# Adaptive dual-programming magnetic soft robot with lifelike behavior

**DOI:** 10.1126/sciadv.aeh1001

**Published:** 2026-11-06

**Authors:** Yanchen Ye, Can Xu, Sen Wang, Weijia Li, RongYe Wu, Hao Wu, Neng Xia, TianYe Yang, Shuang Song, Li Zhang, Yue Dong, Bing Li

**Affiliations:** ^1^ State Key Laboratory of Robotics and Systems (HIT).; ^2^ Guangdong Provincial Key Laboratory of Intelligent Morphing Mechanisms and Adaptive Robots.; ^3^ Key University Laboratory of Mechanism and Machine Theory and Intelligent Unmanned Systems of Guangdong.; ^4^ Harbin Institute of Technology, Shenzhen 518052, China.; ^5^ Department of Mechanical and Automation Engineering, The Chinese University of Hong Kong, Shatin, New Territories, Hong Kong SAR, China.

## Abstract

Magnetic soft robots with embodied intelligence of multimodal motions, environmental perception, and functional decoupling depend on ingenious material arrangement and programmable morphing information introduction. While some efforts have been made, achieving lifelike behaviors for soft robots in system-level strategy remains a notable challenge. Here, we present dual-programming hydrogel-based soft robots (DHSRs) that couple magnetic and anisotropic programming through the spatial arrangement of patterned magnetic skeletons into responsive and structural hydrogel bodies. These DHSRs exhibit programmable exquisite and multicurved structures in three-dimensional (3D) space, environment-adaptive deformation behaviors proved by switching from random jumping on land to regular swimming in water of a fish-like soft robot, and further locomotion-function decoupling capabilities of region-selective cargo delivery in an integrated “locomotion-perception-action” workflow. Our strategy in biomedical potential is exemplified by a ring-like pH-responsive DHSR for gastric ulcer therapy, which integrates multimodal locomotion (rolling, tumbling, and rotating), adaptive matching, and microneedle-mediated penetration. By collaborating with a capsule endoscopy system, we explore the possibility of a wireless and painless diagnostic-therapeutic platform in a pig model. This strategy endows the robot with perception and multitask execution capabilities, highlighting a promising route for the intelligent evolution of soft robots across diverse application domains.

## INTRODUCTION

Living organisms with biological intelligence undertake essential responsibilities in the natural systems, evolving exceptional locomotion and deformation capabilities (*1*–*4*), environment-adaptive behaviors (*5*–*7*), and dynamic exquisite structures (*8*). These serve as long-term aspirations and enable small-scale soft robots to obtain substantial advancement in their intelligence and functions. Magnetically controlled soft robots are particularly attractive owing to their remote actuation modes (*9*, *10*), fast response rate, customized magnetization (*6*, *10*–*12*), and safe interaction with surroundings, highly promoting the promising applications of soft grippers (*1*, *6*, *8*, *11*, *13*–*17*), intelligent electronics (*10*, *18*–*20*), bionics (*16*, *21*–*23*), precision medicine (*12*, *19*, *21*, *24*, *25*), and minimally invasive surgery (*26*–*30*). Current applications are mainly dependent on the programmable structural changes of magnetic soft robots, and some functional units (microstructures, particles, molecules, cells, etc.) are additionally integrated into the body of soft robots to enrich their multifunctionality (*13*, *25*, *31*–*34*). However, achieving lifelike intelligence in system-level strategy remains a notable challenge (*34*–*36*).

The construction of programmable magnetic soft robots capable of transformative morphologies is to arrange the orientations and regions of magnetization profiles throughout the soft matrix (magnetic programming) (table S1). In general, hard magnetic particles of neodymium-iron-boron (*10*, *13*) (NdFeB), chromium dioxide (*11*), nanomagnets (*37*), and nickel nanowires (*16*) are uniformly distributed into silicon elastomers (*13*), adhesive tape (*19*), rigid membranes with soft link (*37*), and other soft polymers (*9*) to create soft robots with customized magnetization, resulting in multiple ingenious origami-like deformations (discrete magnetization) and simple curved shapes (continuous magnetization) (*10*, *11*, *13*, *38*). The pursuit of spatially intricate multicurved structures coupled with magnetized morphing is rarely explored. In addition, the nonresponsive characteristic of soft matrix makes the overall structural changes of soft robots under the sole stimuli of magnetic field, severely restricting their functional decoupling capabilities, environmental adaptability, and perception ability of soft robots that are regulated by spatiotemporally controlling their local deformation when stimulated by multiple factors (*35*).

The introduction of active materials with variable properties endows magnetic soft robots with the capabilities of multiple sensing and local actuation. Liquid crystal elastomers (*39*–*41*), responsive multilayered films (*32*, *42*), and hydrogels (*1*, *43*–*46*) are used as the body materials of soft robots that display transformative shape when response to light (*47*), heat (*46*), and magnetic fields, allowing enhanced environmental perception ability. In particular, hydrogels stand out in constructing soft robots with lifelike behavior due to their three-dimensional (3D) hydrophilic network, tissue-like softness, and responsive elements customizability (*48*, *49*). The environment-adaptive deformation behaviors of hydrogel soft robots, synergized with magnetic actuation, enable their functions decoupling of fast motion and cargo delivery. However, the mutual noninterference of diverse independent functions induced by local deformation of soft robots faces difficulties. Magnetic actuation tends to initiate overall deformation due to the uniform distribution of magnetic particles. In addition, it has been widely accepted that the spatial arrangement of patterned rigid elements within the hydrogels will generate programmable anisotropic deformation of multicurved structures (*16*, *50*). While the rapid sedimentation and agglomeration of magnetic particles (especially for NdFeB) in the hydrogel precursor solution make it challenging with current additive manufacturing techniques and make scalable manufacturing difficult. Thus, a more efficient strategy should be developed to distribute patterned magnetic particles into locally responsive hydrogels to construct magnetic soft robots with programmable exquisite structures and lifelike behaviors.

Here, we address these challenges with a modular dual-programming strategy that allows individual arrangement of magnetic skeletons (patterned NdFeB particles) and hydrogel body modules (Fig. 1). These patterned skeletons are acquired by assembling modular units with and without prescribed magnetization, which simultaneously provide magnetic programming and anisotropic programming information to enrich the deformation structures of hydrogel-based magnetic soft robots. Hydrogels are distributed onto the magnetic skeletons in the form of multiple types, structures, and layers to construct soft robots and argument their perception ability and functions. The deformation and fish-like motion behavior of a soft robot in air and water controlled by the same magnetic field are investigated to prove the environmental adaptability of soft robots. Temperature-, pH-, and ion-sensitive hydrogel units are locally integrated into soft robots to execute the functions of cargo delivery. These functions and the magnetic actuation are determined by different deformations and have no obvious interference with each other, demonstrating the functional decoupling capability of the soft robot. In addition, we develop a ring-like pH-responsive soft robot loaded with microneedles to explore its biomedical application in gastric ulcer therapy. This soft robot uses magnetic field–controlled multimodal motion of rolling, tumbling, and rotating to reach the target region, which further unfolds in the gastric acid to expose microneedles that are used to eradicate and penetrate biofilm for killing bacteria deep inside. As a proof-of-concept demonstration in robot collaboration, capsule endoscope–assisted guidance, observation, and feedback control of soft robots are explored for minimally invasive treatments through in vivo experiments in a pig model. Overall, our strategy revolutionizes soft robots with lifelike behaviors of adaptive deformation and functions decoupling and is expected to advance the development of more intelligent soft robots across multiple fields.

**Fig. 1.**
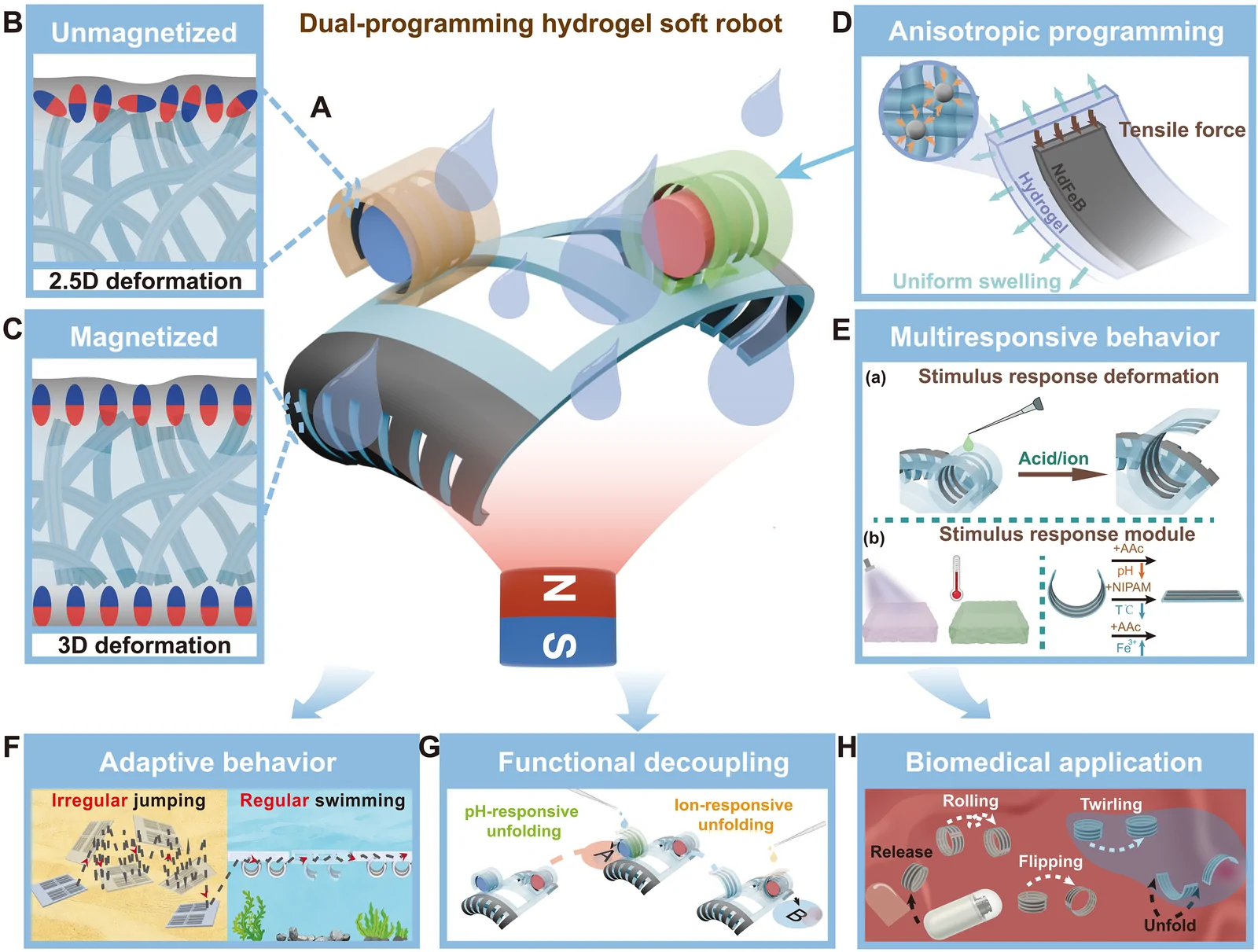
Schematic illustration of the DHSR and its behaviors. (**A**) Overall structure of the dual-programming robot, which can deform and move under humid conditions and external magnetic fields. Specific functional modules exhibit responsive deformation upon environmental stimuli. The robot can simultaneously carry both unmagnetized (**B**) and magnetized magnetic skeletons (**C**) to meet different requirements for deformation and locomotion. (**D**) Schematic of the anisotropic deformation mechanism of hydrogels induced by the rigid magnetic skeleton. (**E**) Functional components composed of various stimuli-responsive hydrogels. (**F**) Environment-adaptive behavior of the DHSR. (**G**) Functional decoupling capacity of the DHSR. (**H**) Biomedical application of the DHSR.

## RESULTS

### Design and fabrication of the dual-programming hydrogel-based soft robot

Figure 2 illustrates the design and fabrication process of the dual-programming hydrogel-based soft robot (DHSR) that requires the distribution of patterned NdFeB particles with programmable magnetization into the hydrogel (fig. S1). In conventional fabrication, the rapid sedimentation of NdFeB particles causes compositional heterogeneity in the flexible matrices of magnetic soft robots and therefore restricts soft robot manufacturing precision in both spatial and temporal dimensions, hindering the robot from achieving precise programmed deformations (Fig. 2A and fig. S2). In addition, the disordered diffusion and agglomeration of NdFeB particles in the flexible matrix directly compromise magnetic patterning fidelity and batch-to-batch reproducibility, which disrupt the formation of complex patterned architectures and magnetization programming (Fig. 2, B and C), thereby limiting the deformation complexity and locomotion performance of robots.

**Fig. 2.**
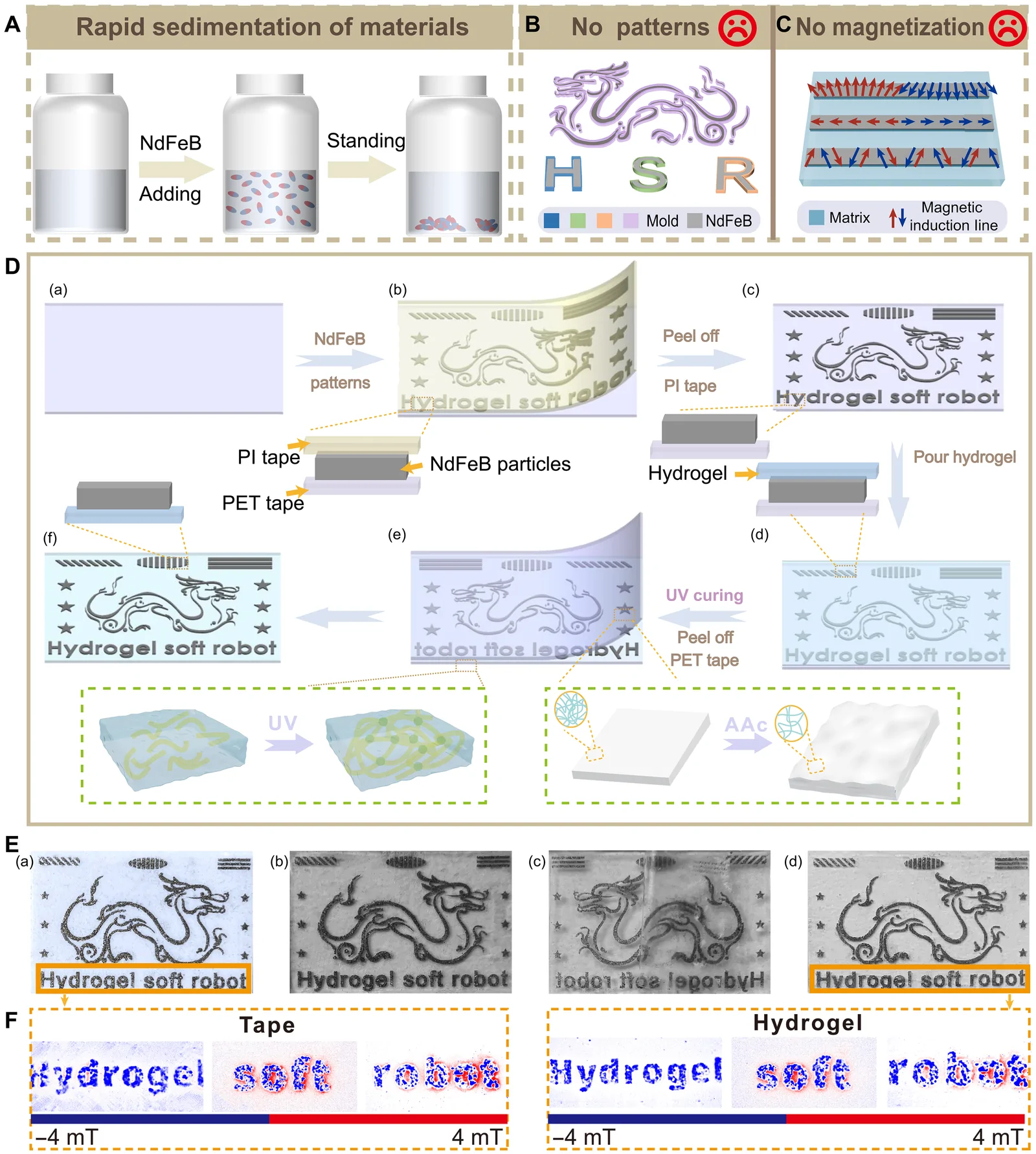
Fabrication process of the DHSR. Conventional fabrication of magnetic soft robots often encounters challenges such as (**A**) sedimentation of magnetic particles, (**B**) inability to achieve patterned distribution of magnetic domains, and (**C**) difficulty in arbitrary programming of magnetic orientations. (**D**) Schematic illustration and (**E**) optical image of the fabrication process of the dual-programming soft robot. (**F**) Magneto-optical microscopy images of the magnetic particles before (left) and after (right) the transfer.

To address these challenges, we propose a simple yet versatile fabrication strategy for hydrogel-based magnetic soft robots that uses NdFeB patterns to mediate programmable anisotropic deformation of the hydrogel. To validate the high patterning resolution and reproducibility of the proposed fabrication strategy, a complex “Loong” pattern is used as a representative example. The detailed fabrication process is illustrated in Fig. 2D. Magnetized NdFeB patterns are first transferred onto a polyimide (PI) adhesive tape (fig. S3), which is then laminated onto the exposed adhesive surface of a polyethylene terephthalate (PET) tape [Fig. 2D (b)]. The magnetization direction and spatial arrangement of NdFeB particles are determined according to the predesigned deformation behavior of the robot [Fig. 2D (c) and fig. S1]. Because of the adhesion differences between the PET and PI tapes, the magnetized patterns are efficiently transferred onto the PET substrate. This transfer method enables the production of magnetic patterns with high precision, uniformity, and reproducibility without the need for costly equipment (figs. S4 and S5 and tables S2 to S5). Furthermore, we have developed a roll-to-roll patterning and transfer system to extend this approach to automated and scalable fabrication (fig. S6 and movie S1). Subsequently, the hydrogel prepolymer solution is poured onto the PET tape to cover the NdFeB patterns [Fig. 2D (d)], and its thickness is precisely defined using spacers and glass slides (fig. S7). Following ultraviolet (UV) photopolymerization, the hydrogel network gradually densifies, enabling the hydrogel to tightly encapsulate the NdFeB particles. Upon the peeling process [Fig. 2D (e)], the resulting interfacial bonding strength between the hydrogel and the particles exceeds the adhesive force between the particles and the PET tape (fig. S8A). In addition, acrylic components within the hydrogel react with the adhesive layer of the PET tape, causing the network within the adhesive layer to become sparse and thereby reducing its adhesion to the particles. These two effects ensure that the PET tape can be smoothly peeled off from the cured hydrogel surface while preserving the magnetic particle patterns intact on the hydrogel [Fig. 2D (e), fig. S8B, and movie S2]. The resulting hydrogel can be further tailored to the desired shape for optimizing the target deformation.

**Fig. 3.**
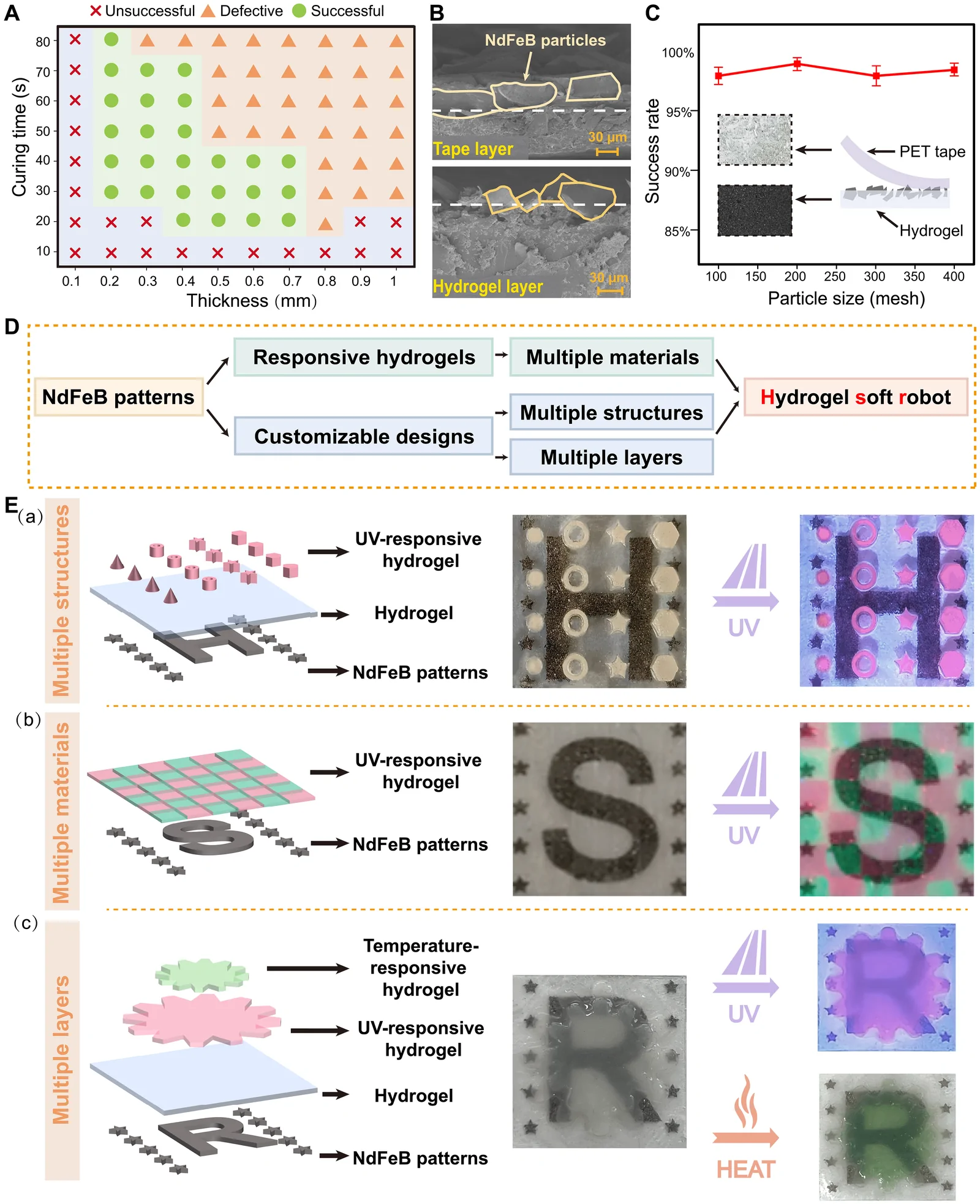
Analysis of fabrication parameters and functional design of the robot. (**A**) Effects of different hydrogel thicknesses and curing times on robot fabrication. (**B**) Cross-sectional SEM images of magnetic particles on tape (top) and within hydrogel (bottom). (**C**) Transfer efficiency of magnetic particles with different sizes. Error bars represent the SD (*n* = 3). (**D**) Design concept of the hydrogel-based robot. (**E**) Responsive hydrogel structures with various patterns and structural arrangements.

Figure 2E systematically presents the sequential fabrication stages, including hydrogel casting, polymerization, PET tape removal, and the final product after separation. The NdFeB particles remain entirely embedded in the hydrogel surface. Furthermore, magnetic-optical microscopy images (Fig. 2F) of the “hydrogel soft robot” pattern, both before and after separation, confirm that the alignment and magnetization intensity of the magnetic particles remain mostly unaffected. This result verifies the stability of the magnetic response and hydrogel-NdFeB interface (figs. S9 to S12) in the fabricated hydrogel-based soft robot, ensuring its reliable deformation and locomotion performance. However, the robot should be prevented from long-term storage in alkaline environments or in air, as this may affect its stability and mechanical properties (fig. S13).

### Efficiency of magnetic particle transfer and modular functional integration

In the fabrication process of hydrogel-based robots, the efficient integration of magnetic particles and the customizable design of functional modules are critical to ensuring the stability and versatility of the robots. We systematically analyze the parameters affecting the particle transfer efficiency. The hydrogel layer thickness and UV exposure time are the dominant factors governing transfer performance (Fig. 3A). With increasing UV exposure duration, the crosslinking density and mechanical strength of the hydrogel progressively increase (fig. S14). However, prolonged exposure promotes adhesion between the hydrogel network and the adhesive layer, leading to undesired residue on the hydrogel surface after peeling (fig. S15B). In addition, an excessively thick hydrogel layer hinders the penetration of light during photopolymerization, resulting in incomplete crosslinking at the bottom layer and a loose polymeric network that fails to firmly anchor the hard magnetic particles (fig. S14B). Experimental results indicate that the parameter combinations within the green region in Fig. 3A are suitable for fabricating hydrogel-based robots. Among these, the optimal parameters for robot fabrication are a hydrogel layer thickness of 0.3 mm and a UV curing time of 40 s, as these parameters endow the robots with both high particle transfer efficiency and robust mechanical performance. Unless otherwise specified, all subsequent robot fabrications are carried out under these optimized conditions. To further validate the reliability of the fabrication strategy, we visually compare the adhesion behavior of NdFeB particles on the surfaces of PET tape and hydrogel. Cross-sectional scanning electron microscopy (SEM) images (Fig. 3B) reveal tight NdFeB particles embedded on both substrates, confirming the strong interfacial adhesion achieved by this process. In addition, the main body of magnetic particles is encapsulated within the hydrogel after the transfer process, further confirming the effectiveness of the fabrication strategy. Quantitative analysis of the residual particles on the PET surface demonstrates consistently high transfer efficiency across various particle sizes (Fig. 3C).

Functional design constitutes another key aspect in the fabrication of hydrogel soft robots. The NdFeB patterns endow the DHSRs with a high degree of magnetic programmability. Meanwhile, the intrinsic customizability of hydrogels allows them to respond to diverse environmental stimuli. When combined with versatile templating and 3D printing methods, this strategy enables the creation of functional modules featuring multiple structures, materials, and layers, thereby greatly enriching the functional diversity of hydrogel soft robots (Fig. 3D). Specifically, incorporating UV- or temperature-responsive powders into the hydrogel matrix imparts distinct color-changing behavior under corresponding stimuli (Fig. 3E). Through a template-assisted strategy (fig. S16), multiple UV-responsive color-changing 3D hydrogel structures (e.g., microneedles, hollow cylinders, pentagrams, polygonal prisms, and gears) are constructed on the surface of magnetic particle–embedded hydrogels [Fig. 3E (a)]. Since the responsive hydrogels preserve their interconnected polymeric network, the high magnetic particle transfer efficiency remains uncompromised. Furthermore, sequential photopolymerization combined with customized template designs enables the realization of more sophisticated multiresponsive architectures, allowing hydrogels with distinct color-changing properties to be integrated within single (fig. S17) or multilayer configurations [Fig. 3E (b and c)]. By further tuning the concentration of responsive components, the optical transparency of the hydrogel can be optimized to customize the visibility of the underlying magnetic patterns during color transition (fig. S18). In addition, the introduction of specific functional groups allows the hydrogels to undergo stimulus-induced shape deformation, further enriching the functional complexity and advancing the soft robot toward intelligent lifelike behaviors.

### Dual-programming deformation and environmental adaptability of the DHSR

Patterned NdFeB particles with programmable magnetization can serve as rigid skeletons to mediate anisotropic deformation of hydrogels, endowing hydrogel-based robots with both magnetic programming deformation and anisotropic deformation capabilities. Under an external magnetic field, the preprogrammed magnetic domain orientations within the skeleton align with the magnetic field direction, guiding the predesigned patterned structures (a windmill, a flower, and a turtle) to undergo coordinated deformation [Fig. 4, A (a), B (a), and C (a)]. Meanwhile, the patterned rigid skeleton imposes asymmetric constraints on the hydrogel, where the embedded magnetic particles restrict the swelling of the hydrogel network on the patterned side. Such differential expansion drives the entire layer to bend toward the patterned side upon water absorption, allowing precise control of anisotropic deformation through structural design [Fig. 4, A (b), B (b), and C (b)]. The influence of geometric parameters on anisotropic deformation is further examined by varying the magnetic stripe width and hydrogel strip length (fig. S19). The results indicate that wider stripes lead to a greater bending curvature (fig. S19B), while the length of the hydrogel strip shows only a weak correlation with anisotropic deformation (fig. S19A). Likewise, the curvature of helical deformation increases with the width of oblique stripes (fig. S20), highlighting the critical role of magnetic pattern geometry in regulating programmable morphing. This approach overcomes the limitations of conventional magnetic programming techniques in achieving complex multicurvature deformation, thereby enhancing the morphological complexity of magnetic soft robots. Since the two programming modes can be independently controlled, they can be coupled to achieve more complex and synergistic deformation behaviors [Fig. 4, A (c), B (c), and C (c)]. The above results reveal that swelling-induced stress primarily determines the adaptive morphology and curvature configuration of the robot, whereas magnetic torque mainly governs dynamic locomotion and directional actuation. The dual-programming strategy not only expands the design flexibility for constructing complex morphologies of soft robots but also paves a new way for their applications in multienvironment task execution.

**Fig. 4.**
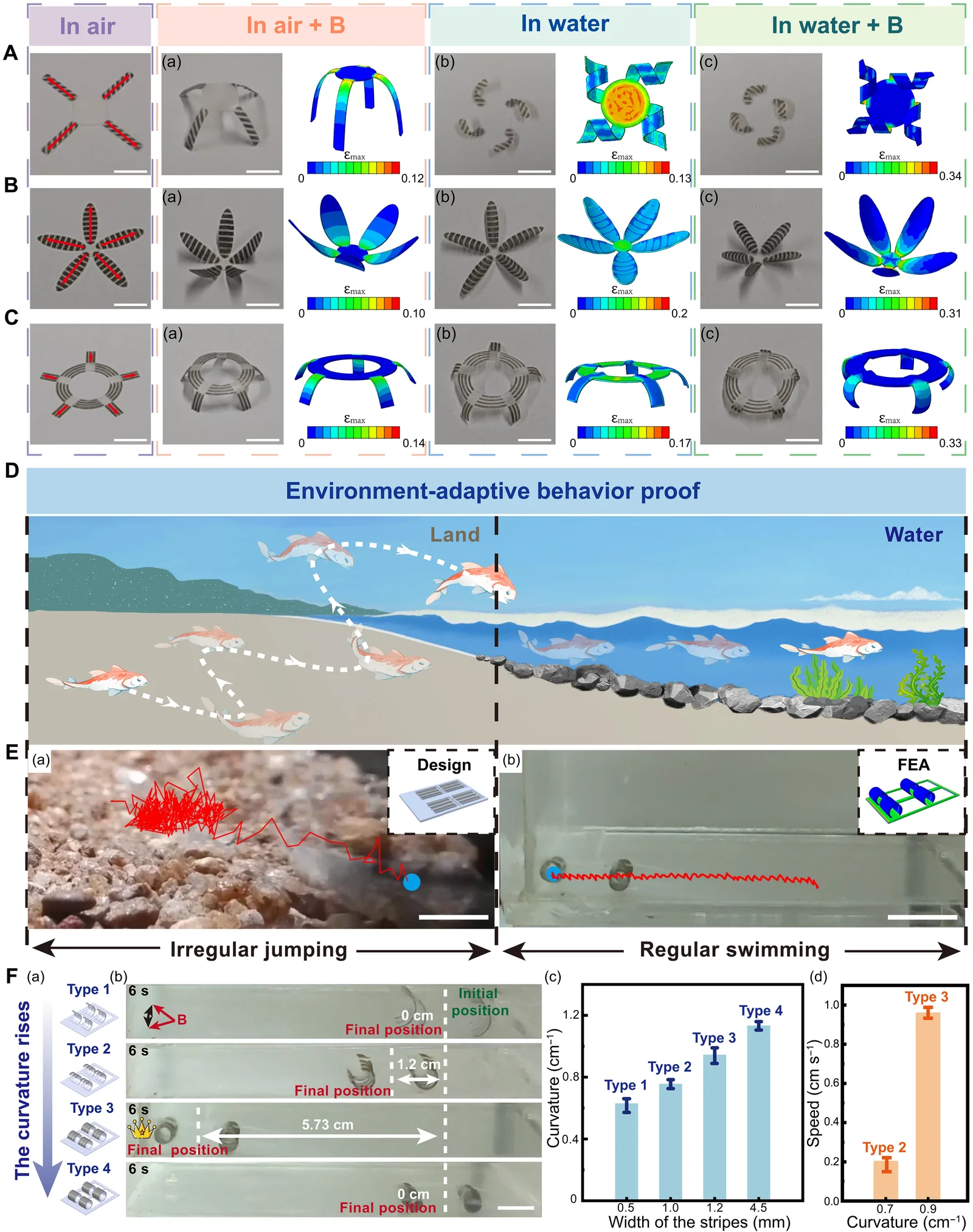
Stimulus-responsive deformation and environmental adaptability of the DHSR. (**A** to **C**) Deformation behavior of DHSR with three different types of striped magnetic skeletons (diagonal, horizontal, and vertical) before and after exposure to external magnetic field, water environment, and combined conditions, along with corresponding simulation results (*10*, *44*). [Scale bar, 1 cm; color bar, range of maximum principal strain (logarithmic strain)]. (**D**) Schematic illustration of different locomotion modes of fish on land and in water. (**E**) Locomotion trajectories of a four-fin robot in simulated land and water environments (inset shows the design and simulation results of the robot). (**F**) The effect of the magnetic skeleton stripe width on the robot’s locomotion parameters in water. Error bars represent the SD (*n* = 3).

Inspired by the environment-adaptive behaviors of fish in nature during locomotion, we further explore the bioinspired behaviors of the DHSR. As shown in Fig. 4D, a fish exhibits irregular thrashing on land; however, upon entering water, coordinated tail oscillation and fin undulation enable it to swim flexibly along a regular trajectory. To emulate this environment-adaptive locomotion, we fabricate a four-fin fish-like soft robot, whose “fins” are patterned with vertically aligned NdFeB stripes on one side. Upon water absorption, these stripes induce inward bending of the hydrogel, allowing the robot to mimic fish-like swimming behaviors. As illustrated in Fig. 4E, under an oscillating magnetic field in the *z* direction, the locomotion trajectory of the robot on land exhibits high randomness [Fig. 4E (a)]. After undergoing environment-adaptive deformation, the same magnetic stimulation causes the four curled fins to oscillate in a coordinated manner, resulting in straight-line locomotion [Fig. 4E (b), fig. S21, and movie S3]. This swimming behavior of the robot is attributed to its four dual-programming fins, which are magnetized along the longitudinal axis of the stripes and deploy in alignment with the magnetic field direction, thereby generating regular frictional forces with the contact interface to propel the robot forward. To investigate the effect of stripe width on anisotropic fin deformation and overall locomotion, we design four robots with stripe widths of 0.5, 1.0, 1.2, and 4.5 mm [Fig. 4F (a and c)]. Simulation results show that increasing the stripe width enhances the fin curling curvature (fig. S22). The predefined anisotropic programming regulates swelling-induced shape evolution, thereby reconfiguring the spatial geometry of the premagnetized skeleton and the relative orientation of its magnetic moments, which ultimately alters the magnetic actuation mode. When tested in the same environment for 6 s, robots with wider stripes exhibit greater fin deformation, which produces larger opening and closing amplitudes under identical magnetic field stimulation [Fig. 4F (b)]. This enhanced deformation generates stronger propulsive forces, increasing the robot’s swimming speed, with the maximum velocity reaching 0.955 cm s^−1^ [Fig. 4F (d)]. Notably, beyond a critical curvature, the magnetic field is insufficient to fully open the curled fins, limiting their oscillation amplitude and reducing propulsion, which ultimately prevents the robot from overcoming water resistance to move forward.

### Locomotion-function decoupling strategy

Owing to the challenge of autonomously controlling their motion joints, traditional soft robots struggle to achieve effective decoupling between locomotion and functionality, thereby restricting their capability for multitask execution. To address this limitation, we introduce the dual-programming deformation strategy in hydrogel-based soft robots, in which several actuation modes are independently regulated. When distinct stimulus-responsive hydrogels are integrated into specific modules, localized deformations can be triggered under corresponding external stimuli. This multimode actuation mechanism enables effective locomotion-function decoupling, endowing the hydrogel-based soft robot with lifelike adaptability for diverse operational scenarios. Building on the original fabrication method, we further propose a double-sided patterning programming strategy to enhance the spatial complexity of shape morphing (Fig. 5A and fig. S23). Specifically, patterned NdFeB particles are incorporated on both sides of the same region or on opposite sides of different regions within the hydrogel matrix, depending on the desired deformation behavior. This advanced strategy offers two key advantages: (i) Regions of the hydrogel patterned with magnetic skeletons on both sides exhibit enhanced mechanical strength owing to dual-layer magnetic reinforcement [Fig. 5A (a)]; (ii) the introduction of bidirectional anisotropy facilitates more complex multicurved deformations, such as asymmetric or opposite side bending in water [Fig. 5A (b)].

**Fig. 5.**
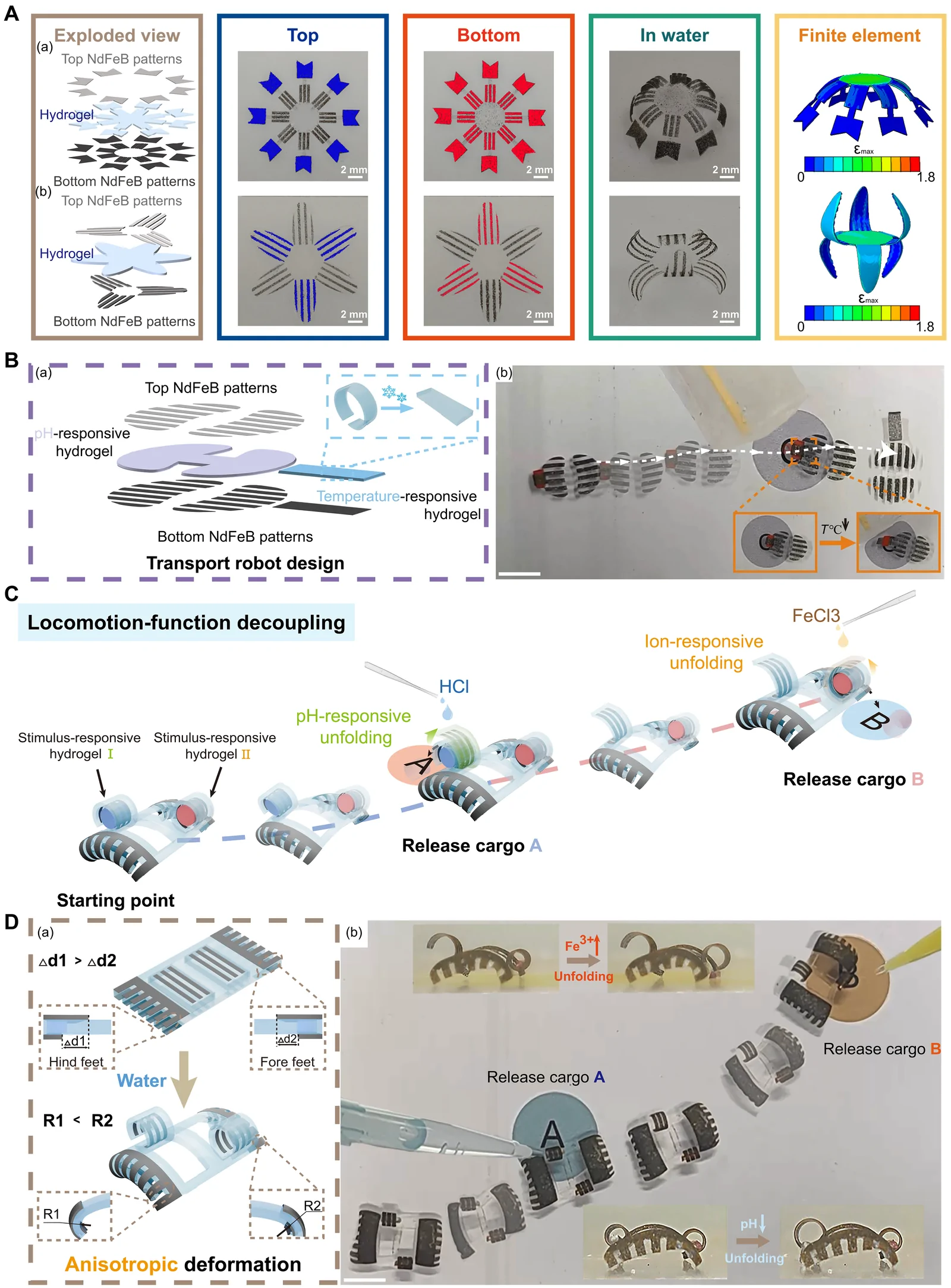
Locomotion-function decoupling applications of the DHSR. (**A**) Design schematic of the double-sided magnetized skeleton robot, along with optical images and simulation results before and after water-induced swelling and deformation. (**B**) Design diagram and locomotion trajectory of the robot for single-site cargo release. (**C**) Schematic illustration of the working process of the robot for multisite cargo release. (**D**) Demonstration of the robot before and after deformation upon water exposure and snapshots of the locomotion and working process (inset shows the cargo release process). Scale bars, 1 cm. Color bar, range of maximum principal strain (logarithmic strain).

To validate the locomotion-function decoupling capability, we develop a DHSR designed for cargo transport (Fig. 5B). The robot body, serving as the locomotion module, is composed of a pH-responsive hydrogel coated on both sides with premagnetized NdFeB stripe patterns to enhance magnetic response and mechanical robustness. The grippers, acting as the function modules, are fabricated from a temperature-responsive hydrogel with unmagnetized NdFeB strips attached to their inner surfaces, only inducing anisotropic deformation. This design achieves decoupling between the robotic body (locomotion module) and gripper (function module) in actuation and task execution. Under an alternating magnetic field, the body undergoes cyclic bending to propel the robot forward, while the unmagnetized gripper remains unresponsive to the magnetic field. Upon reaching the target site, localized cooling gradually decreases the temperature, inducing a temperature-responsive deformation that actuates the gripper to release the cargo. Following delivery, the magnetic field is reapplied to drive the robot away, completing the full transport cycle (movie S4).

The locomotion-function decoupling strategy further imparts the DHSR with sequential actuation capability, providing a promising approach for multitasking manipulation (Fig. 5C). Building on this principle, we develop a DHSR capable of releasing cargo at multiple sites. The robot’s feet are patterned with asymmetrically distributed magnetized NdFeB particles, which provide enhanced mechanical strength and enable one-sided bending in water [Fig. 5D (a)]. Upon undergoing anisotropic deformation, the robot’s feet arch upward, reducing surface contact and consequently minimizing friction to facilitate smoother locomotion. To enhance directional locomotion, asymmetric design differences are introduced between the fore and hind feet. Specifically, the magnetic skeleton pattern difference (*∆d1*) on the hind feet is greater than that on the fore feet (*∆d2*), resulting in a larger bending amplitude of the hind feet. This asymmetric design creates a friction gradient, where the forefeet experience lower frictional resistance than the hind feet, thereby promoting forward propulsion under localized magnetic stimulation. The two hydrogel-based grippers are coated with nonmagnetized NdFeB vertical stripe patterns on their inner sides, allowing them to bend inward upon swelling yet remain unaffected by magnetic actuation. To impart chemical adaptability, acrylic acid (AAc) and methacrylic acid (MAAc) monomers are copolymerized into the hydrogel network, introducing abundant anionic pendant (carboxylic) groups that endow the grippers with pH and ion responsiveness. When the environmental pH decreases or cationic solutions are added, the anionic carboxyl groups on the polymer side chains tend to bind with H^+^ or cations via protonation or ionic complexation (fig. S24). This reduces the electrostatic repulsion between molecular chains, leading to the contraction of the hydrogel network and thus triggering gripper unfolding (fig. S25). By adjusting the concentration of AAc, the response characteristics of the two hydrogel grippers to distinct stimuli are tailored. The left gripper contains a higher AAc concentration than the right counterpart, rendering it more sensitive to pH changing in the environment, whereas the right gripper exhibits enhanced responsiveness to ionic stimuli.

As illustrated in Fig. 5D (b), after initial swelling-induced anisotropic deformation, the robot is magnetically actuated along a preprogrammed trajectory to site A. Upon magnetic field removal, localized addition of hydrochloric acid near the left gripper triggers a pH-responsive deformation to release the cargo, while the right gripper remains unresponsive. Subsequently, the magnetic field is reactivated to drive the robot toward site B. In this region, FeCl_3_ solution is locally applied near the right gripper, inducing Fe^3+^-responsive deformation and the second cargo release (movie S5). This demonstration showcases the capability of soft robots for spatiotemporal control and multisite actuation, highlighting great potential for multitask execution across diverse environments.

### The DHSR for the treatment of gastric diseases

For gastric diseases, localized and on-demand drug delivery using soft robots can effectively reduce systemic side effects and inefficient drug utilization associated with conventional oral or injectable therapies (*19*, *32*, *51*, *52*). However, the complexity of the in vivo environment and constrained operational space poses technical challenges for localized drug delivery, representing a key focus in the community of soft robotics. The environment-adaptive deformation of the DHSR holds promise for addressing this challenge. Both the biocompatible hydrogel matrix, exhibiting tissue-like mechanical compliance, and the NdFeB particles modified with a silica coating exhibit favorable biosafety, laying a foundation for the in vivo operation of the DHSR (figs. S26 and S27).

To validate the potential of DHSRs as minimally invasive tools for in vivo biomedical applications, we perform ex vivo gastric experiments. Leveraging the aforementioned modular integration strategy, a compact DHSR (unfolded length, 20 mm; width, 5 mm; coiled diameter, 6 mm; fig. S28) integrated with drug-loaded microneedles (fig. S29) is developed, which exhibits precise navigation and targeted drug delivery capabilities (Fig. 6A). Vertically aligned NdFeB stripe patterns enable the robot to curl from an elongated shape into a loop upon swelling [Fig. 6A (b)], thereby not only protecting the microneedles from mechanical damage but also preventing unintended injury to surrounding tissues during locomotion. In addition, microneedles can effectively penetrate biofilms, thereby enhancing the efficiency of targeted drug delivery (fig. S30). Actuated by an external magnetic field, the robot can traverse gastric tissues with high precision and adapt to the complex topography and fluidic environment of the stomach by swiftly switching multiple locomotion modes. As the robot navigates from the gastric fundus to the lesion site, it exhibits three typical locomotion modes. In relatively flat regions, the robot traverses rapidly via rolling locomotion under a rotating magnetic field in the *xzxz* plane [Fig. 6B (a)], where *Z* is the outward normal of the gastric wall surface. Reduced friction due to gastric mucus, however, hinders the robot’s effective locomotion over undulating terrains, prompting a switch to flipping mode to overcome higher obstacles [Fig. 6B (b)]. Upon reaching the gastric acidic environment, the robot autonomously adjusts its posture under buoyancy and continues forward via a twirling locomotion under a rotating magnetic field along the *Z* axis [Fig. 6B (c)]. During prolonged exposure to the gastric acid, the AAc components within the hydrogel enable the robot to undergo environment-adaptive deformation, with its curvature decreasing from 0.319 mm^−1^ initially to 0.205 mm^−1^ after 19 min (Fig. 6C and fig. S31), thereby exposing the drug-loaded microneedles. Ultimately, the robot unfolds into a semicircular configuration, facilitating efficient obstacle climbing and conformal adhesion to the gastric folds. Figure 6D demonstrates that the robot remains stable anchoring to the lesion site under the magnetic actuation, enabling targeted microneedle insertion and drug release (fig. S32; the complete process of this experiment is shown in movie S6). Leveraging the dual-programming strategy, the soft robot can sense environmental changes at the physical intelligence level and undergo adaptive deformation, thereby ensuring effective targeted delivery and providing a new tool and insight for the development of intelligent medicine.

**Fig. 6.**
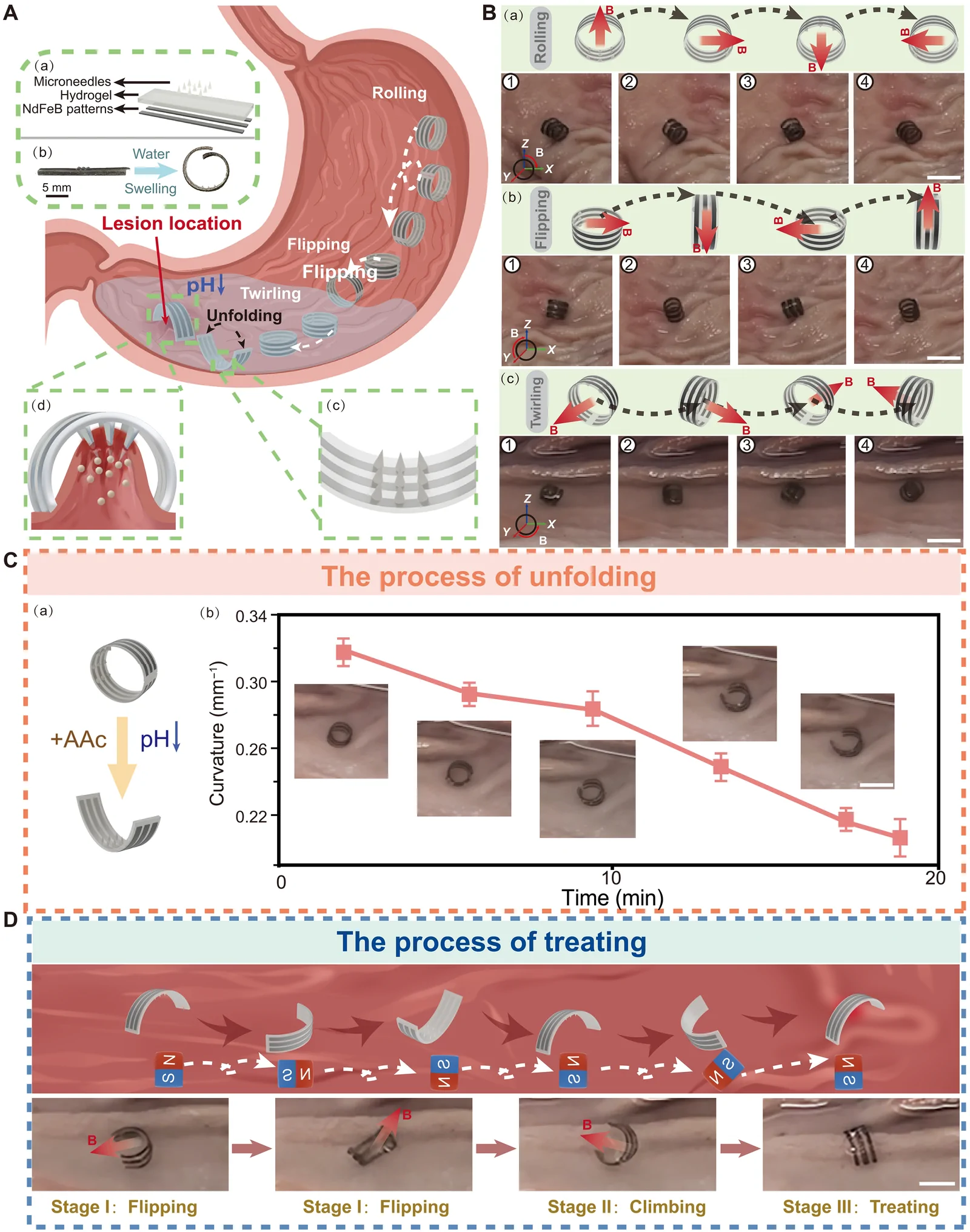
Localized therapy in the stomach using the hydrogel microneedle robot. (**A**) Design schematic and working process illustration of the robot. The stomach created in BioRender. Ye, Y. (2026), https://BioRender.com/rstkv5b. (**B**) Three locomotion modes of the robot inside the stomach. (**C**) Schematic of pH-responsive deformation of the DHSR and the corresponding evolution of curvature during unfolding in an acidic environment. Error bars represent the SD (*n* = 3). (**D**) Images illustrating the localized therapeutic process of the robot. Scale bar, 1 cm.

### Integrated therapy combining the DHSR and capsule endoscopy

Despite advances in the biomedical application of soft robots, translating these medical devices from bench to bedside remains challenging. Traditional gastrointestinal endoscopic techniques, such as gastroscopy and colonoscopy, have long been regarded as the gold standard for the diagnosis of gastrointestinal diseases (*53*, *54*). However, their invasive operation often causes patient discomfort, requires anesthesia, and leaves diagnostic blind regions. In recent years, the successful clinical adoption of magnetically controlled capsule endoscopes has enabled painless, wireless, and anesthesia-free diagnostics. Building on this progress, our previous work proposed that integrating soft robots with capsule endoscopes holds promise as an ideal painless tool for treating gastrointestinal ulcers (*19*, *55*, *56*).

Here, we combine capsule endoscopy with the DHSR, allowing the diagnostic and therapeutic modules to enter the body via a single ingestion, thus achieving simultaneous wireless lesion detection and localized treatment (Fig. 7A). The capsule endoscope measures 11.8 mm in diameter and 31.9 mm in length (fig. S33), exceeding the dimensions of the soft robot. To ensure protection during ingestion, the DHSR is encapsulated in a pH-sensitive medical coating, which is mounted at the posterior end of the capsule (Fig. 7B). An axially magnetized permanent magnet embedded inside the capsule as an actuation module enables rotation and translation under an external magnetic field. The control system block diagram of the capsule endoscope is shown in Fig. 7C. Image data captured by the onboard complementary metal-oxide semiconductor camera is transmitted via an internal RF coil to an external receiving antenna system (Fig. 7D), which stores the data on a memory card for subsequent image retrieval and diagnostic analysis on a computer.

**Fig. 7.**
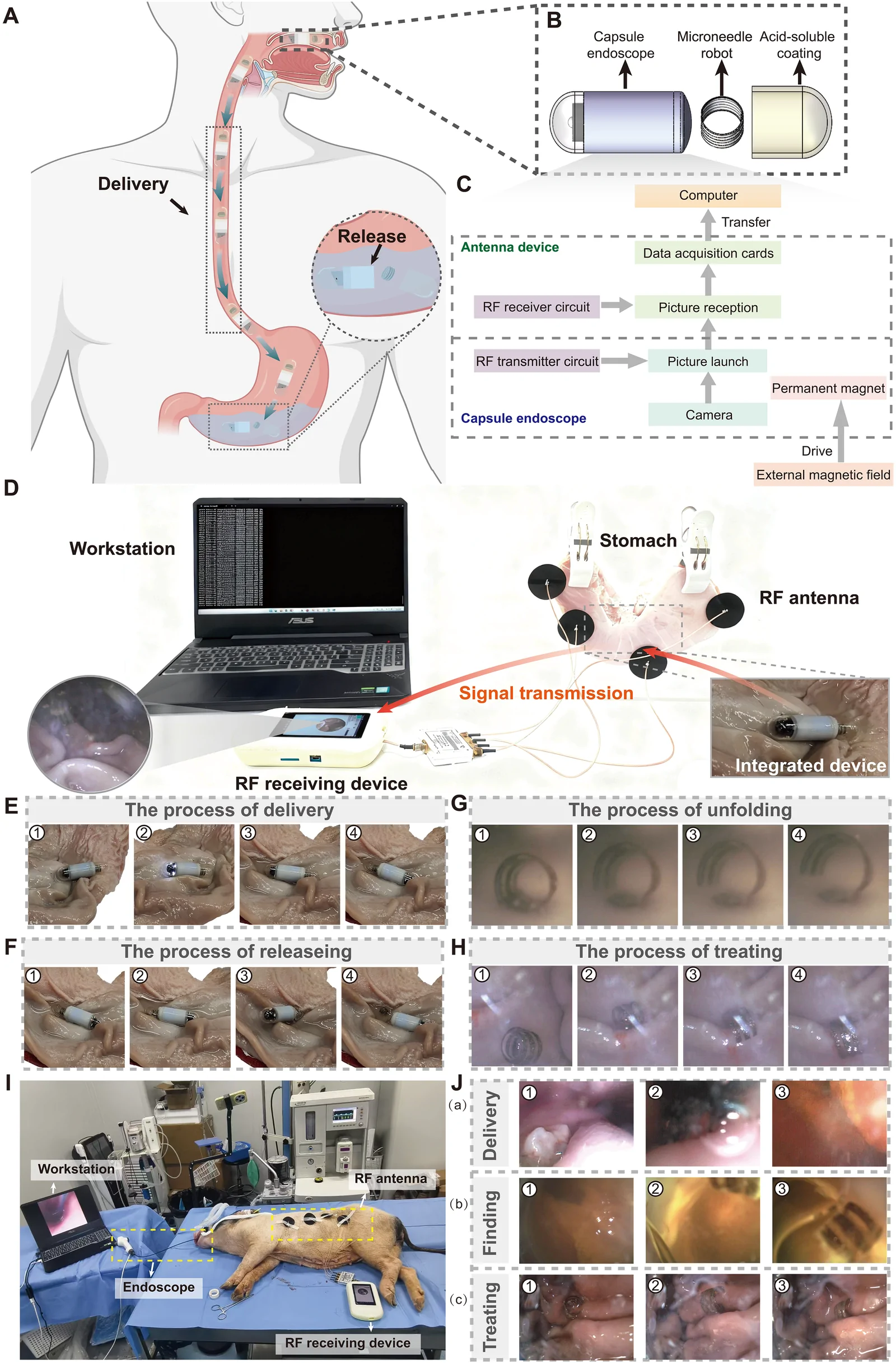
Integrated therapeutic system of the DHSR and capsule endoscope. (**A**) Conceptual illustration of the integrated therapeutic approach combining the robot with the capsule endoscope. Created in BioRender. Ye, Y. (2026), https://BioRender.com/rstkv5b. (**B**) Exploded view of the integrated system. (**C**) Schematic of the image transmission mechanism of the capsule endoscope. (**D**) The experimental setup is shown: images captured by the capsule endoscope are transmitted via antenna to the receiver. (**E**) Delivery process of the integrated system. (**F**) Release process of the DHSR. (**G**) PH-responsive unfolding process of the robot. (**H**) The therapeutic process of the robot. (**I**) Overall layout of the capsule endoscope device, the catheter endoscope, and the auxiliary equipment for the in vivo porcine surgery. (**J**) The processes of capsule endoscope delivery, localization of the DHSR, and the DHSR treatment within the porcine stomach.

To validate the diagnostic-therapeutic performance, we simulate the locomotion, diagnosis, and operation of the integrated device within an ex vivo enclosed gastric environment. Driven by a gradient magnetic field parallel to the capsule’s longitudinal axis, the device moves from the gastric fundus to the acidic antrum. Upon reaching the target site, the capsule reorients to ensure adequate exposure of the pH-sensitive coating to gastric acid (Fig. 7E). After partial dissolution of the coating, leveraging friction from the irregular gastric surface, the capsule endoscope uses oscillatory motion to fully dislodge the coating, enabling successful release of the DHSR. Differential magnetic actuation between the capsule and the robot facilitates their separation, allowing the capsule to navigate toward an optimal observation site (Fig. 7F). The integrated imaging module enables the continuous capture of high-resolution color images, recording the unfolding of the DHSR with a curvature change from 0.32 to 0.20 mm^−1^ within ∼15 min, consistent with previous experiments (Fig. 7G). As the capsule repositions during imaging, the expanding field of view reveals the DHSR rolling toward the ulcer and anchoring onto the lesion site for localized therapy (the complete process of this experiment is shown in Fig. 7H and movie S7).

Beyond the ex vivo experiment, the in vivo porcine gastric model further confirms the translational potential of the integrated capsule-DHSR platform (Fig. 7I and movie S8). During the procedure, the platform is delivered through an over-tube via the oral route into the stomach and subsequently guided to the target region under magnetic actuation. The capsule endoscope records the entire therapeutic workflow in vivo, including transesophageal delivery [Fig. 7J (a)], the DHSR release in the acidic gastric environment [Fig. 7J (b)], and magnetically guided climbing and anchoring of the DHSR onto the target site along the gastric wall [Fig. 7J (c)]. Owing to the limited imaging frequency and restricted visual range of the commercial capsule endoscope used in this study, an electronic gastroscope is additionally used to provide real-time visualization, facilitating precise magnetic control and positional calibration. Encapsulated within the pH-sensitive coating at the posterior end of the capsule, the DHSR is successfully released upon exposure to gastric acid and magnetically guided to the target site for localized treatment. These in vivo results confirm that the proposed integrated diagnostic-therapeutic platform enables flexible locomotion navigation and effective lesion-targeted drug delivery within the confined, narrow, and structurally complex gastric environment.

The integration of capsule endoscopy with soft robotic technologies holds great promise for the development of wireless intelligent diagnostic and therapeutic platforms. However, the realization of a fully integrated diagnostic-to-therapeutic workflow within an in vivo gastric environment remains challenging. Current limitations stem from restricted visual range, limited motion precision, and insufficient controllability of robot release. Future efforts may focus on optimizing the structural robustness and deployment reliability of therapeutic soft robots, alongside developing more efficient coordination between the capsule and the therapeutic unit, toward truly noninvasive, intelligent, and efficient precision therapy for gastrointestinal diseases.

## DISCUSSION

The development of magnetically controlled intelligent soft robots with lifelike environmental perception, multimodal locomotion, and motion-decoupled multifunctionality holds great promise to advance the widespread application of soft robotics across scientific and engineering fields. Realizing this potential requires the coordinated optimization of material properties and actuation strategies under the constraints of miniaturized designs. Although notable progress has been made in material design, actuation mechanisms, and structural innovation, challenges remain in endowing small-scale soft robots with environmental responsiveness and achieving decoupling between locomotion and functional execution.

In this study, we propose a modular dual-programming strategy that introduces an innovative fabrication method to integrate magnetically programmed skeletons with responsive hydrogel modules, enabling the robot to achieve environment-adaptive deformations and locomotion-function decoupling. This method circumvents the need for direct dispersion of NdFeB particles in hydrogel precursors, ensuring precise magnetic distribution and structural integrity. Magnetic particles are efficiently transferred into hydrogel matrices according to predesigned patterns, thereby preventing particle sedimentation and agglomeration, which is an enduring challenge in magnetic soft robotics. The ability to embed magnetic domains with precise spatial arrangement enables independent programming of magnetic and anisotropic deformations within complex 3D hydrogel geometries. Furthermore, the compositional adjustability of hydrogels enables the preparation of diverse responsive modules (e.g., temperature-, pH-, and ion-sensitive types), which can be flexibly integrated into robotic architectures through templating and 3D printing methods. This modular fabrication framework endows intelligent soft robots with sophisticated architectures and lifelike behaviors and establishes a scalable platform for constructing multifunctional assemblies.

The premagnetized patterned magnetic skeleton enhances the shape-morphing capability of the hydrogel-based robot, exhibiting dynamic lifelike aesthetic. A series of bioinspired robots is designed to demonstrate their complex deformation behaviors under external magnetic stimulation, water environment, and their coupled effects. To achieve more sophisticated asymmetric deformations and improved mechanical robustness, a double-sided programming technique is further developed. Compared with conventional single magnetic programming, this dual-programming design enables diverse deformation modes across varying environments and realizes multicurved morphing behaviors. Three distinct finite element modeling demonstrates that the simulated results are identical to experimental observations, validating both the reliability of the fabrication strategy and the deformation fidelity of the dual-programming soft robots.

Such anisotropic and magnetic programming information not only endows DHSR with environment-adaptive capabilities but also facilitates the decoupling of actuation and functional behaviors. As a proof of concept, a four-fin soft robot is developed to demonstrate its environmental adaptability by emulating the locomotion characteristics of fish. The robot exhibits disordered motion on land, whereas in water, it transforms into a shape of four fins that promote coordinated locomotion, highlighting its capability for environmental adaptation. In multitask execution scenarios, the DHSRs exhibit distinct motions (e.g., swimming, grasping, or releasing), depending on their structural design, while accommodating diverse hydrogel types (e.g., temperature, pH, and ion sensitive) and layer configurations. This functional decoupling, a distinctive trait of lifelike systems, expands the operational scope of soft robots beyond mere motion to integrated “locomotion-perception-action” workflows, providing a paradigm for designing soft robotic systems.

The biomedical potential of our strategy is exemplified by a ring-like pH-responsive DHSR for gastric ulcer therapy, which integrates magnetic multimodal locomotion (rolling, tumbling, and rotating) and microneedle-mediated biofilm penetration. This proof of concept demonstrates three critical advantages for clinical translation: First, the magnetic controlled targeted navigation addresses the challenge of accessing localized lesions in the gastrointestinal tract; second, acid-triggered adaptive deformation leverages the physiological environment for on-demand therapy, enhancing drug delivery efficiency while minimizing off-target effects; third, the drug-loaded microneedles can penetrate biofilms for deep-seated therapy, addressing a critical barrier in treating refractory infections. Furthermore, the integration of capsule endoscopy for real-time guidance, observation, and feedback further showcases the system’s capabilities for collaborative, minimally invasive therapy, highlighting our strategy’s potential to bridge the gap between soft robotics and translational medicine.

Overall, the key innovation of our work lies in emphasizing the “lifelike adaptability” of soft robots, where adaptive deformation and function decoupling are seamlessly integrated with biomedical practicality. Despite these advancements, several aspects hold promise for advancing this technology. The dual-programming strategy demonstrated in this work enables the transformation of 2D planar structures into 3D complex architectures. Future efforts may focus on implementing more sophisticated programming transformations on prefabricated 3D constructs, which will further expand the morphological diversity and functional versatility of soft robots. Regarding biomedical applications, while we have combined our localized therapeutic module with capsule endoscopy, realizing full-process operations in vivo environment of the stomach requires additional modules such as visual feedback and path planning. The integration of flexible sensing, closed-loop control, and machine learning technologies holds promise for enabling soft robots to progressively realize genuine, lifelike intelligence. Incorporating real-time tracking and navigation capabilities in such confined environments will be crucial for completing diagnostic and therapeutic tasks more effectively. Integrating machine learning algorithms to enable real-time feedback control (e.g., adjusting locomotion mode based on environmental cues detected by capsule endoscopy) could enhance robotic intelligence. Expanding the modular library to include conductive hydrogels or piezoelectric materials may add sensing and energy-harvesting capabilities. In addition, scaling down the dimensions of robots could enable targeted therapy in narrower regions, such as gastrointestinal tract, bile ducts, blood vessels, auditory meatus, and nasal cavity.

## MATERIALS AND METHODS

### Material preparation

AAc (99%), MAAc (99%), ethyl lactate (98%), and *N*,*N*-dimethylformamide (DMF; 99.9%) are purchased from Shanghai Aladdin Biochemical Technology Co. Ltd. Polyvinylpyrrolidone (PVP; average molecular weight ≈ 1,300,000 g mol^−1^) is obtained from Shanghai Macklin Biochemical Co. Ltd. The photoinitiator Irgacure 819 is supplied by Shanghai Guangyi Chemical Co. Ltd. Glycerol is purchased from Shanghai Lingfeng Chemical Reagent Co. Ltd. *N*-isopropylacrylamide (NIPAM) is obtained from Beijing Innochem Technology Co. Ltd. Ethoxylated (6) trimethylolpropane triacrylate (TMP6EOTA; 98%) is purchased from Curease Chemical. NdFeB particles (diameter, 150 μm) are obtained from Guangzhou Xinuode Co. Ltd. The ex vivo stomach is sourced from pigs purchased at a local market.

### Synthesis of hydrogel precursor

To prepare the hydrogel precursor, 0.5 ml of MAAc, 0.5 ml of AAc, and 2 g of NIPAM are uniformly mixed with 0.65 ml of TMP6EOTA as a crosslinker. To increase viscosity and maintain structural stability during curing, 0.3 g of PVP is added to the mixture. Subsequently, 1.5 ml of a mixed solvent of ethyl lactate and glycerol (mass ratio, 1:3) and 0.15 ml of photoinitiator solution (Irgacure 819/DMF, 5 wt %) are added. The mixture is stirred continuously at 1000 rpm until PVP completely dissolves. In the resulting hydrogel, AAc imparts pH-responsive deformation, while NIPAM contributes temperature sensitivity and mechanical reinforcement, preventing brittleness after short-term curing.

### Fabrication of the NdFeB pattern

A mask complementary to the desired NdFeB pattern is adhered to PI tape. The mask is fabricated through paper cutting, wax printing, or acrylic laser cutting. NdFeB particles are evenly spread across the masked PI surface, and excess particles are removed. The mask is then stripped using an appropriate solvent (e.g., anhydrous ethanol for paper or acrylic masks and ethyl acetate for wax masks). After air drying at room temperature, the patterned regions are magnetized using a magnetizer (Shenzhen Pengyongyuan Technology Co. Ltd.). The magnetized modules are then assembled on one side of PET tape according to the predesigned orientations, forming a complete magnetic skeleton pattern.

### Fabrication of the dual-programming soft robot

A printed robot design is placed flat on a glass slide (with the paper slightly narrower than the glass). PET double-sided adhesive tape is applied over the paper, extending slightly beyond its edges. The upper adhesive layer is then peeled off. PI tape carrying the patterned magnetic skeletons is affixed onto the PET tape according to the designed pattern and magnetization direction. Spacers of suitable thickness are placed at the tape edges, followed by pouring of the hydrogel precursor mixture. A second glass slide is placed on top and clamped tightly to ensure uniform thickness. The assembly is cured under UV light using a UV curing system (Mofang Co. Ltd.). After curing, the glass slides, paper, and PET tape are sequentially removed, leaving the NdFeB particles embedded within the hydrogel. The cured hydrogel is then cut according to the robot design, yielding a magnetically responsive soft robot with dual-programming deformation capability.

### In vivo gastric ulcer therapy in pig models

The protocols of anesthesia, surgery, and postsurgical care of the pigs all meet the requirements stated by the Animal Experimentation Ethics Committee and Licensing Office of the Shenzhen Advanced Medical Services Limited Company (ref. number AASE2601001P). Construction of the animal model involves meticulous preparation, including surgical ulcer induction, preoperative animal conditioning, and sterilization of surgical instruments. Healthy adult Bama pigs aged 6 months with a body weight of 20 to 30 kg are selected as experimental subjects. After procurement, the pigs undergo a 7-day quarantine to exclude potential pathogen infections, followed by acclimatization to minimize stress-related interference. According to the protocol, food is withheld for 24 hours and water for 12 hours before surgery to reduce the influence of gastric contents on intraoperative observation and robotic deployment. On the day of surgery, anesthesia is induced, and an ear marginal vein is cannulated for drug administration and fluid infusion. Following endotracheal intubation, anesthesia is maintained with isoflurane inhalation, and electrocardiographic monitoring is continuously performed to ensure physiological stability. A sterile electronic gastroscope is introduced orally to inspect the esophagus, cardia, and gastric cavity. Subsequently, the capsule endoscope loaded with the microrobot is transesophageally delivered into the stomach and initially positioned near the lesion. Under external magnetic actuation, the capsule orientation and position are manually adjusted to diagnose and localize the target site. Upon exposure to gastric acid, the microrobot is released from the capsule and gradually unfolds. Guided by an external magnetic field, the microrobot traverses the gastric folds and precisely anchors to the target gastric wall. Throughout the procedure, the capsule endoscope continuously records the microrobot deployment and anchoring process, while the electronic gastroscope synchronously captures robot release and intragastric locomotion, providing comprehensive visual data for subsequent therapeutic evaluation.

### Deformation simulation

We conduct deformation simulations using ABAQUS. For magnetically programmed deformation, a UEL subroutine is used to perform finite element analysis. The structure is divided into magnetized and nonmagnetized regions according to the patterned magnetic skeleton, and the corresponding material parameters are defined, including the remanent magnetization of the magnetized regions (*M* = 72.4 kA m^−1^) and the applied external magnetic field B. The material is assumed to be incompressible and modeled using a Neo-Hookean constitutive framework. To reduce potential asymmetry in the Cauchy stress caused by magnetic torque, eight-node linear hexahedral elements are used for meshing.

For simulating anisotropic programmed deformation, the robot is first segmented into rigid and flexible regions based on the geometry of the magnetic skeleton. The anisotropic curvature of the hydrogel containing the magnetic skeleton is experimentally measured. Combined with the hydrogel layer thickness, the anisotropic swelling coefficients are obtained and assigned to the material properties as swelling anisotropy parameters with defined orthogonal principal axes. Other material parameters are derived from mechanical testing using a universal testing machine. The mesh likewise uses eight-node linear hexahedral elements.

For coupled programming, the deformed nodal coordinates obtained from the anisotropic deformation simulation are exported and used as the initial configuration for the magnetically programmed deformation simulation. Because the change in initial coordinates does not affect other parameters or the homogenized calculations in the magnetic simulation, the final deformation results can be obtained without issue.

### Microneedle antibacterial assay

To verify the effectiveness of the microneedles as a local drug delivery interface, the hydrogel microneedles were immersed in amoxicillin (3 mg ml^−1^) and tetracycline (10 mg ml^−1^) solutions for 24 hours to ensure sufficient drug loading. *Escherichia coli* DH5α was then selected as a model bacterial strain, and a standardized bacterial lawn was prepared on LB agar plates. The drug-loaded microneedle robots were placed at the center of the petri dishes, allowing the microneedles to penetrate the surface of the agar/bacterial lawn. After 24 hours of coculture, clear inhibition zones were observed in both the amoxicillin- and tetracycline-loaded groups, whereas no obvious antibacterial effect was detected in the unloaded microneedle control group. These results demonstrate that the microneedle structure can mediate localized drug release and effectively inhibit bacterial growth.

### Cell viability assays

Human dermal fibroblasts (HDFs) were seeded into 96-well plates at a density of 1 × 10^4^ cells per well and allowed to adhere overnight. The culture medium was then replaced with either fresh complete medium (control group) or the DHSR leachate diluted 1:1 with complete medium (DHSR group). After 24 hours of incubation, cell viability was evaluated using the CCK-8 assay (Beyotime) according to the manufacturer’s protocol. Absorbance at 450 nm was measured using a microplate reader (BioTek ELx800).

### Live/dead staining

For live/dead fluorescence imaging, HDFs were seeded into 24-well plates at a density of 5 × 10^4^ cells per well and treated as described above. After 24 hours, the cells were stained with calcein-AM (2 μM) and propidium iodide (4 μM) (Live/Dead Viability/Cytotoxicity Kit, Solarbio) for 15 min at 37°C. Fluorescence images were captured using an inverted fluorescence microscope (Olympus CKX41). Live cells appeared green (calcein-AM), and dead cells appeared red (propidium iodide). All experiments were performed in triplicate.
