# Supplementary material for: Adaptive dual-programming magnetic soft robot with lifelike behavior
Source: Sci Adv. 2026 Nov 6;12(45):eaeh1001. doi: 10.1126/sciadv.aeh1001 (PMC13742183; doi:10.1126/sciadv.aeh1001)
Supplement: Supplementary file 1 [file sciadv.aeh1001_sm.pdf]

Science Advances
AAAS

Supplementary Materials for

Adaptive dual-programming magnetic soft robot with lifelike behavior

Yanchen Ye *et al.*

Corresponding author: Li Zhang, lizhang@mae.cuhk.edu.hk; Yue Dong, dongyue@hit.edu.cn;
Bing Li, libing.sgs@hit.edu.cn

Sci. Adv. **12**, eaeh1001 (2026)
DOI: 10.1126/sciadv.aeh1001

The PDF file includes:

Figs. S1 to S33
Tables S1 to S5
Legends for movies S1 to S8
Legend for data S1

Other Supplementary Material for this manuscript includes the following:

Movies S1 to S8
Data S1

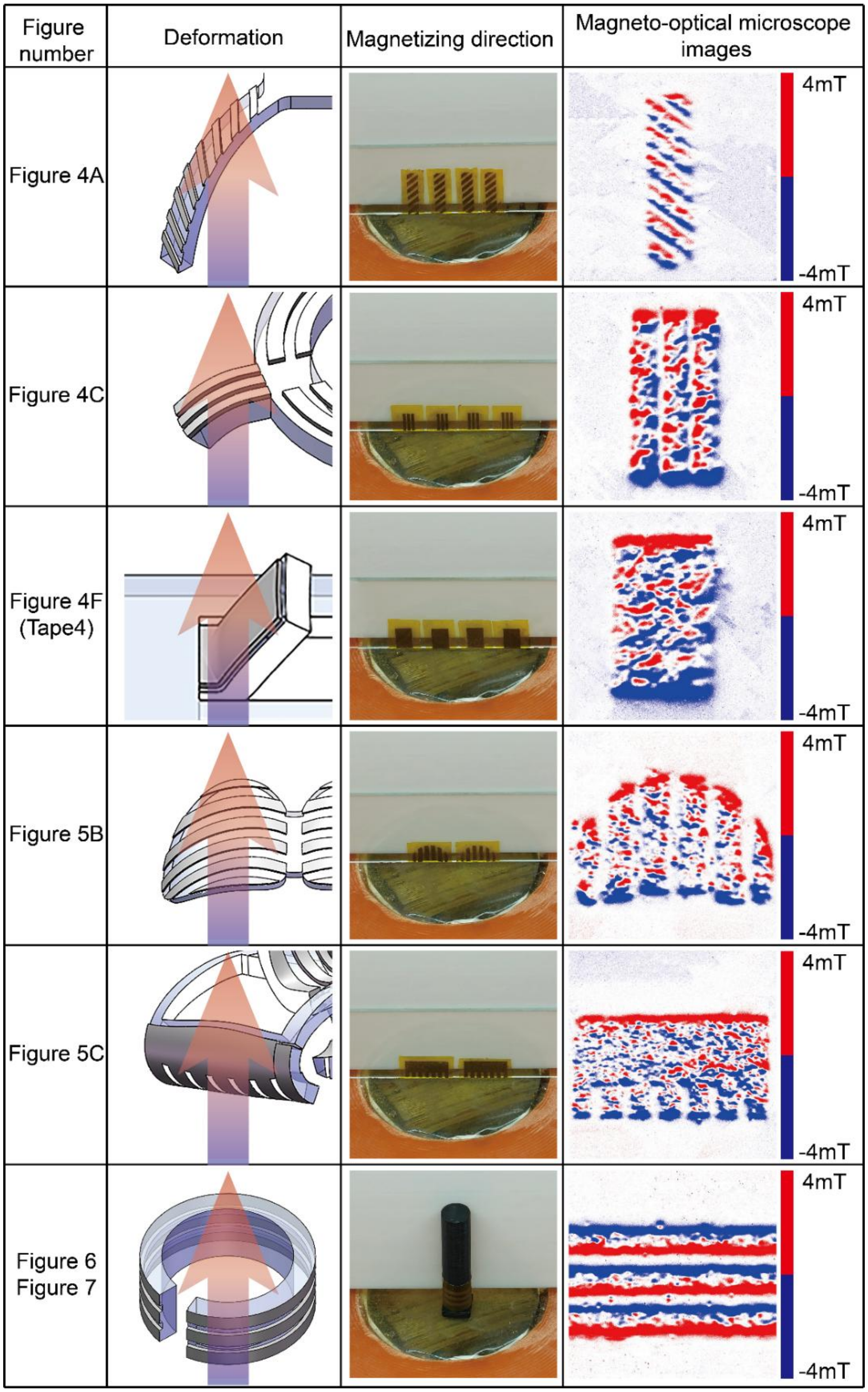

Fig. S1. Magnetization of NdFeB patterns. The table shows representative robots' magnetic deformation, magnetization directions of magnetic particles, and magneto-optical images after magnetization. All magnetic modules in the robots are magnetized using a template-assisted method.

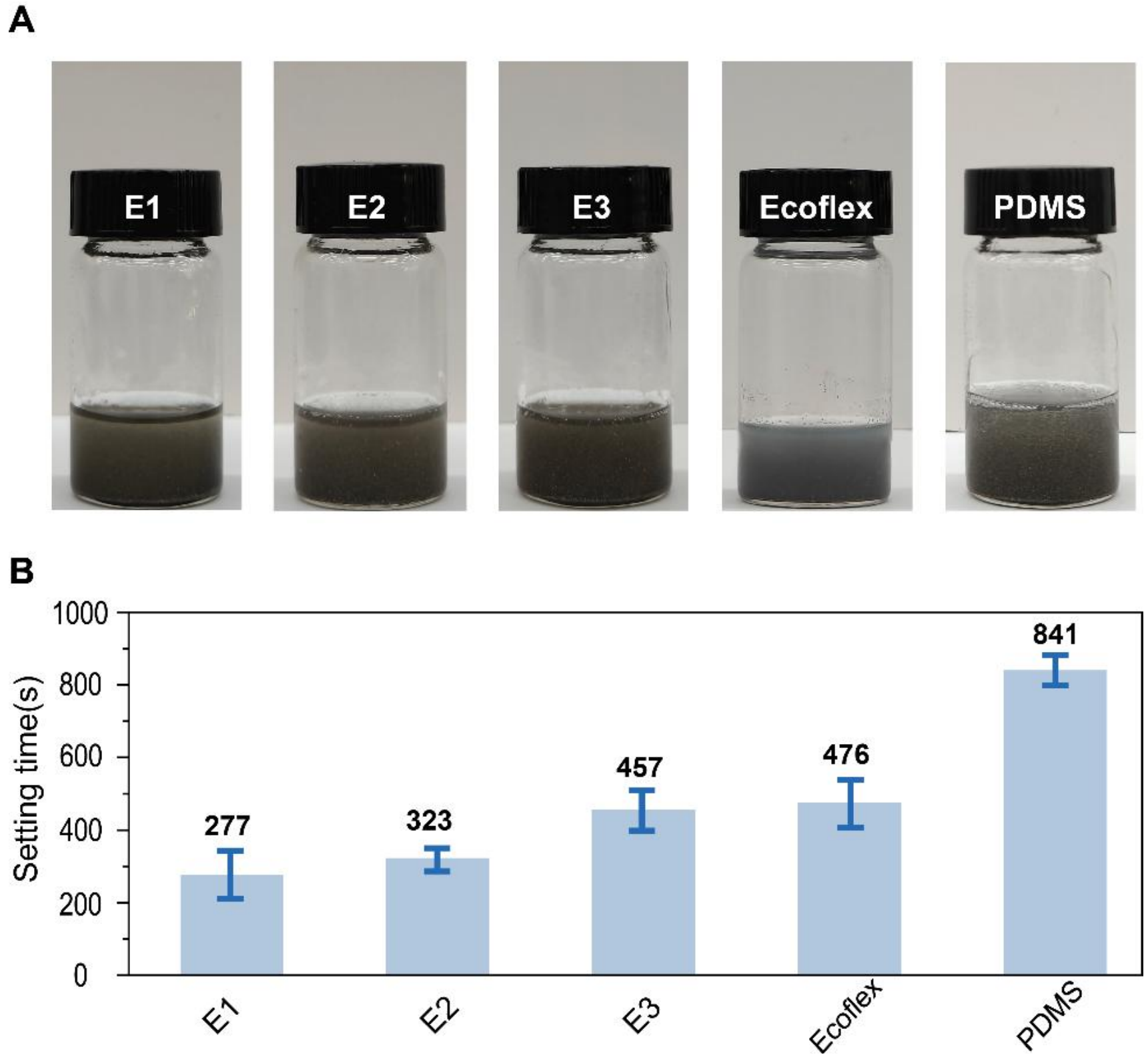

Fig. S2. Sedimentation rate of magnetic particles in different flexible matrix precursors. (A) NdFeB magnetic particles are dispersed in four types of flexible matrix precursors: two commonly utilized soft matrix precursors (2 wt% sodium alginate, E1; Ecoflex; polydimethylsiloxane, PDMS) and two hydrogel precursors developed in this work (temperature-responsive hydrogel, E2; pH-responsive hydrogel, E3). The NdFeB magnetic particle content is 20% in all cases; (B) All systems exhibit distinct sedimentation gradients within approximately 1 minute of dispersion. The two custom-developed hydrogel precursors, the time required to form stable sedimentation gradients is determined to be 323 seconds (temperature-responsive hydrogel, E2) and 457 seconds (pH-responsive hydrogel, E3), respectively.

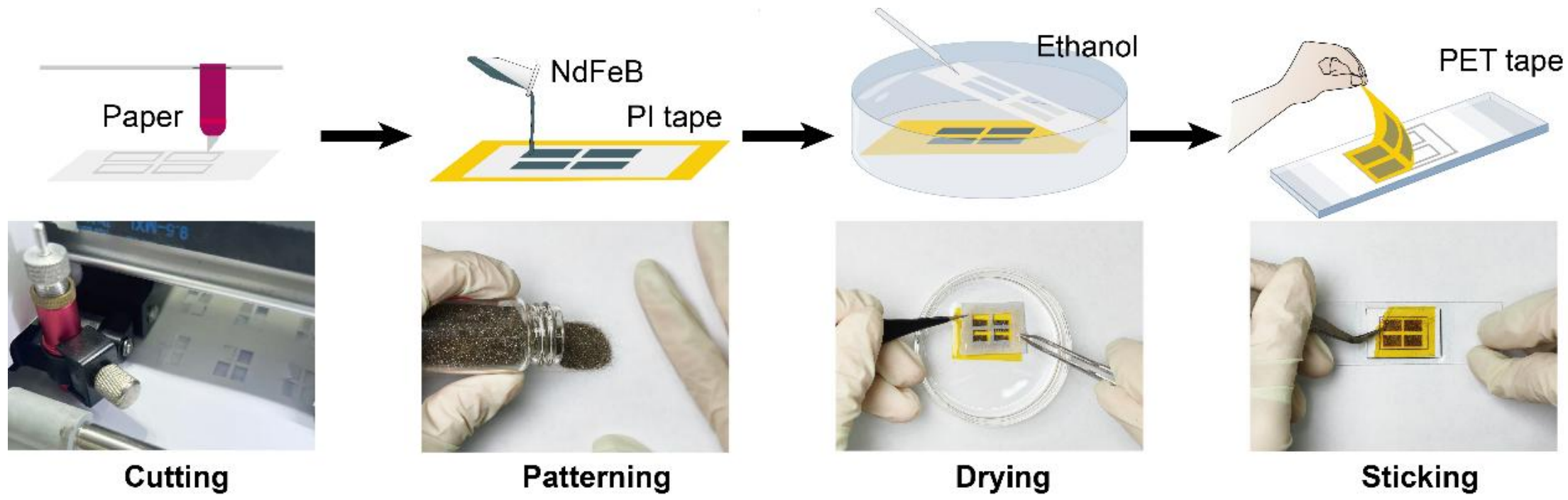

Fig. S3. Fabrication and transfer of NdFeB patterns. Using a paper template, NdFeB patterns are cut with a cutting machine, placed on polyimide (PI) tape, and filled with NdFeB particles. After soaking in anhydrous ethanol, the paper is readily removed. Once dry, the patterned PI tape is magnetized and transferred to polyethylene terephthalate (PET) tape, leveraging the adhesion difference between the tapes.

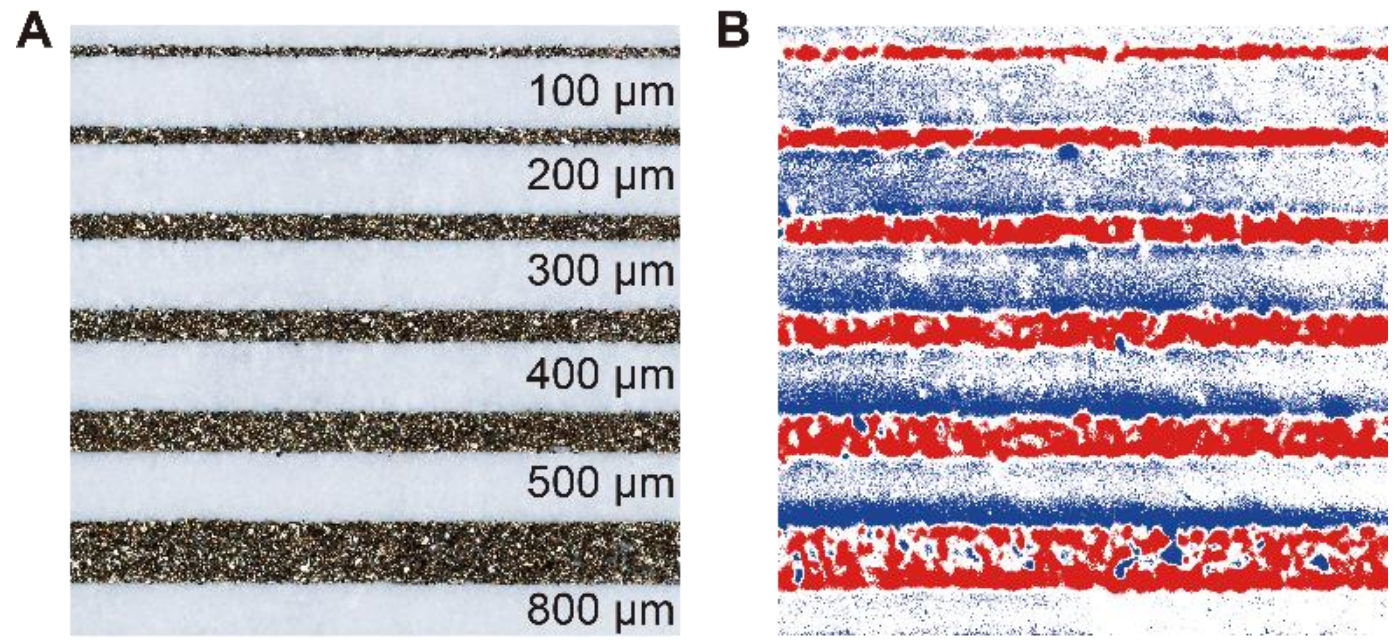

Fig. S4. The fabrication accuracy of NdFeB stripes. (A) The super-resolution optical image and **(B)** magneto-optical microscope image of NdFeB stripes with different widths.

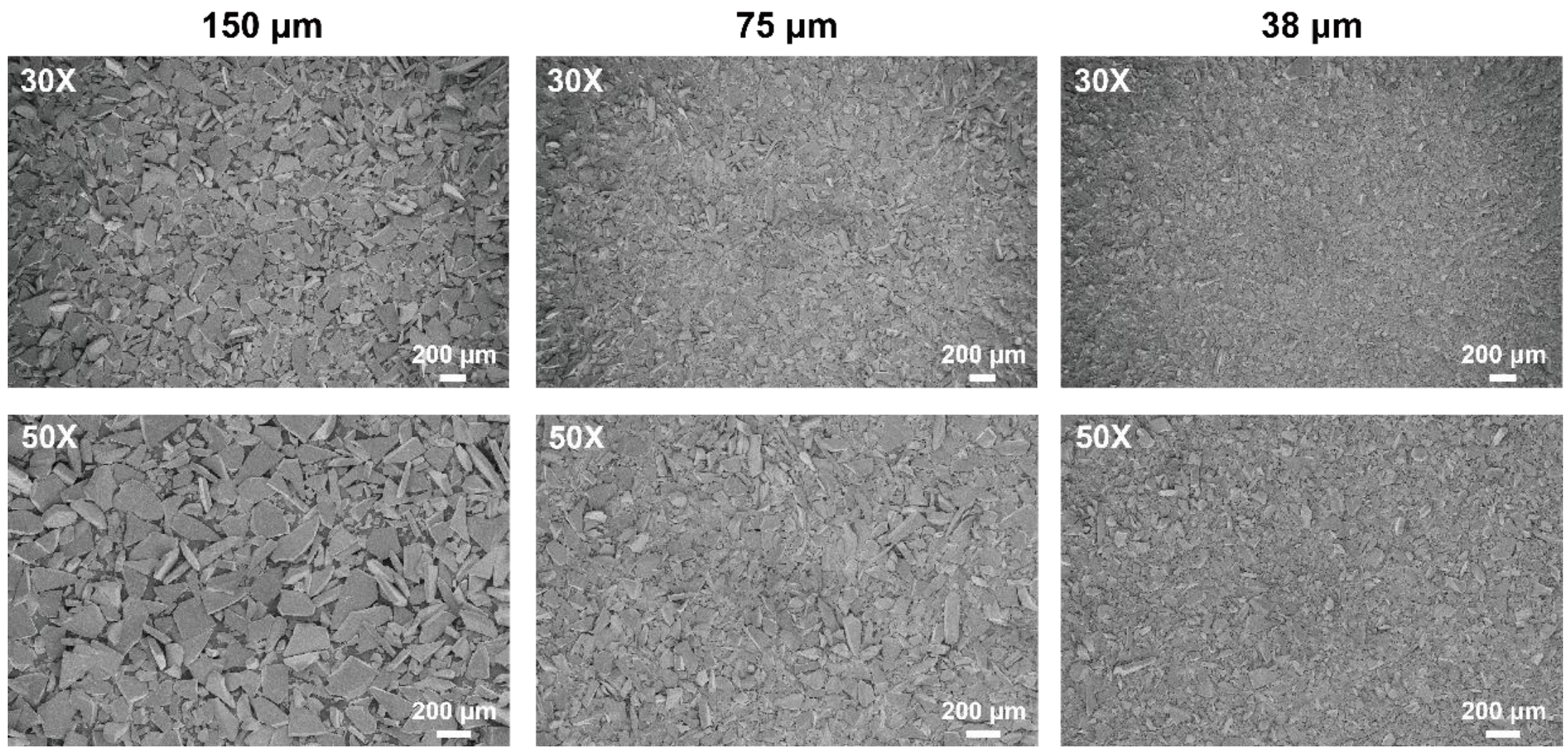

Fig. S5. The SEM images of NdFeB microparticles with different sizes (150 μm, 75 μm, and 38 μm) in the double-sided tapes.

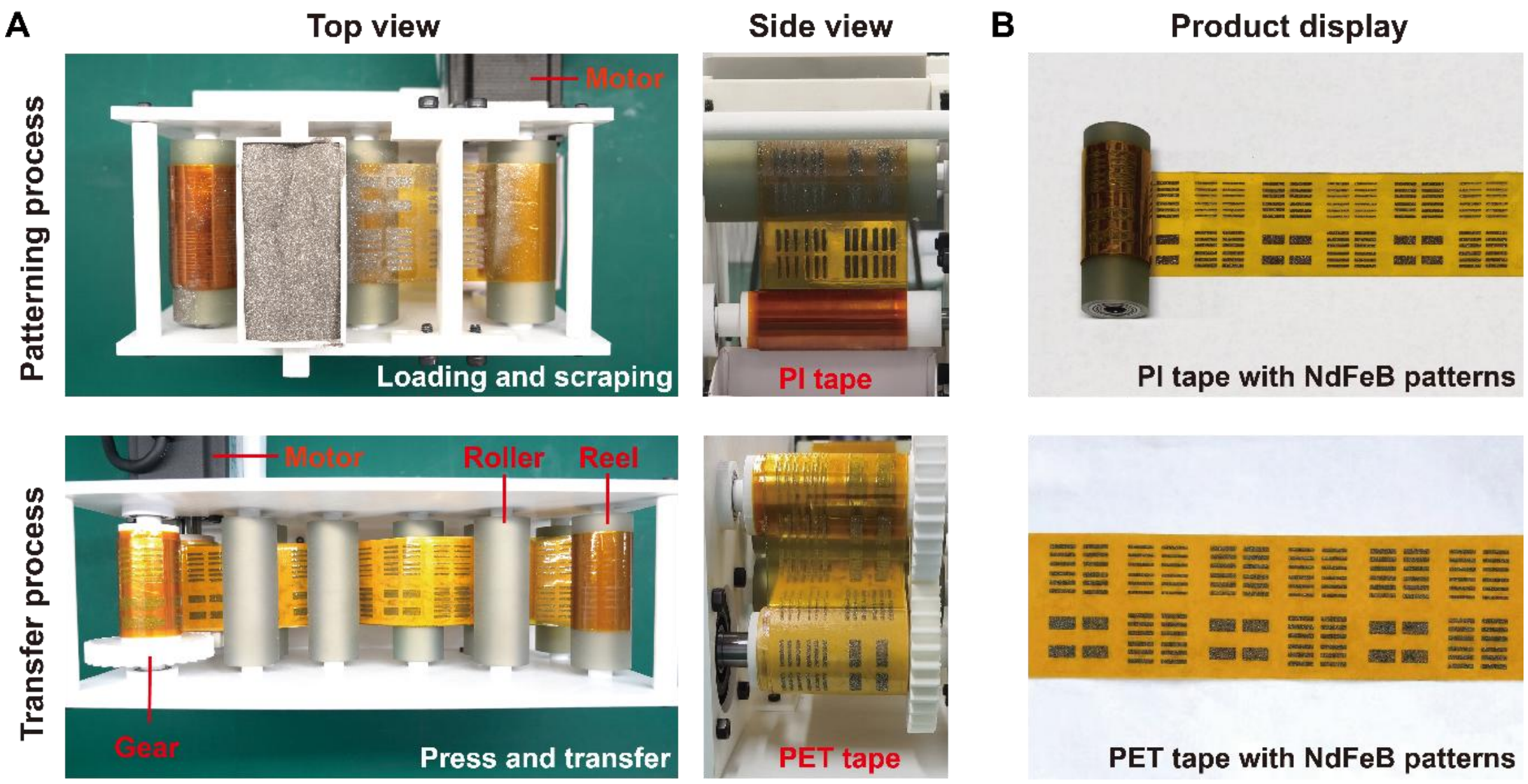

Fig. S6. The automated roll-to-roll fabrication system of the DHSR. (A) The assembly process with roll-to-roll technology. **(B)** Magnetized NdFeB patterns fixed on the reel.

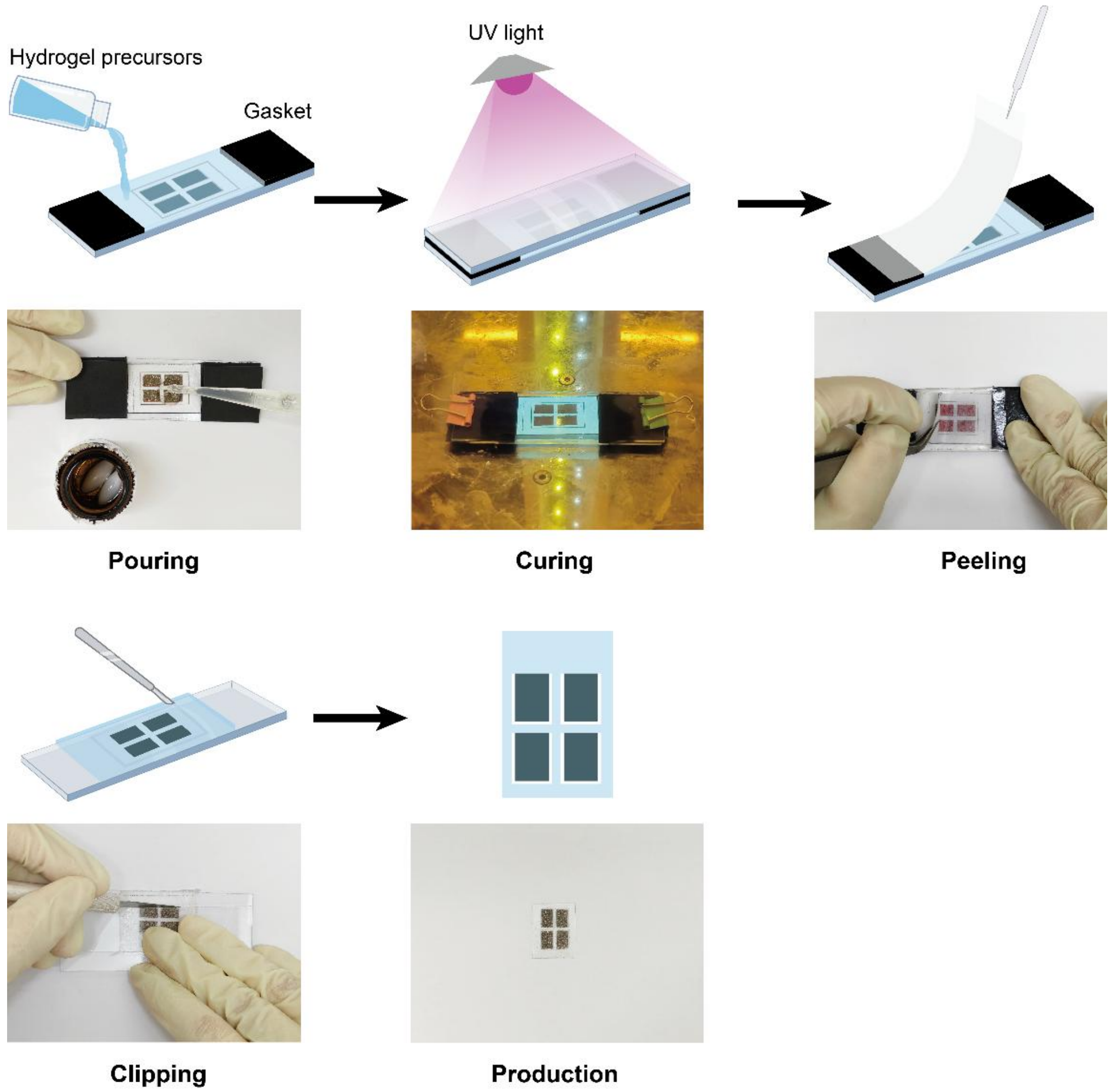

Fig. S7. Fabrication process of hydrogel-based robots. Taking the robot shown in Figure 3C as an example: after attaching the magnetic pattern, gaskets matching the robot's thickness are placed at both ends of the glass slide. The hydrogel precursor is poured over the pattern, covered with another glass slide, and clamped. After UV curing for a set time, the clamp and top glass are removed, and the backing PET tape and pattern paper are peeled off. Finally, the robot is cut into shape as designed.

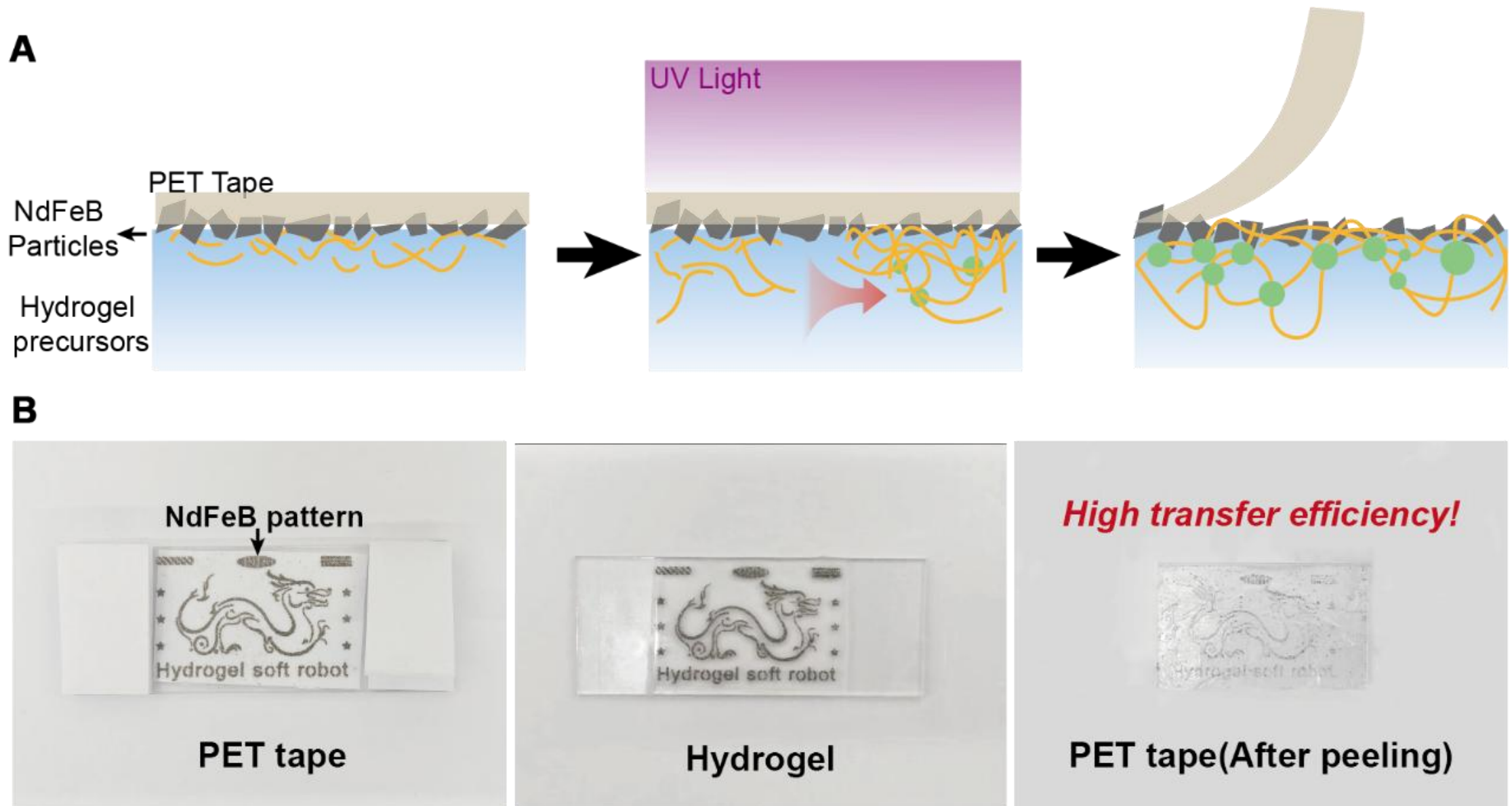

Fig. S8. Mechanism of efficient NdFeB removal from PET tape. (A) Under UV light, the hydrogel forms a crosslinked network that tightly encapsulates NdFeB particles, generating a force stronger than the tape's adhesion, enabling efficient removal. **(B)** Images show the NdFeB pattern on PET before casting, on the hydrogel after removal, and on PET after removal, confirming high transfer efficiency.

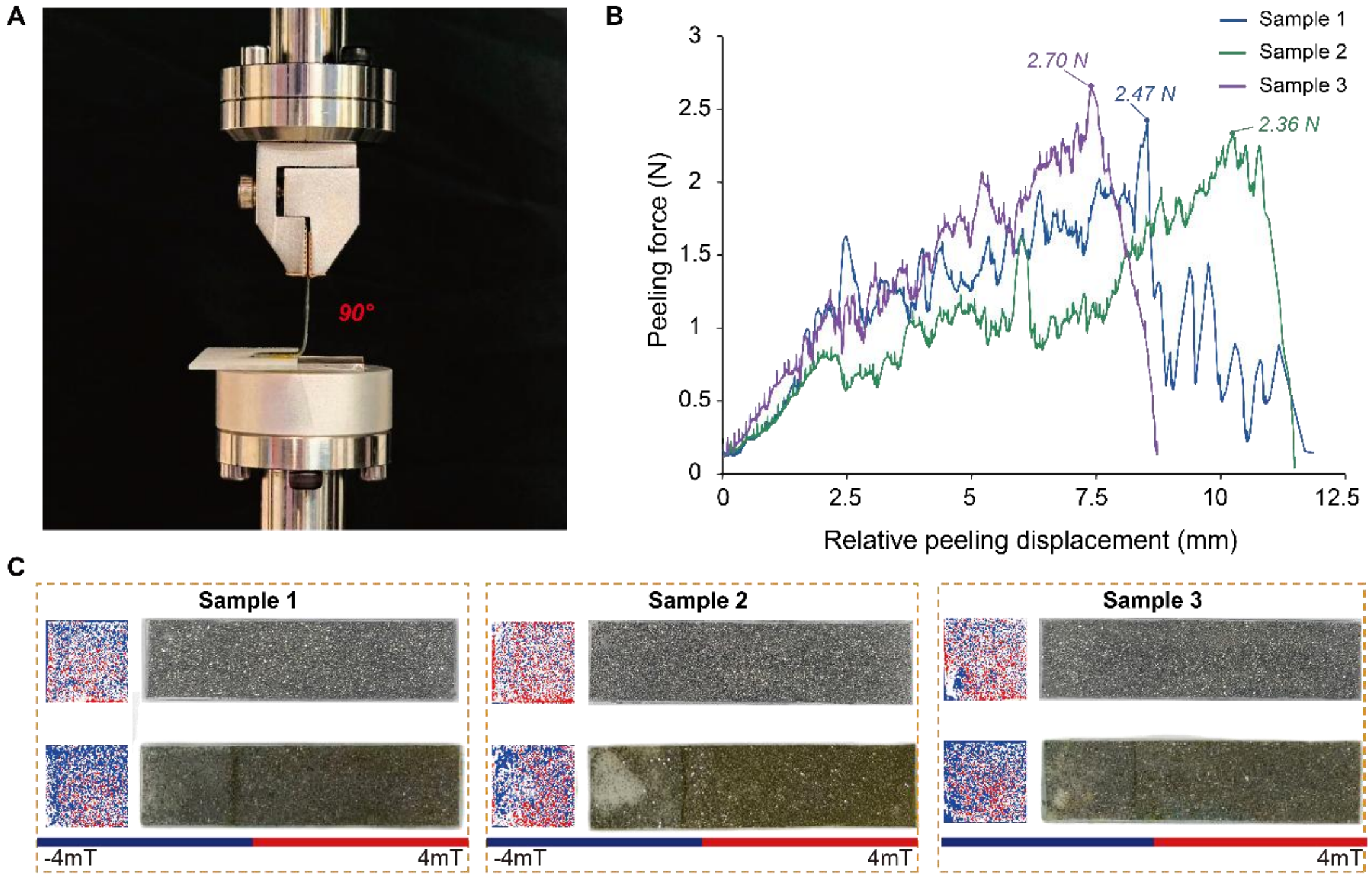

Fig. S9. The adhesion stability experiment of the hydrogel-NdFeB interface. (A) Experimental image of 90° peeling test for interfacial adhesion force between the hydrogel and the NdFeB pattern. **(B)** The peeling force test of three same DHSR samples. **(C)** The optical and magneto-optical microscopy images of three same DHSR samples before and after peeling.

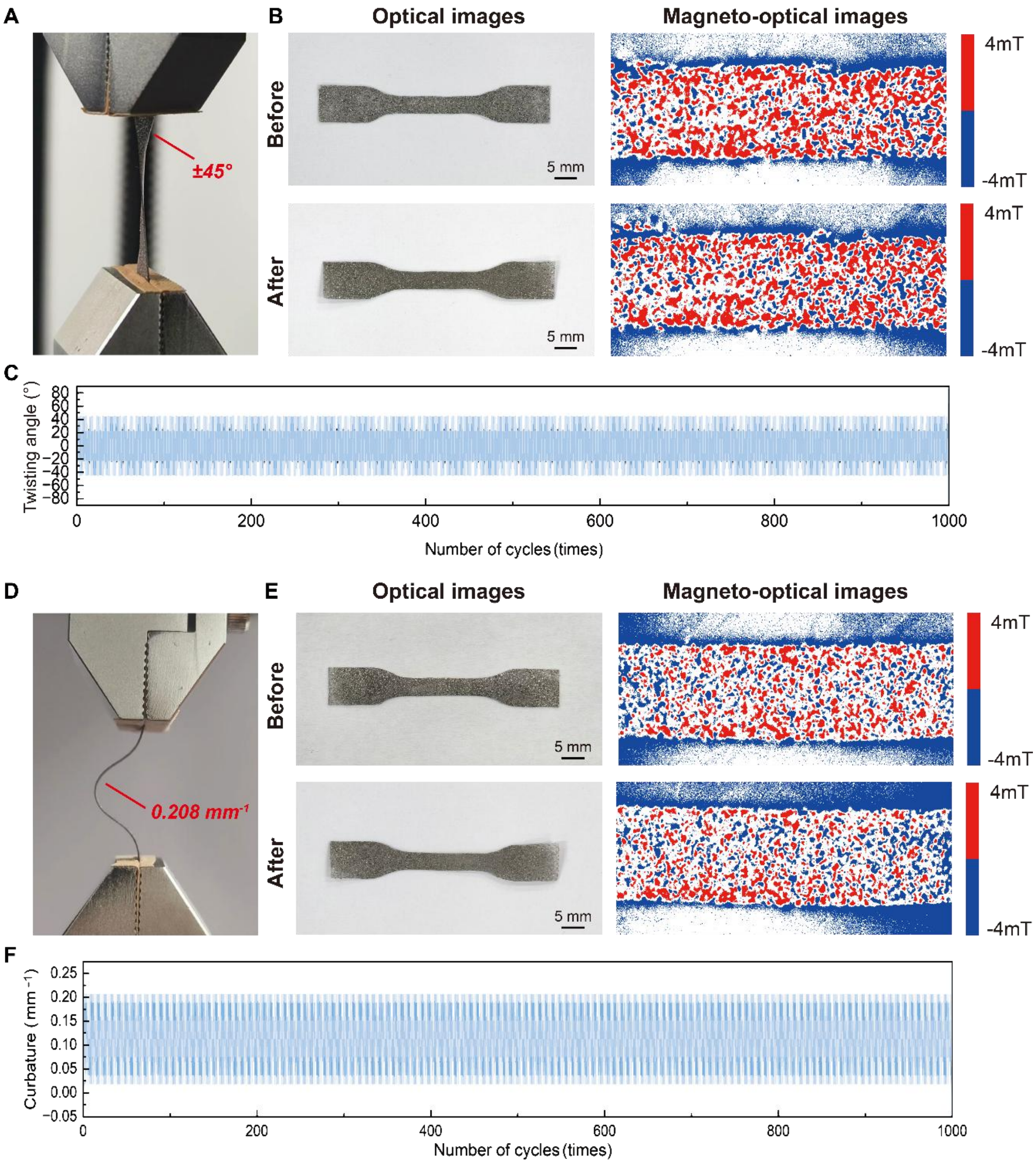

Fig. S10. Stability Experiment under repeated mechanical deformation. **(A)** Experimental image of ±45° twisting fatigue test. **(B)** The optical images and magneto-optical microscopy images of DHSR after 1000 passive twisting cycles. **(C)** The cycling stability of twisting angle for DHSR after 1000 passive twisting cycles. **(D)** Experimental image of bending tests. **(E)** The optical images and magneto-optical microscopy images of DHSR after 1000 passive bending tests. **(F)** The cycling stability of bending curvature for DHSR after 1000 passive twisting cycles.

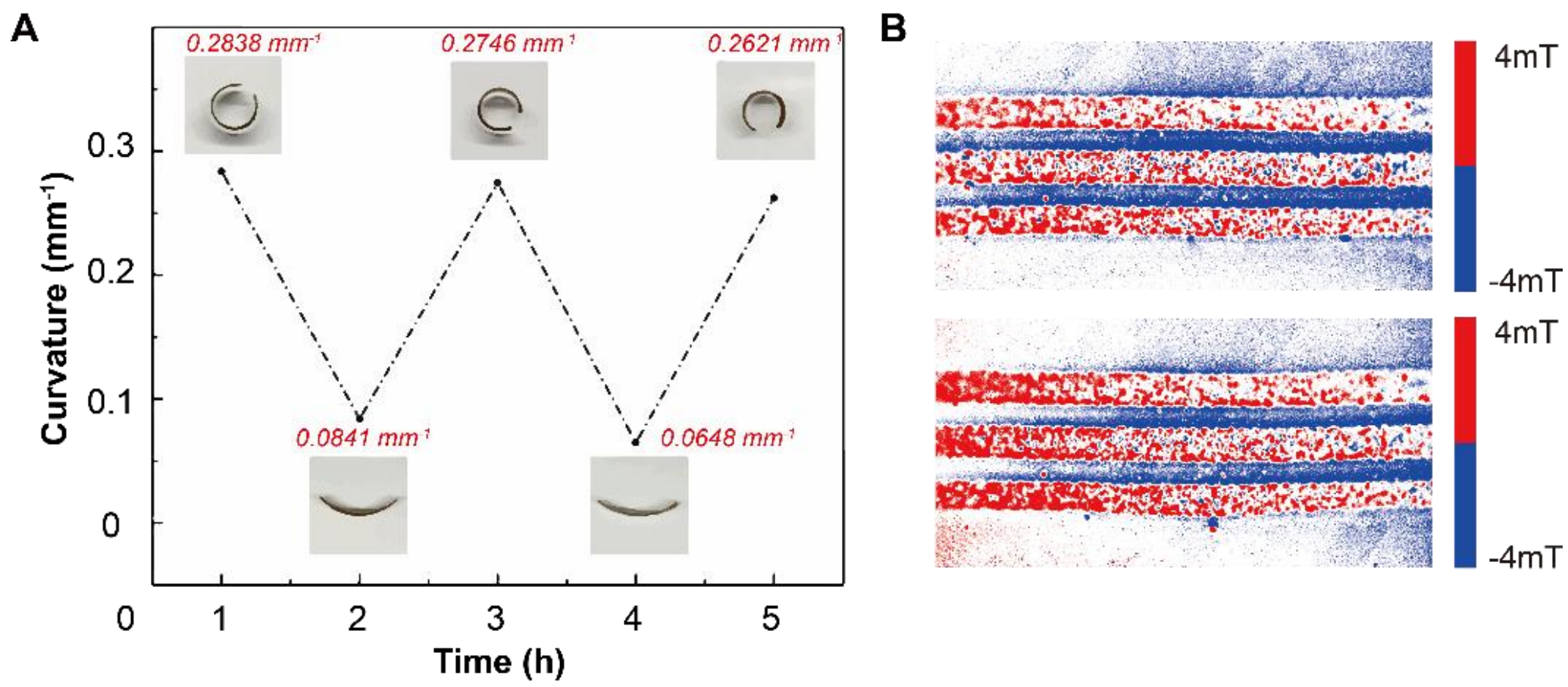

Fig. S11. The repeated swelling deformation experiment of the DHSR. (A) The shrinkage and swelling cycles test of DHSR. **(B)** The magneto-optical images of cyclic DHSR when being returned to the unfolded state.

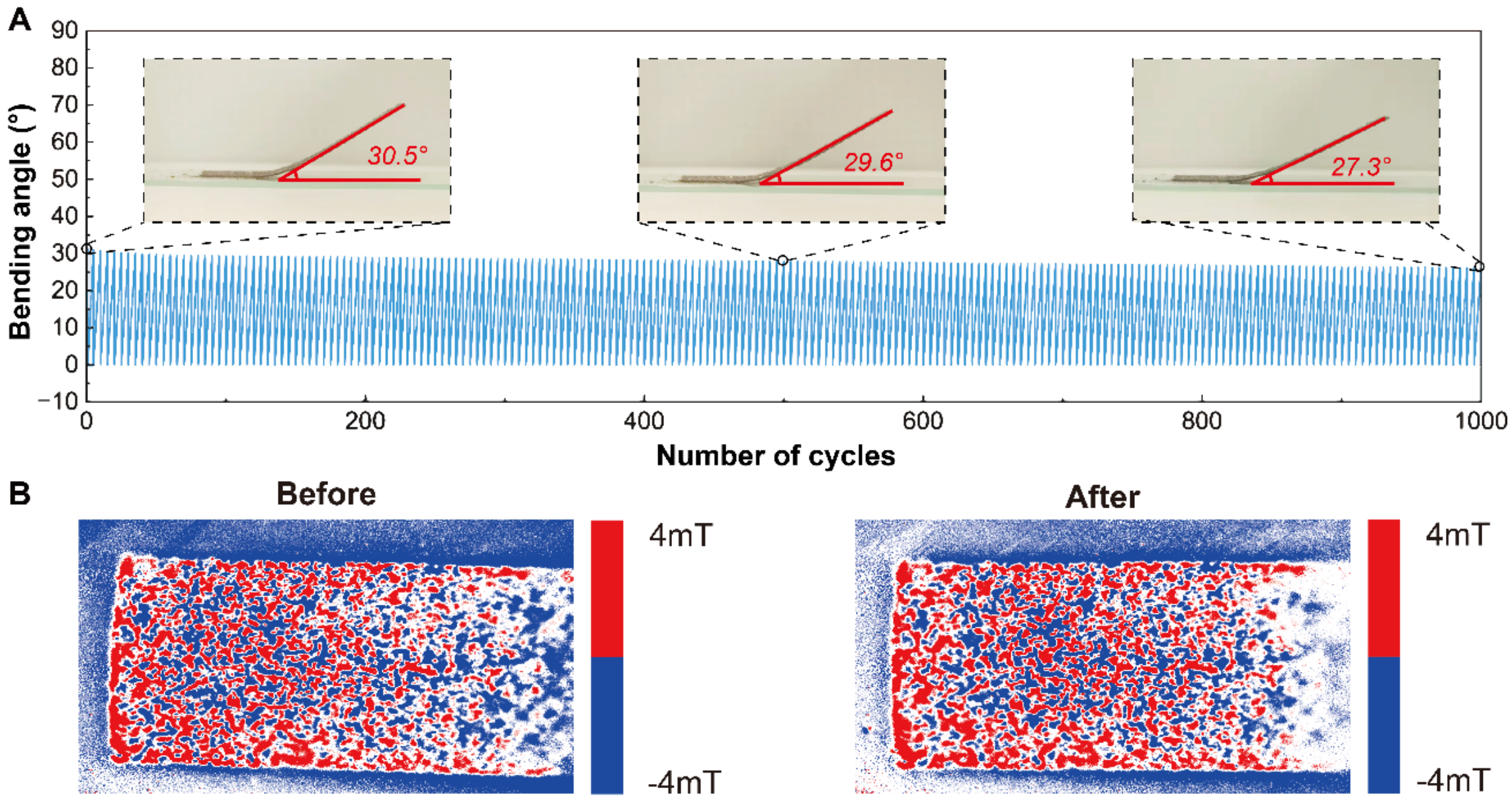

Fig. S12. Stability experiment of the DHSR under repeated magnetic actuation. (A) The stability of the DHSR under 1000 cyclic magnetic actuation. **(B)** The magneto-optical images of the DHSR before and after 1000 cyclic magnetic actuation.

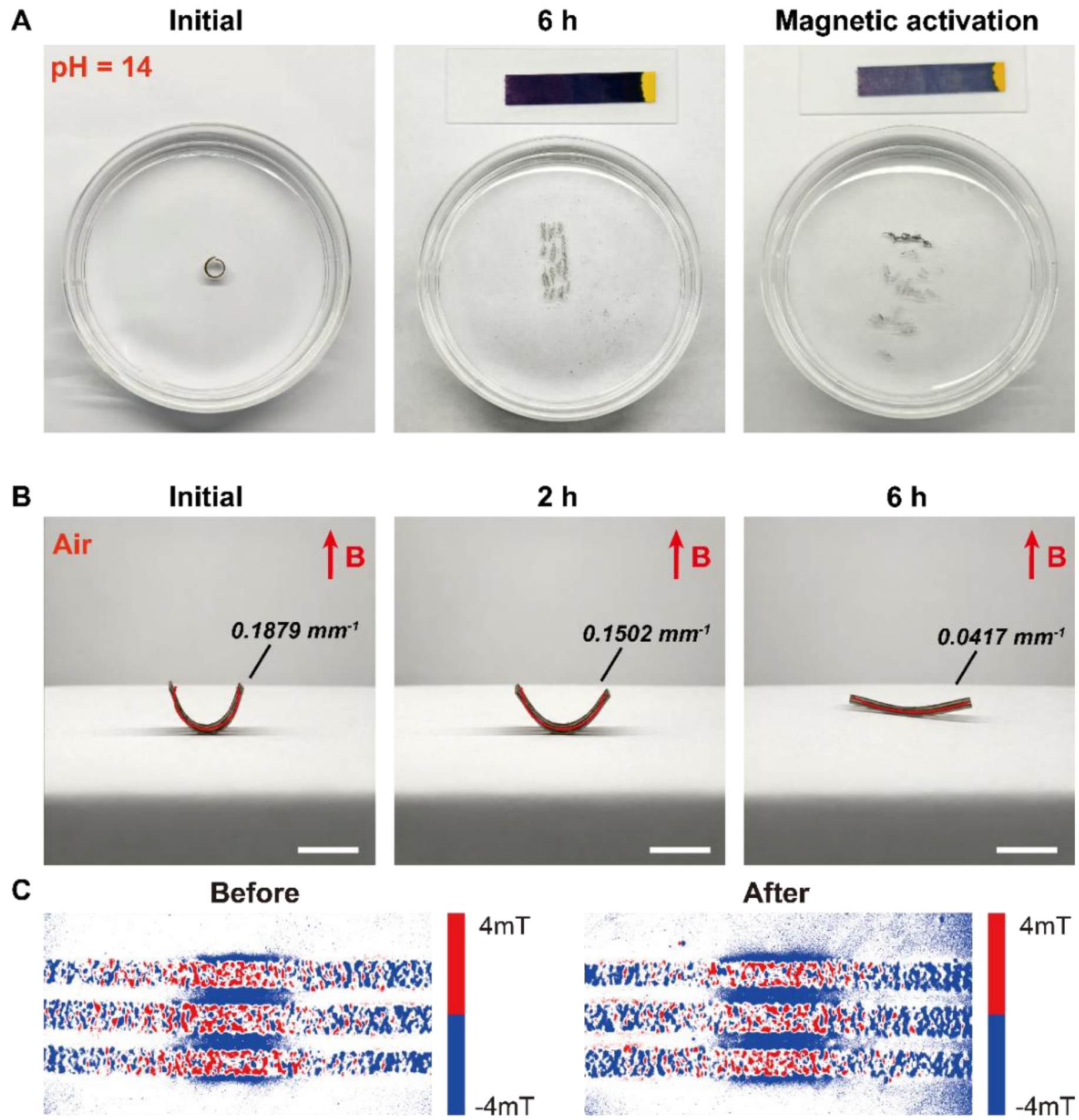

Fig. S13. The failure of the robot when exposed to (A) alkaline conditions and (B) air. (C) The magneto-optical images before and after 6h air exposure. Scale bar: 10 mm.

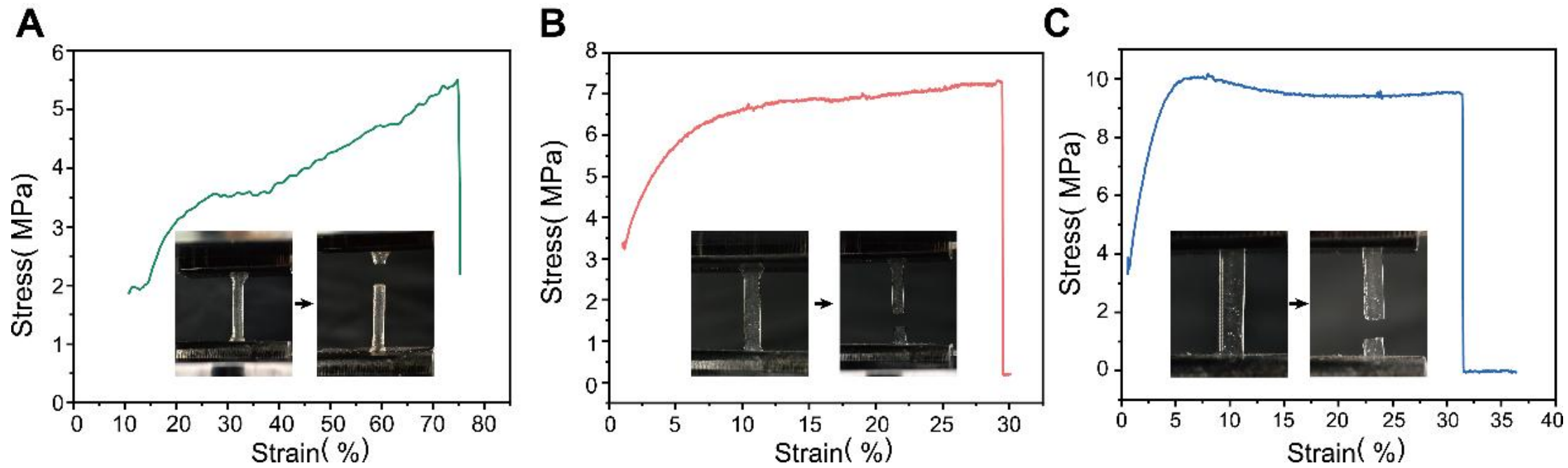

Fig. S14. Effect of crosslinking time on hydrogel mechanical properties. Stress-strain curves of hydrogels cured for (A) 30s, (B) 45s, and (C) 60s. As crosslinking time increases, tensile strength improves from 5.625 MPa (30s) to 10.17 MPa (60s), indicating enhanced mechanical properties.

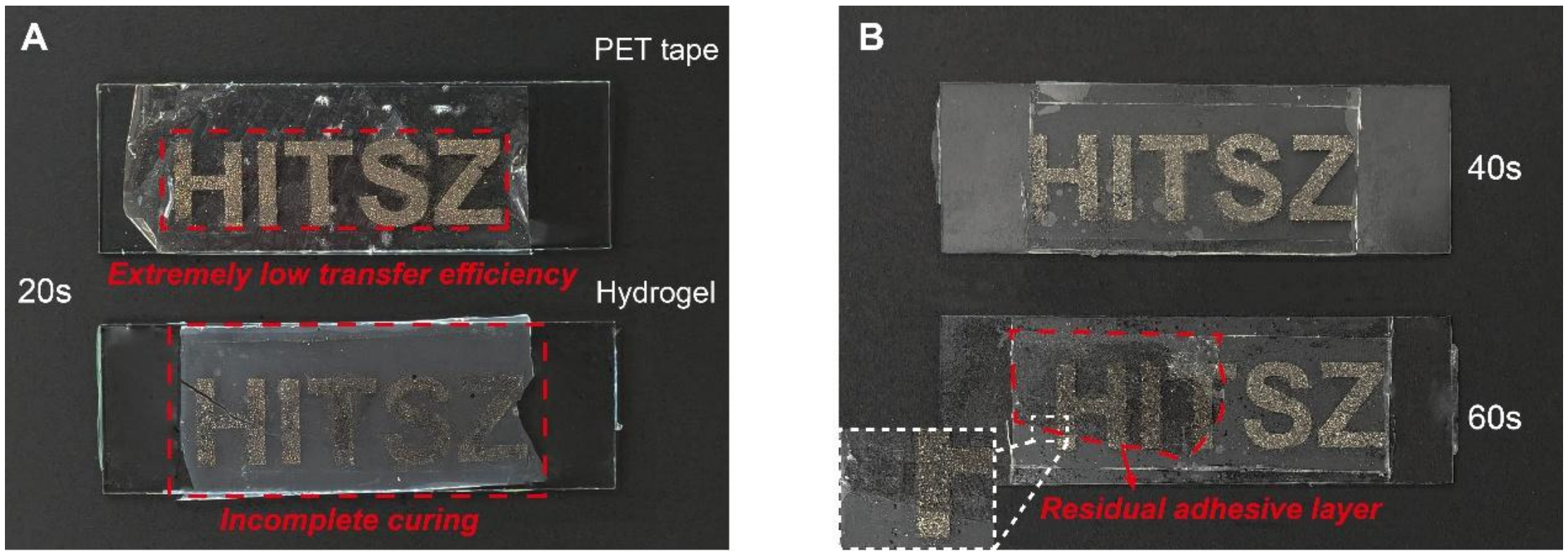

Fig. S15. Effect of crosslinking time on PET tape removal and NdFeB transfer efficiency. (A) Hydrogel cured for 20s is soft and fragile due to incomplete crosslinking, leading to poor NdFeB encapsulation and low transfer efficiency. **(B)** After 40s curing, the hydrogel shows complete pattern transfer with clean tape removal. After 60s curing, over-crosslinking causes strong adhesion between the hydrogel and tape, making removal difficult.

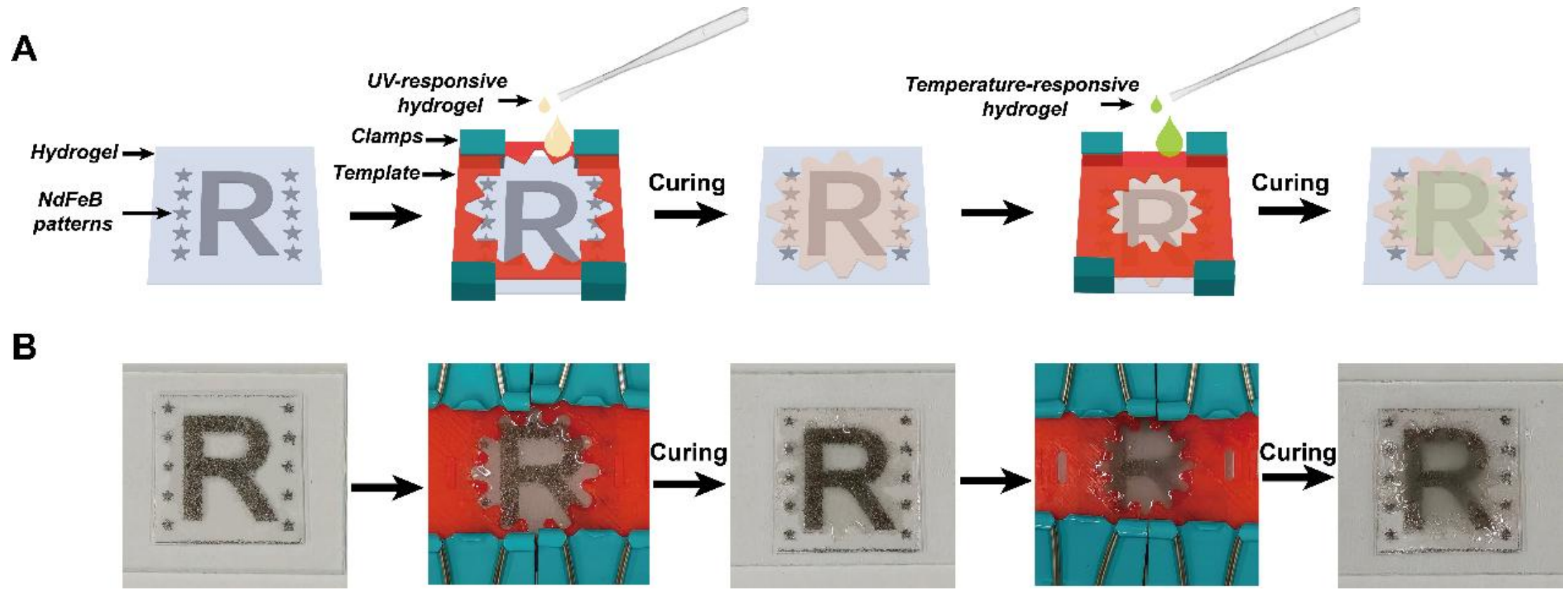

Fig. S16. Fabrication of multi-responsive multilayer hydrogel. (A) Schematic of the process. **(B)** Photo of the sample. The bottom hydrogel-NdFeB layer is prepared as in Fig. S3. A 3D-printed template is clamped above, responsive hydrogel is cast and cured. After removing the template, the responsive hydrogel layer remains. Repeating this forms a multilayer structure with multiple responses.

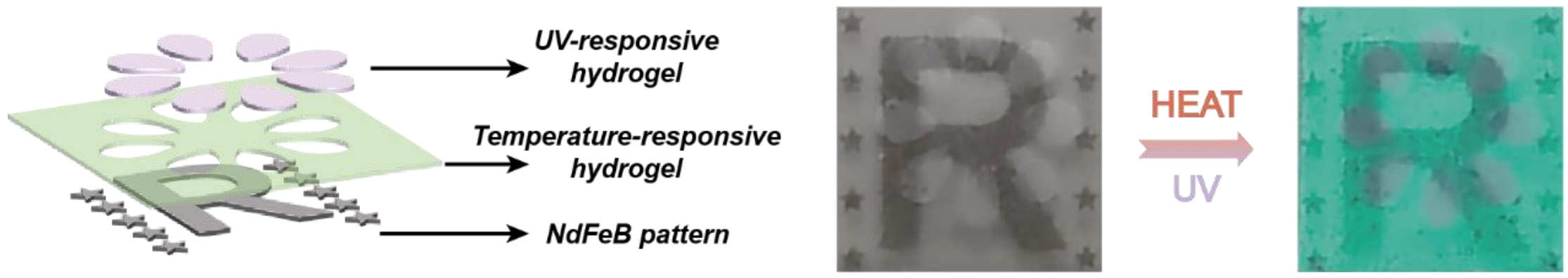

Fig. S17. Single-layer multi-responsive hydrogel-NdFeB structure. Using a template-assisted method, a single-layer hydrogel with multiple responses can be fabricated. Under UV- and temperature-stimuli, the hydrogel exhibits distinct color changes.

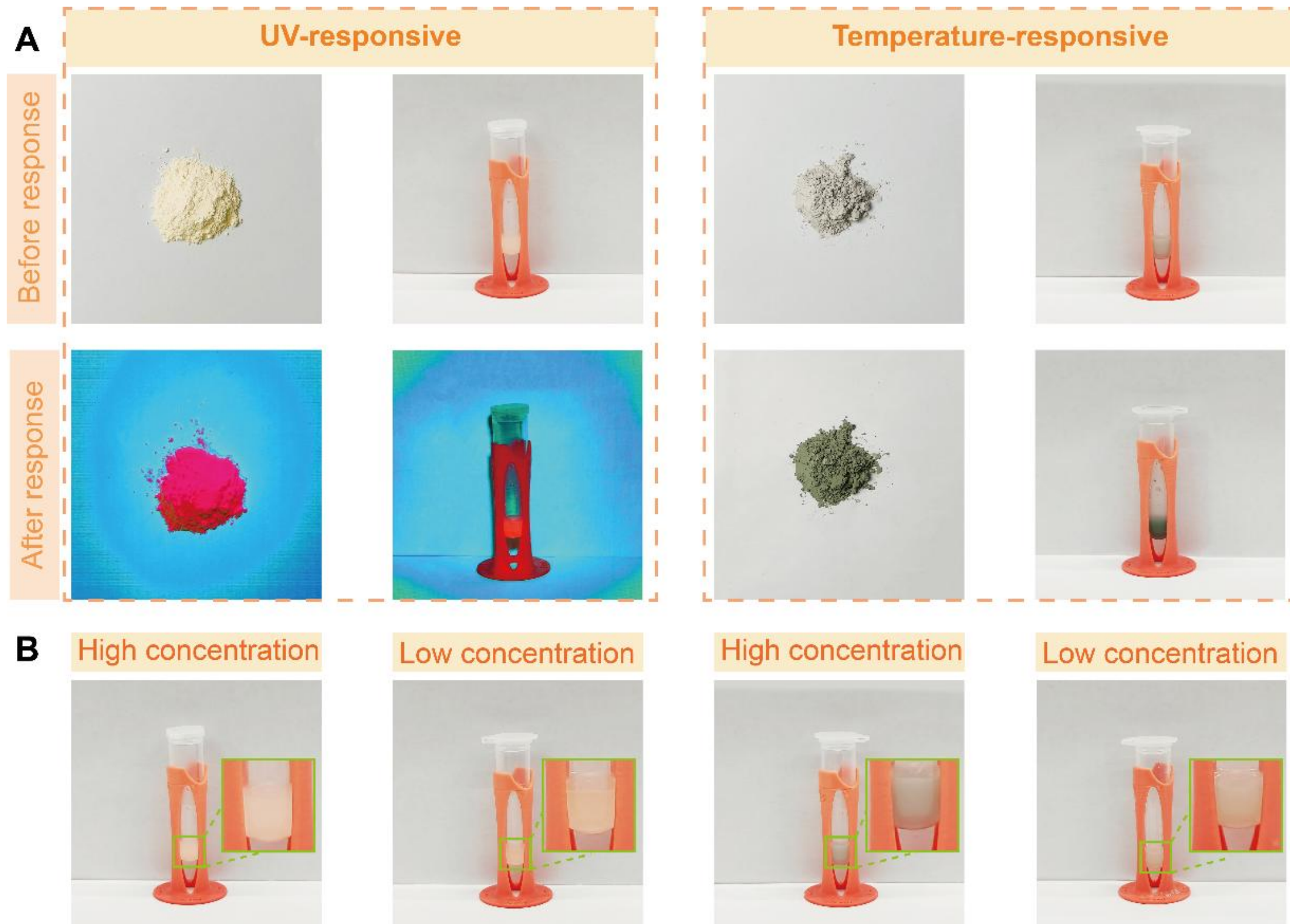

Fig. S18. Stimuli-responsive powders and hydrogels. (A) UV- and temperature-responsive (40 °C) powders and corresponding composite hydrogels. Incorporating the powders into the hydrogel precursor imparts responsiveness to the respective stimuli. **(B)** Reducing the powder content increases hydrogel transparency, enabling clearer visualization of the NdFeB patterns.

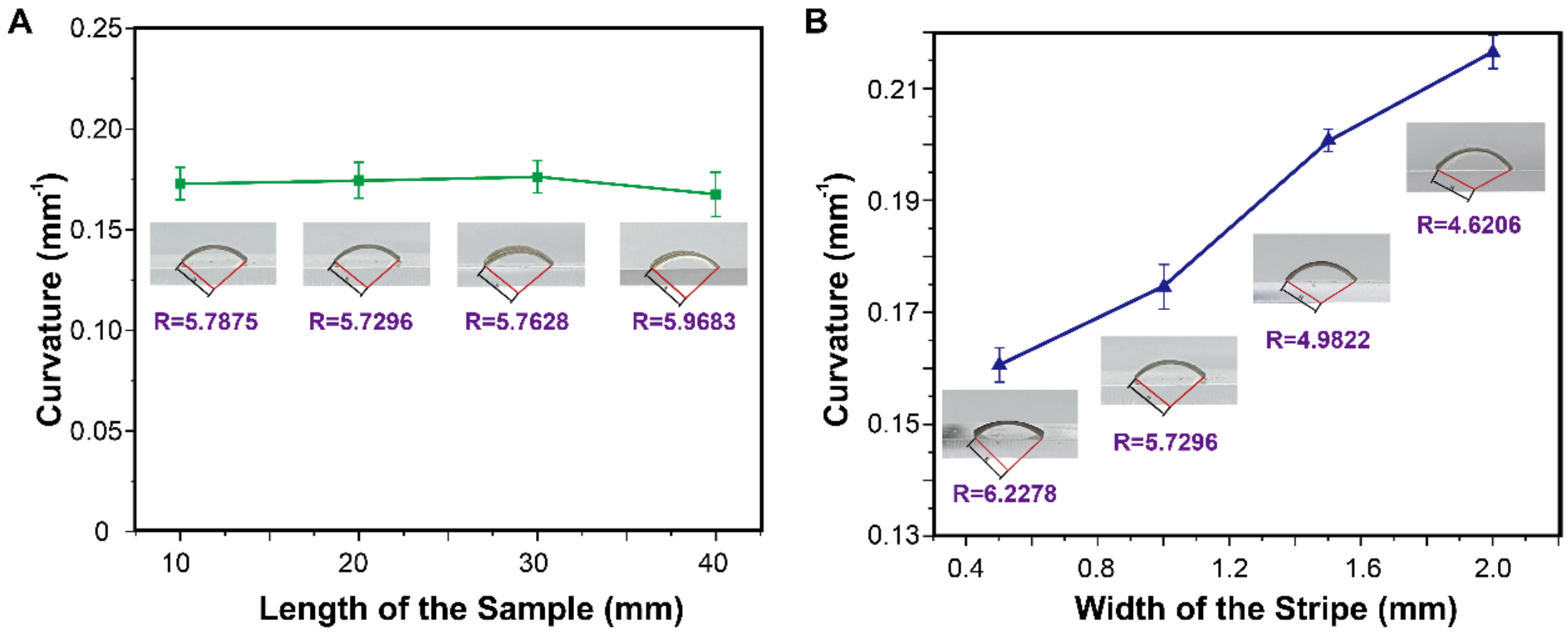

Fig. S19. Influence of magnetic stripe width and hydrogel strip length on anisotropic deformation of hydrogel. (A) The length of the hydrogel strip has minimal effect on anisotropic deformation. **(B)** For transverse stripes, wider magnetic stripes lead to more obvious anisotropic deformation and higher bending curvature.

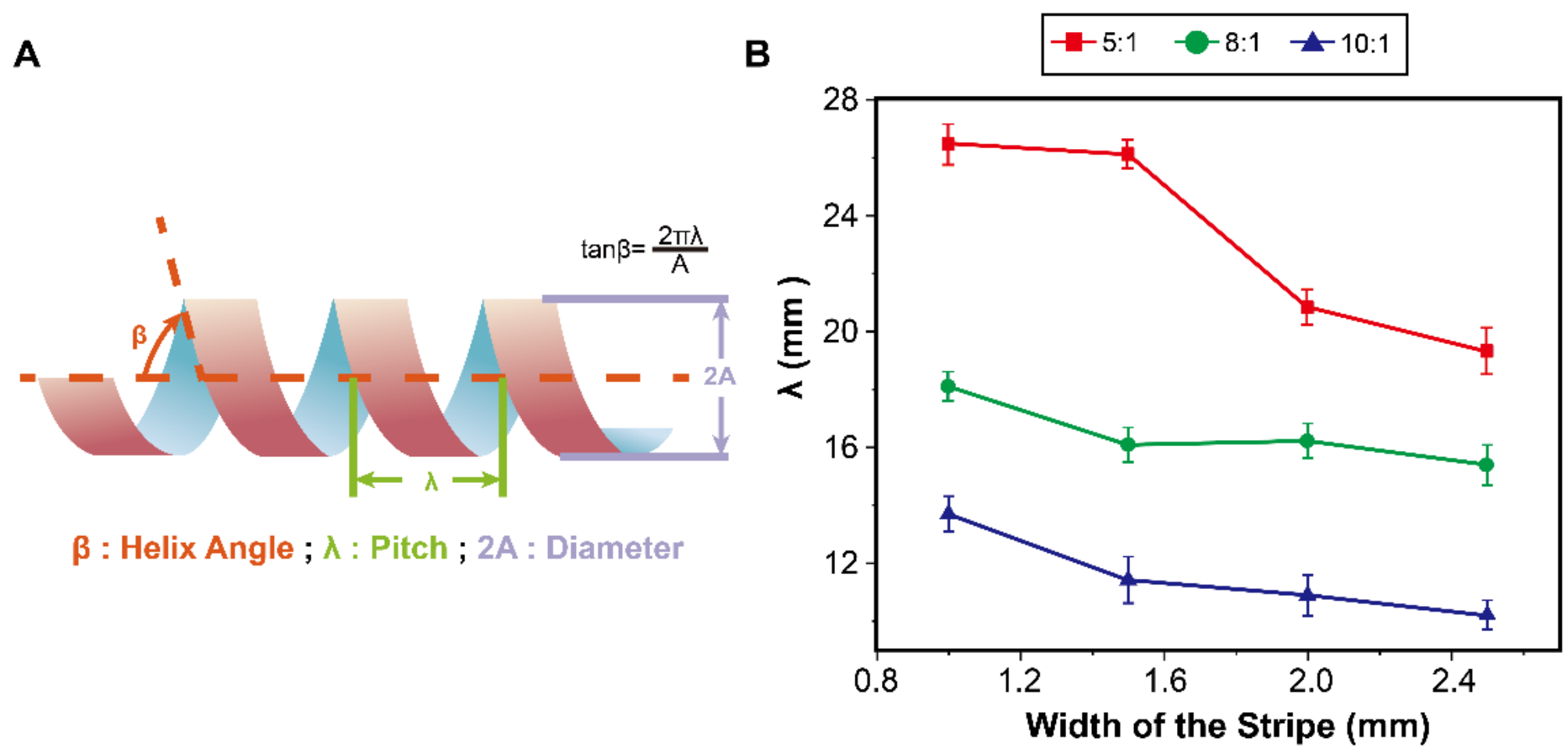

Fig. S20. Influence of magnetic oblique stripe width and aspect ratio of the hydrogel strip on helical deformation of the hydrogel. (A) Method for calculating helical pitch. **(B)** Wider magnetic oblique stripes result in more obvious anisotropic deformation. For a fixed length, increasing the aspect ratio also enhances anisotropic deformation. Values are means, and error bars indicate standard deviation (n = 3 independent samples).

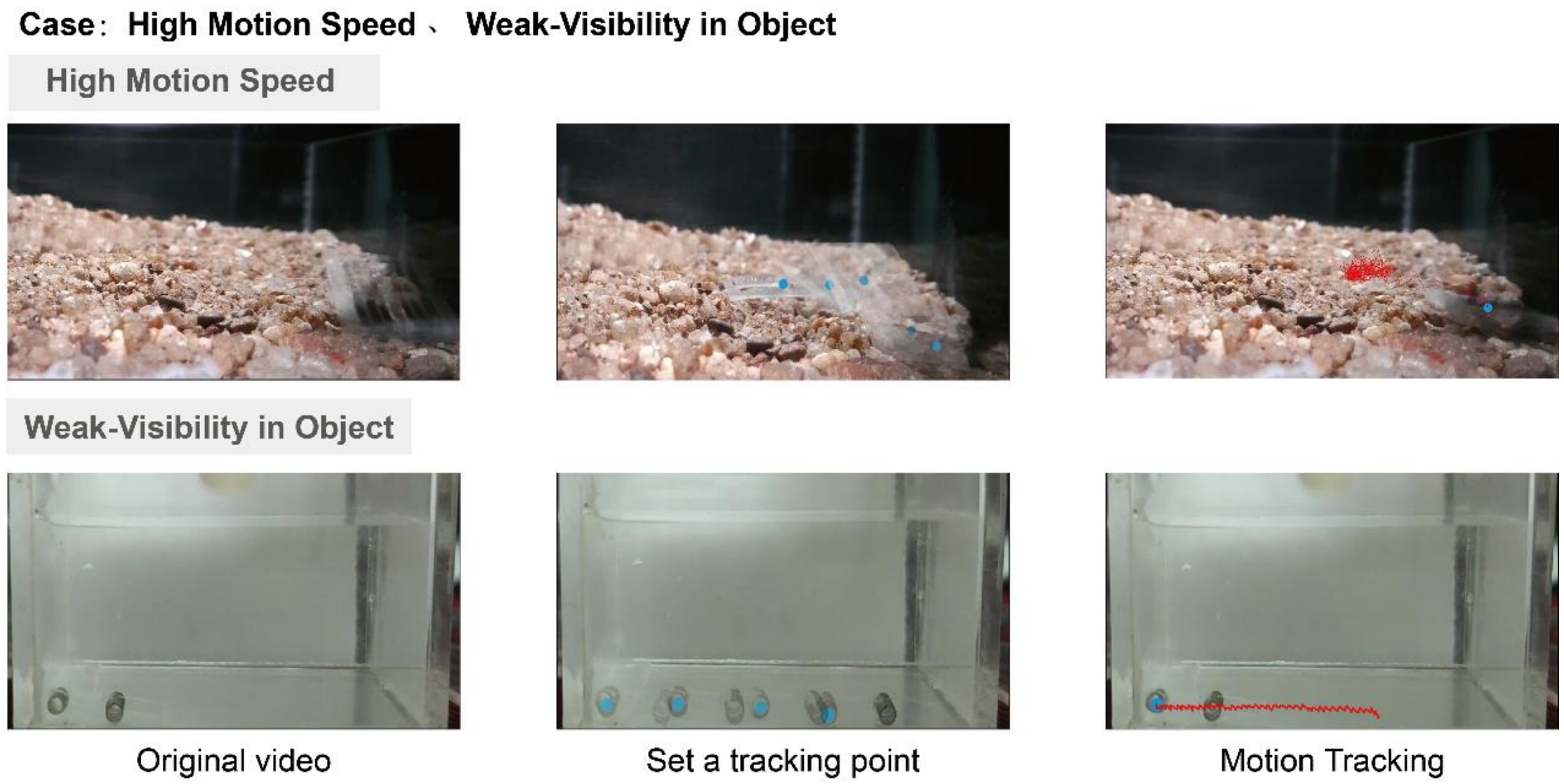

Fig. S21. Path tracking method for high motion speed and weak-visibility in objects. Conventional video tracking methods typically require moderate object speed and clear color contrast between the object and background. In this study, the fish-like robot moves rapidly on land, and its low color contrast due to hydrogel transparency makes standard tracking ineffective. To address this, the locomotion tracking module in After Effects has been employed. A visually distinct marker is placed on a specific part of the robot for tracking. Manual correction is applied when necessary, and the tracked video is exported. Secondary tracking and path rendering are then performed based on the marker to generate a complete path-tracking video.

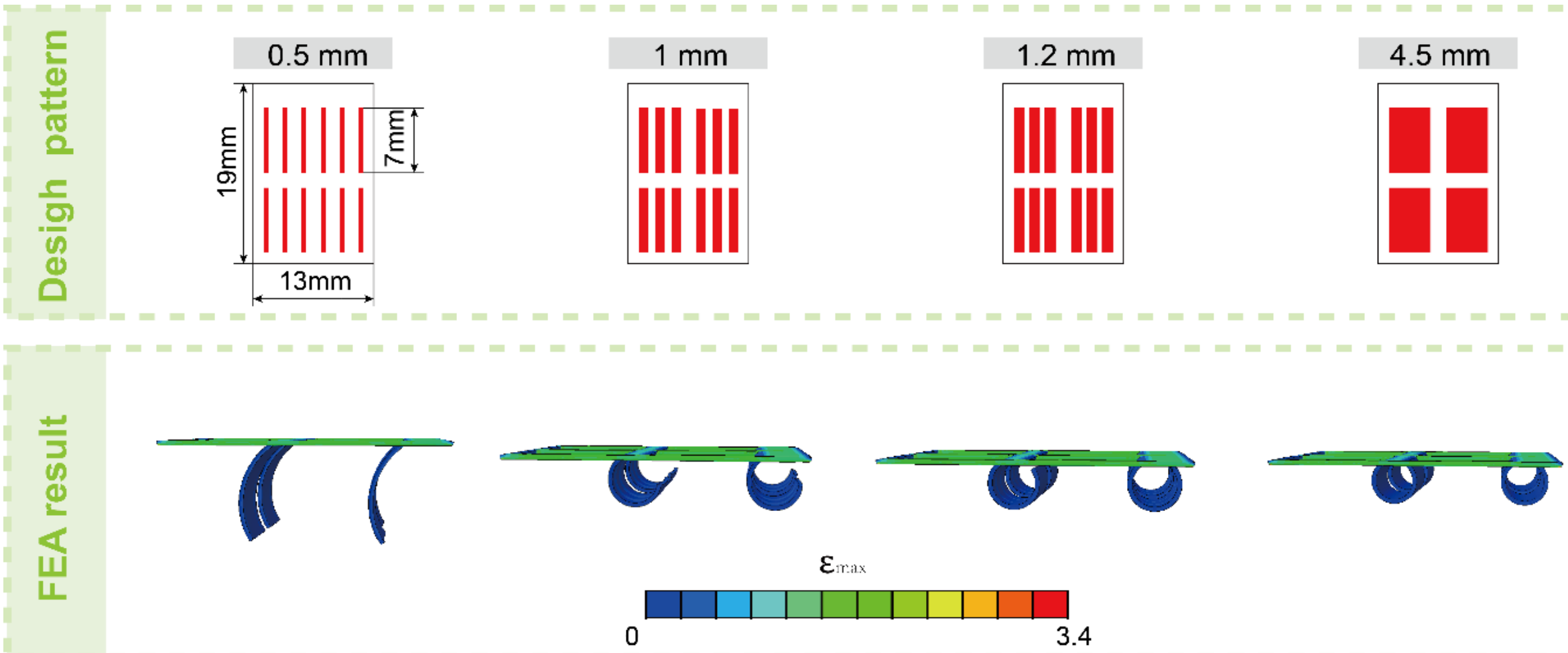

Fig. S22. Design patterns and deformation simulations of four fish-like bioinspired soft robots. Four robots with stripe widths of 0.5 mm, 1 mm, 1.2 mm, and 4.5 mm are designed. Simulation results show that as the stripe width increases, the anisotropic deformation of the hydrogel becomes more obvious, while the bending curvature decreases.

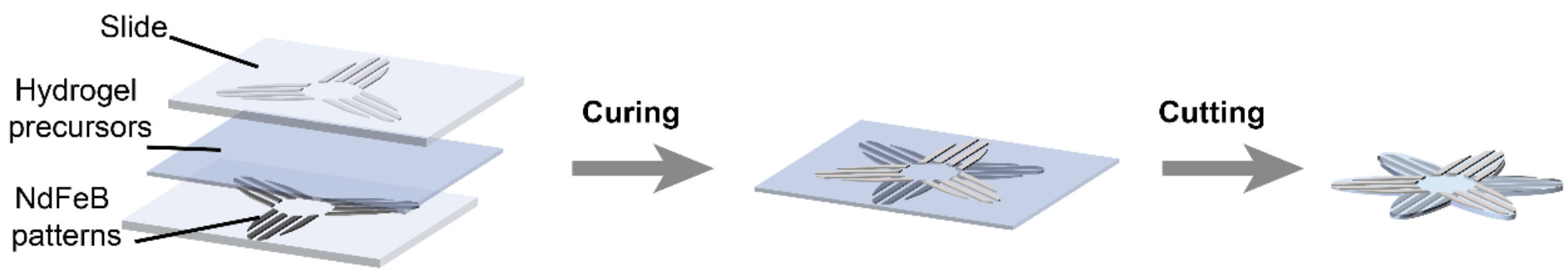

Fig. S23. Fabrication process of the double-sided magnetized skeleton robot. Unlike the single-sided programming method, PET tapes carrying NdFeB patterns are adhered to two separate glass slides. The slides are then aligned face-to-face with the hydrogel precursor filled in between. After curing, PET tape removal, and cutting, the double-sided magnetized skeleton robot is obtained.

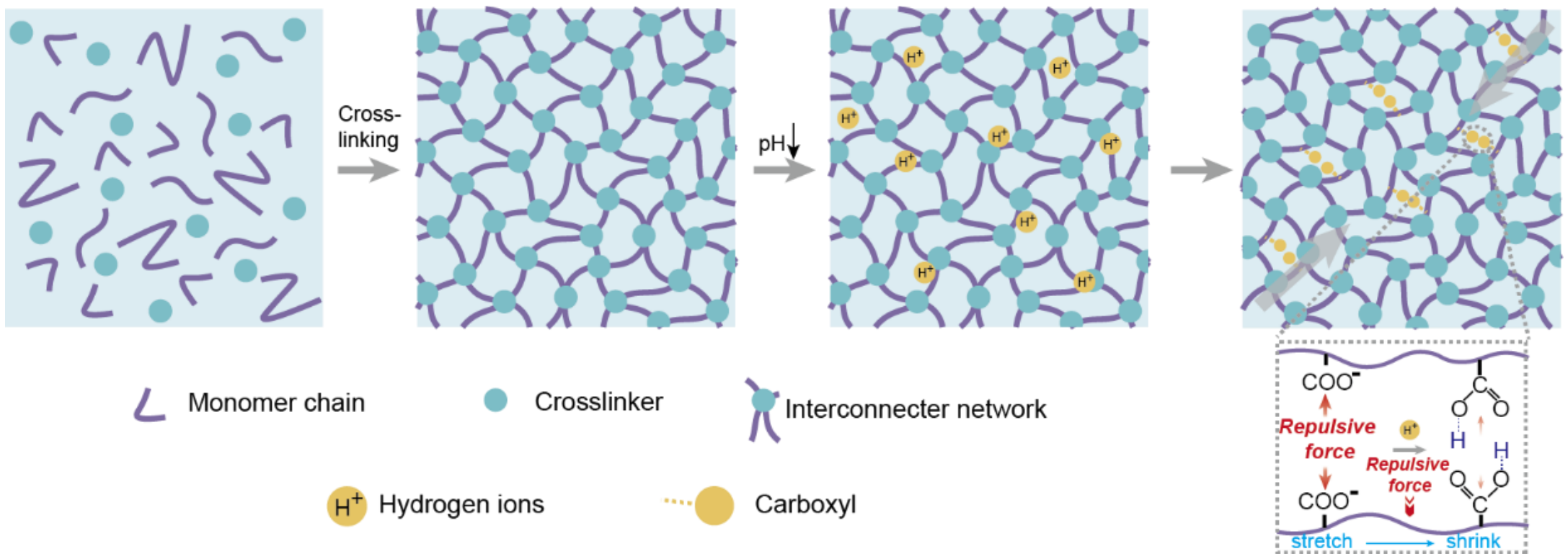

Fig. S24. Mechanism of responsive deformation in AAc-based hydrogel. After incorporation and crosslinking of AAc, carboxylate ions (-COO^{-}) are generated within the hydrogel network, leading to electrostatic repulsion between polymer chains. In an acidic environment, these ions are protonated to neutral carboxylic groups (-COOH), eliminating the repulsion. As a result, the hydrogel contracts in volume and undergoes responsive deformation.

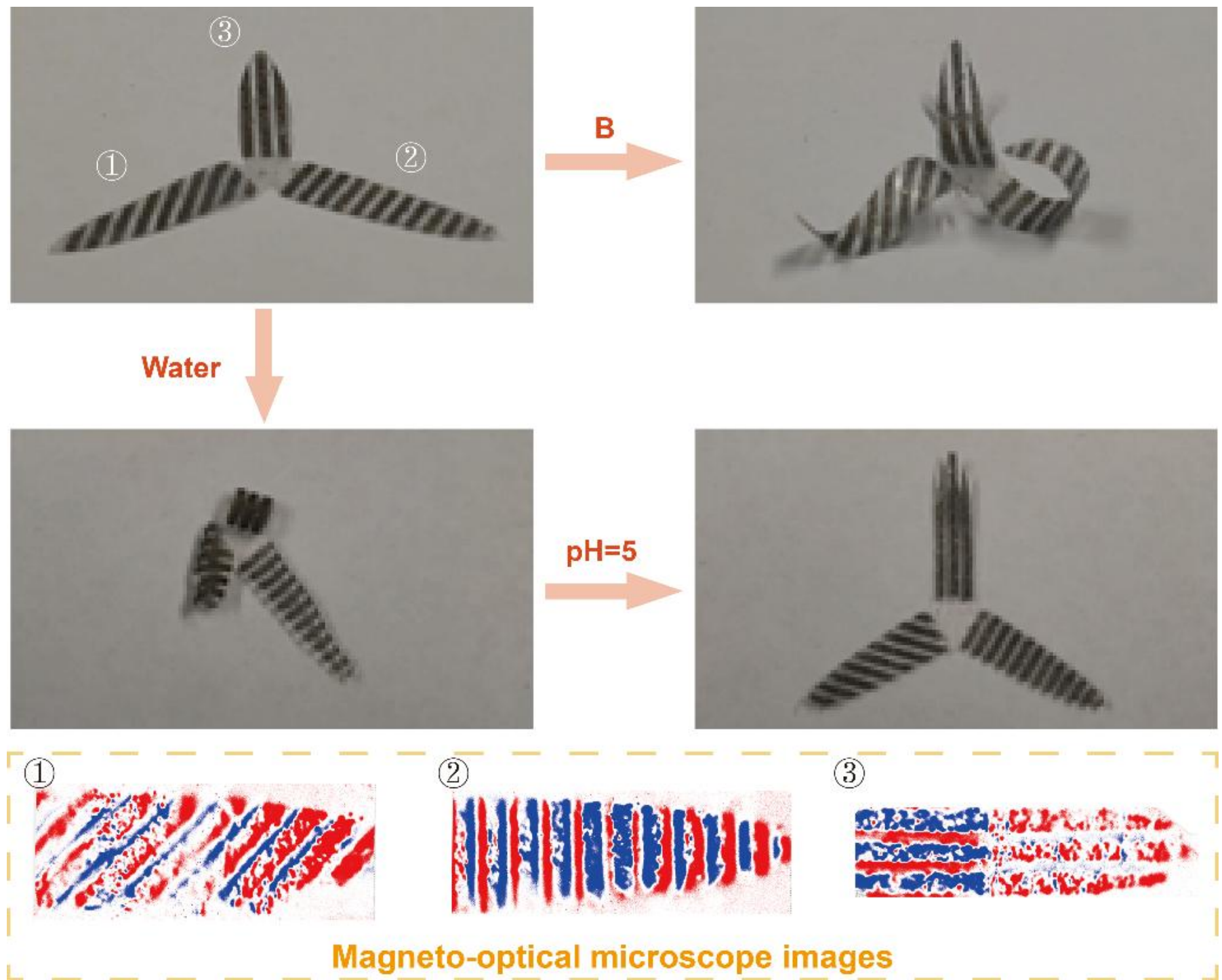

Fig. S25. Integration of multiple magnetic patterns for diverse deformations. The figure shows a trefoil-like robot with three distinct magnetic patterns: Leaf 1 has diagonal stripes, Leaf 2 has horizontal stripes, and Leaf 3 has vertical stripes. Each leaf is magnetized in a different direction. Under an external magnetic field, the leaves deform differently according to their magnetization orientations. Due to the pattern-dependent anisotropic deformation in water, each hydrogel leaf bends uniquely. With AAc groups incorporated, the hydrogel also responds to pH stimuli. At pH 5, the hydrogel contracts; constrained by directional magnetic patterns, the contraction becomes non-uniform, resulting in leaf unfolding.

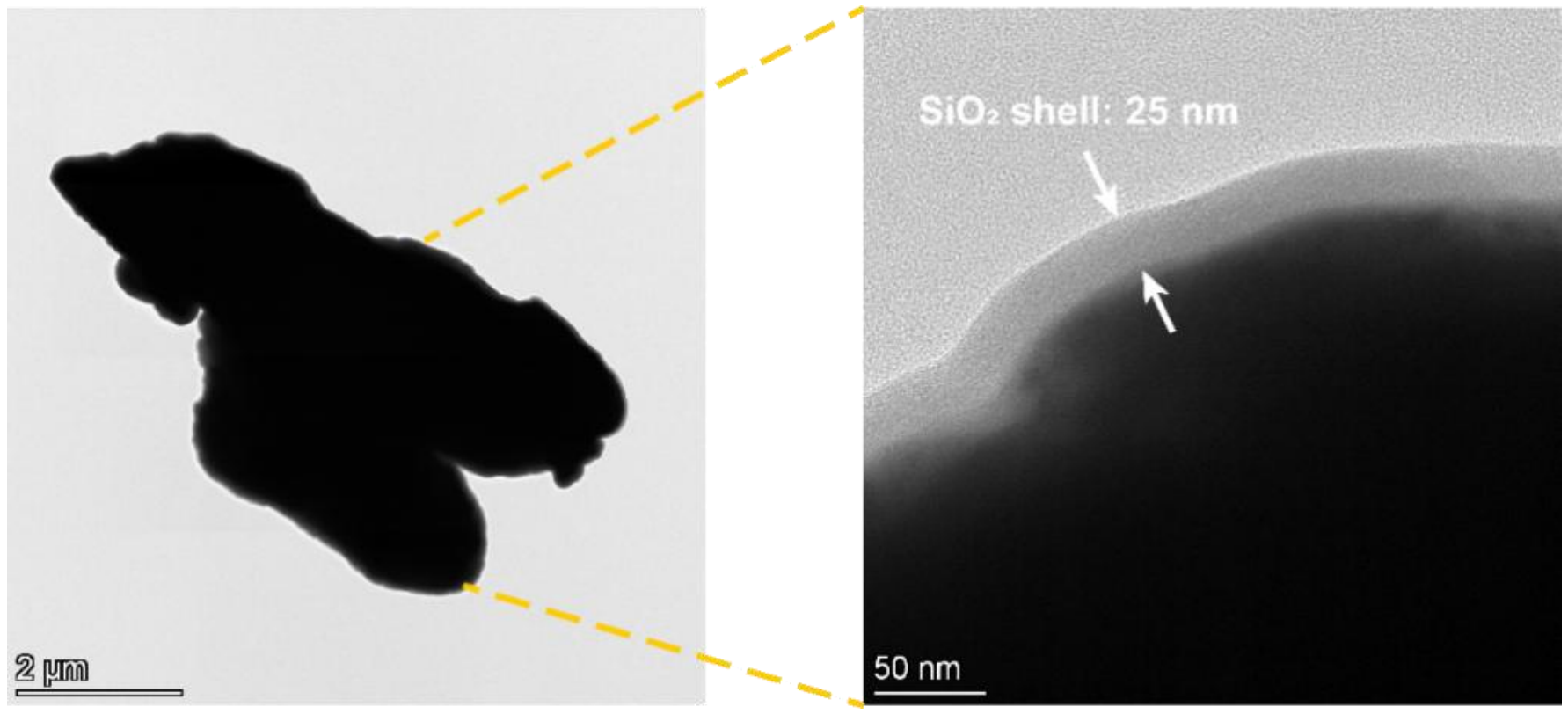

Fig. S26. TEM images of NdFeB microparticles.

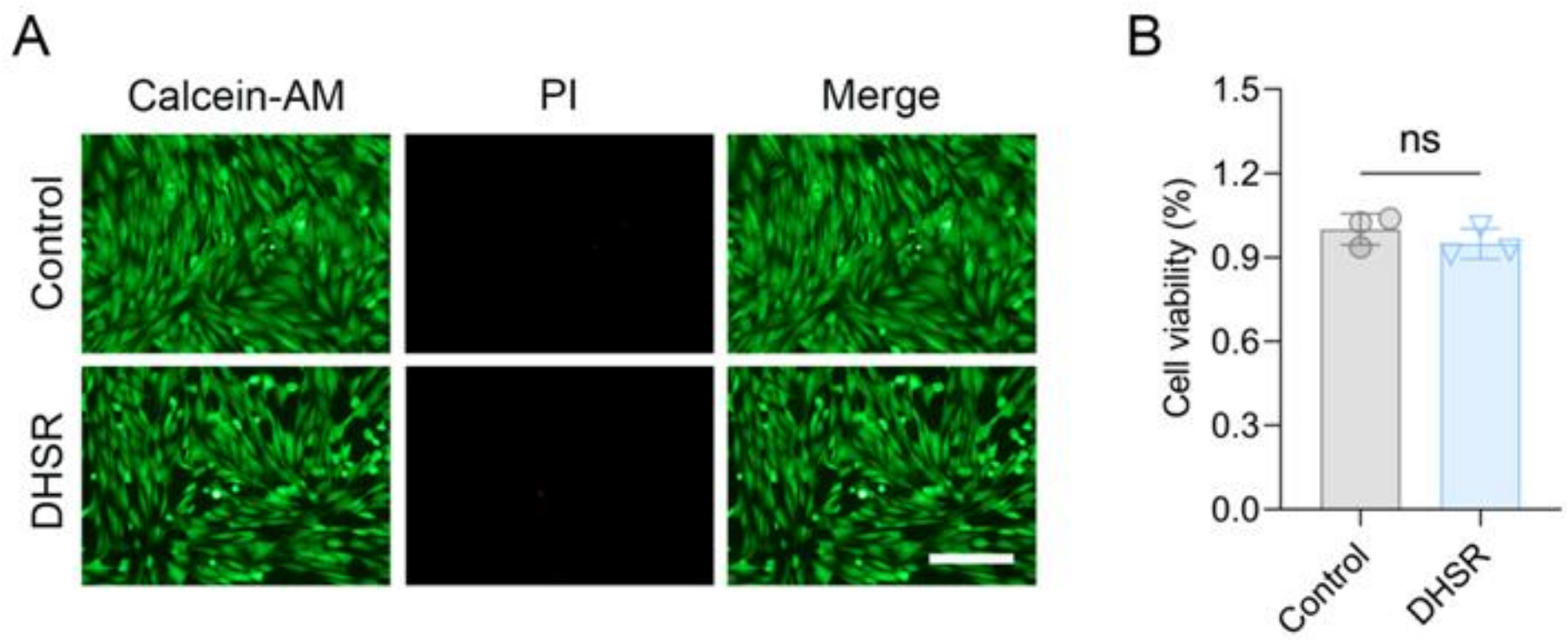

Fig. S27. In vitro biocompatibility evaluation of DHSR leachate. (A) Live/dead staining images of fibroblasts in control and leachate-treated groups at 24 h (live cells in green, dead cells in red). Scale bar: 100 μm. **(B)** CCK-8 assay showing the viability of human dermal fibroblasts cultured with DHSR leachate for 24 h, normalized to the control group.

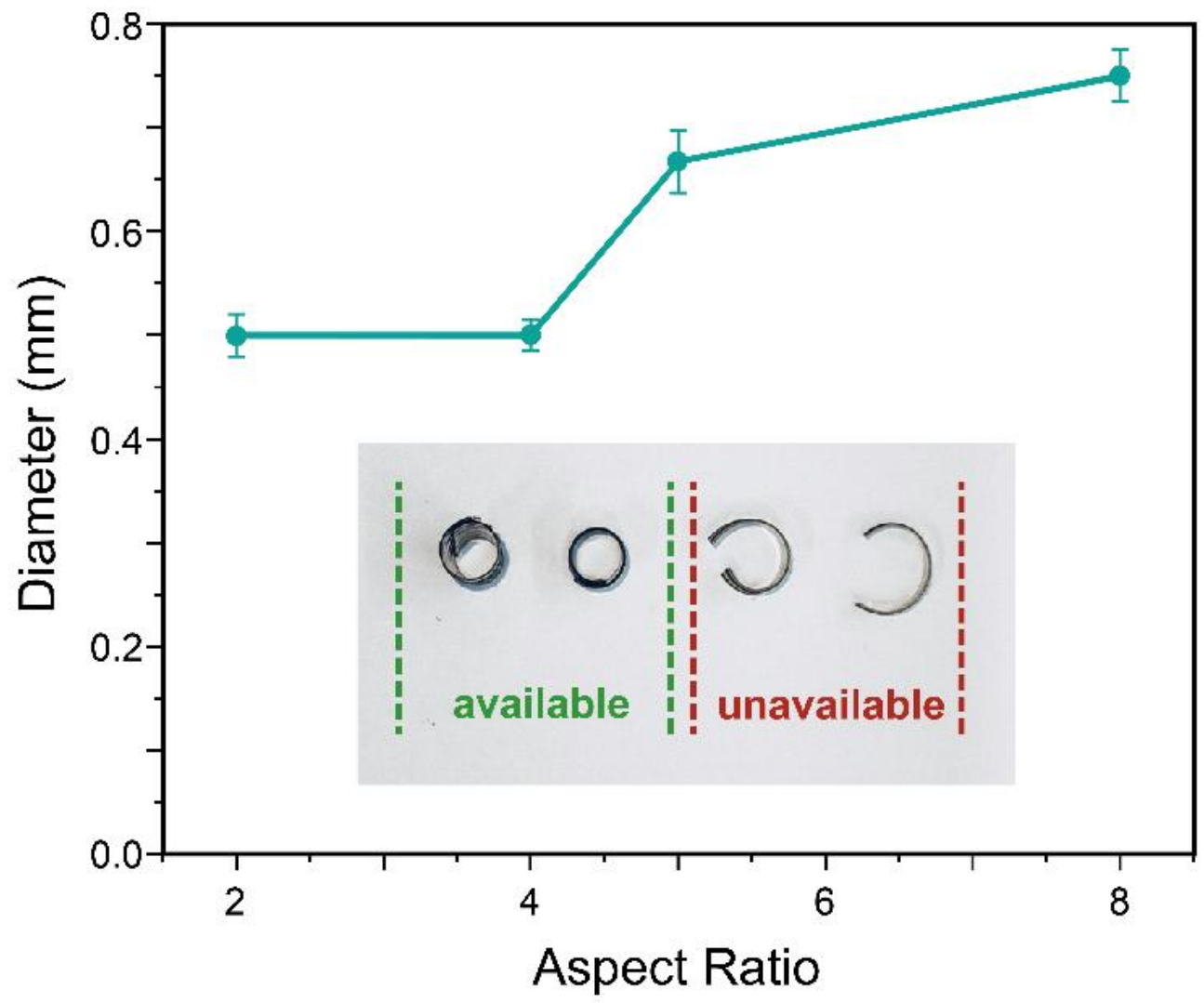

Fig. S28. Effect of hydrogel strip aspect ratio on bending curvature. In this study, 20 mm-long hydrogel strips are employed based on practical considerations, and four aspect ratios are tested for bending behavior. When the hydrogel strip fully curls into a ring, the aspect ratio has little effect on curvature. However, for the hydrogel strip that cannot form a full ring, smaller aspect ratios result in greater bending. This is attributed to the increased number of magnetic stripes with decreasing aspect ratio, which enhances the constraint on uniform swelling and promotes anisotropic deformation. Since the constraining force is uniformly distributed across the width, more stripes lead to a stronger constraint and reduced curvature. Once the hydrogel strip bends into a ring, the constraint balances the force required for bending, minimizing the influence of aspect ratio. Based on these findings, a strip with an aspect ratio of 4 is applied in the medical robot.

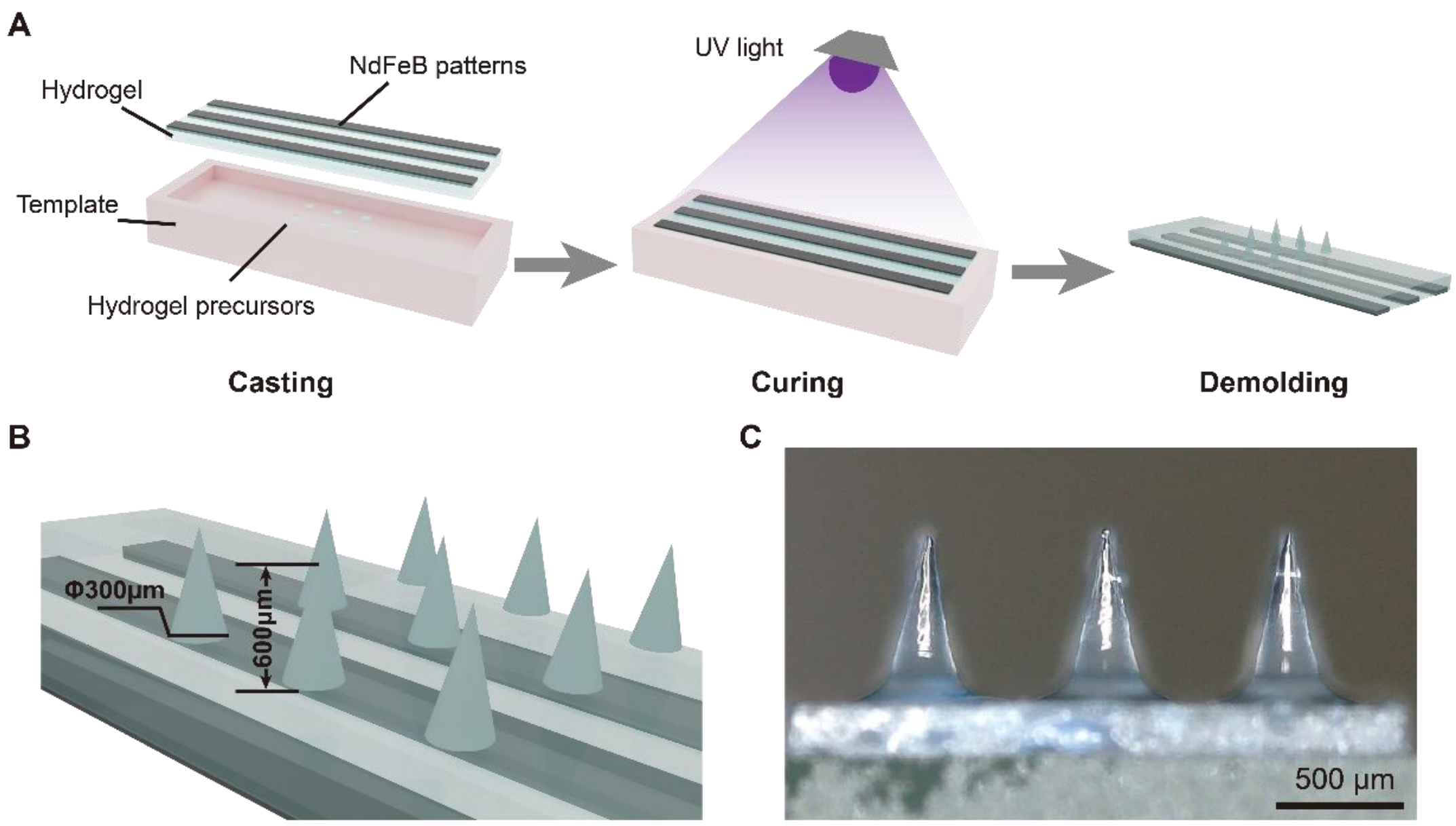

Fig. S29. Fabrication process and microscope image of microneedles in medical robots. (A) Schematic of the microneedle fabrication process: hydrogel precursor or resin precursor is injected into conical cavities of a PDMS mold, and a pre-fabricated hydrogel strip is placed on top. After 60 seconds of curing, the mold is inverted and cured for another 15 seconds. The robot is then removed from the mold, completing the microneedle fabrication. **(B)** Microneedle dimensions: conical microneedles with a diameter of 300 μm and a height of 600 μm. **(C)** Optical microscope image of the microneedles.

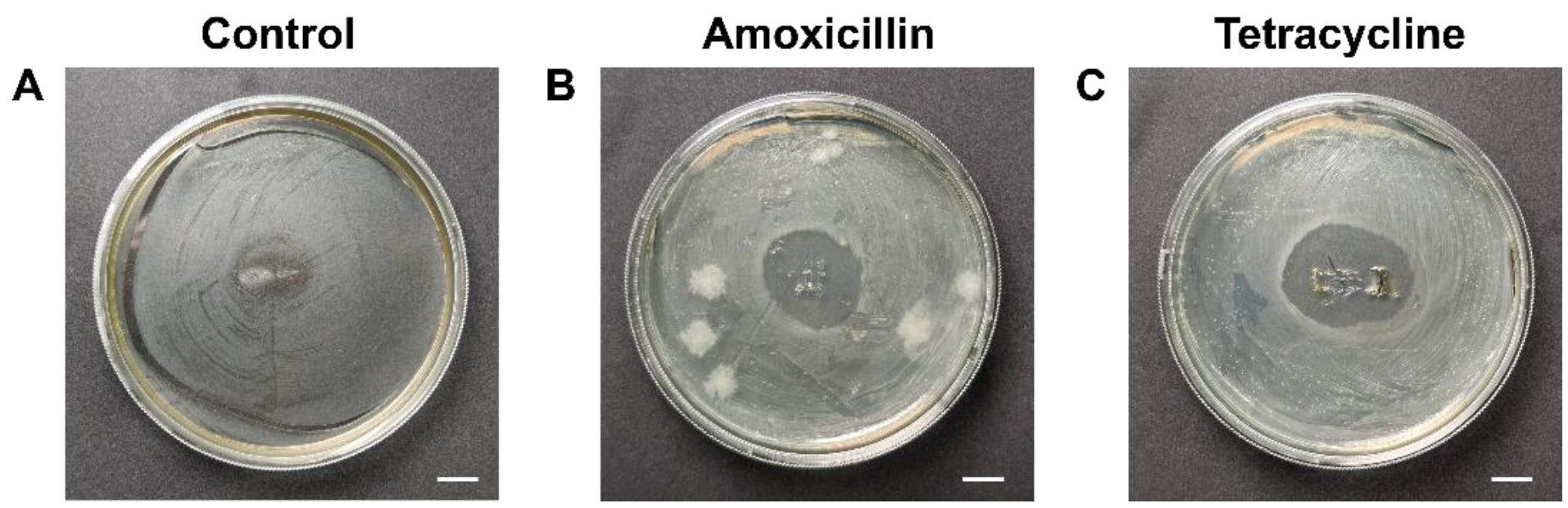

Fig. S30. 24-hour drug-loaded microneedle antibacterial assay. **(A)** Without drug loaded group. **(B)** Amoxicillin-loaded microneedle group. **(C)** Tetracycline -loaded microneedle group. Scale bar: 10 mm.

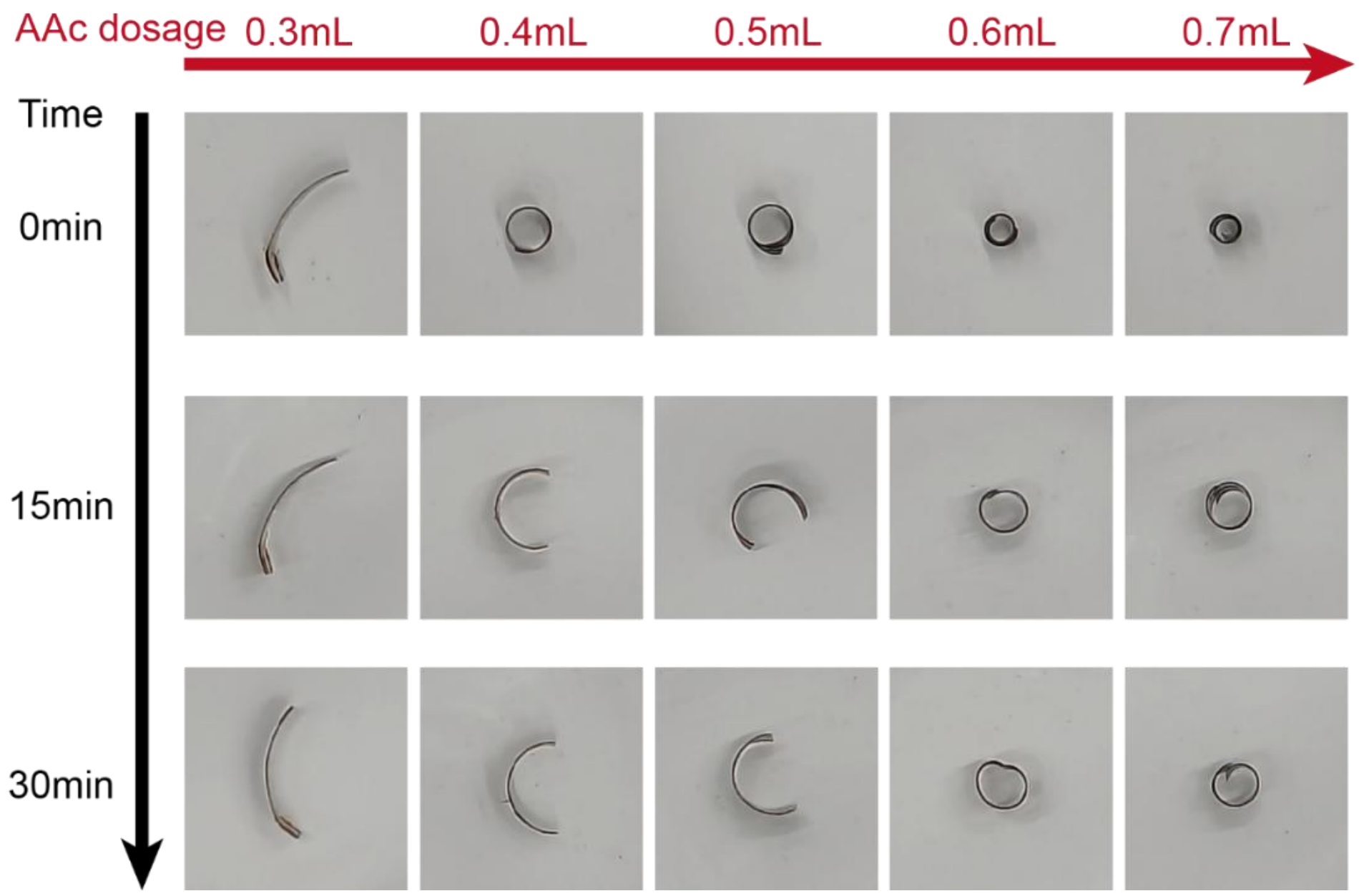

Fig. S31. The pH-responsive deformation of hydrogel-based robots with varying acrylic acid content. Five robot samples are prepared with 0.3 mL, 0.4 mL, 0.5 mL, 0.6 mL, and 0.7 mL of acrylic acid added. After soaking in an acidic environment at body temperature (37°C), the degree of deformation increases with soaking time, while the bending curvature of the hydrogels gradually decreases with increasing acrylic acid concentration. This is attributed to the higher acrylic acid content providing more binding sites during network formation, resulting in a denser network after the same curing time that traps more water molecules, leading to more pronounced deformation. The group with the lowest acrylic acid content could not curl into a ring to effectively protect the microneedles, thus lacking practical value. At 0.4 mL of acrylic acid, the larger unfolding diameter made it difficult to stably lodge in the stomach folds, which is unfavorable for subsequent treatment. Due to soaking time limitations, hydrogels with higher acrylic acid content showed less unfolding, preventing effective exposure of the microneedles, making them unsuitable for medical robots. Based on these comparisons, the hydrogel with 0.5 mL of acrylic acid is selected.

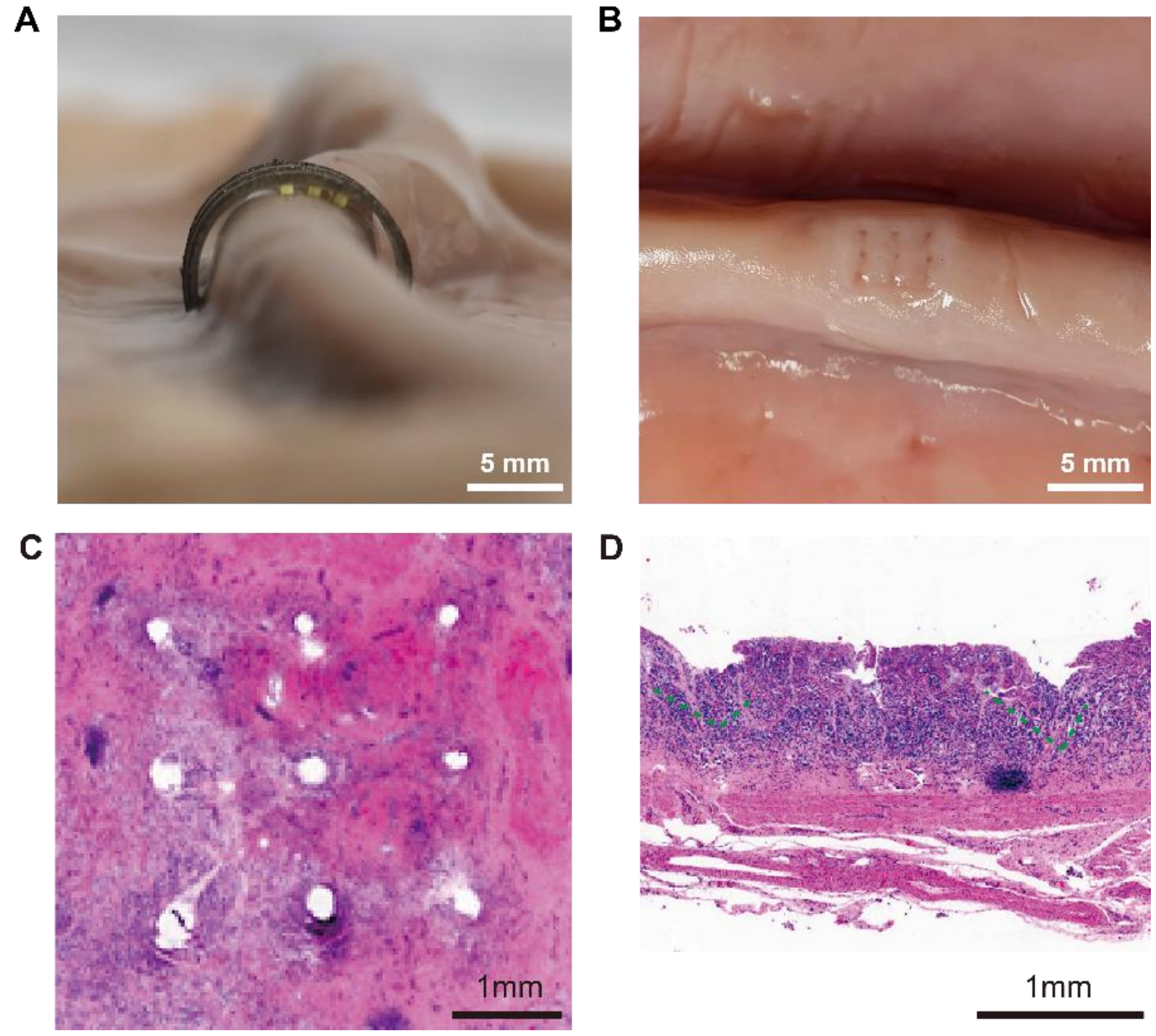

Fig. S32. Microneedles penetrate the gastric wall surface. (A) Microneedle-loaded DHSR penetrates the gastric wall surface. **(B)** Microneedle-loaded DHSR penetrates the gastric wall surface. Top-view **(C)** and cross-sectional **(D)** results of H&E-stained tissue sections.

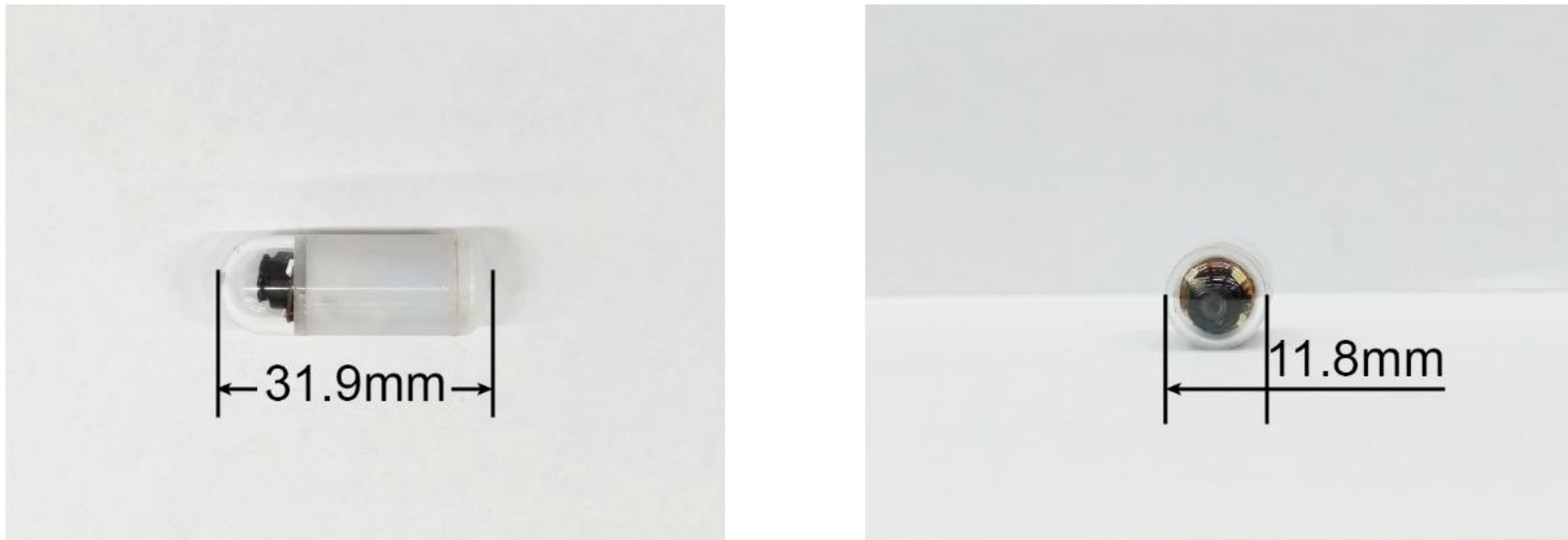

Fig. S33. Size information of the capsule endoscope. The experimental capsule endoscope measures 31.9 mm in length with a maximum diameter of 11.8 mm, allowing easy passage through the oral cavity.

Table S1. Technical summary of previous studies

Article	Robot fabrication method	Magnetic programming method	Material matrix and response characteristics	NdFeB patterning	Multi-curvature / >360° deformation	Avoidance of magnetic-particle sedimentation	Decoupling of locomotion and function
14.Science Advances 6, eabc6414 (2020).	Mold-cured CrO_2/PDMS films are shaped into soft machines.	Curie-temperature thermal reprogramming under local/global magnetic fields.	**Silicone elastomer matrix** Magnetically driven rapid and reversible 2D/3D shape morphing; enables walking, grasping, and metamaterial deformation.	**N/A** CrO_2 is uniformly mixed into PDMS, without magnetic-particle patterning.	**Partial**. Complex curved deformation is achieved, but the deformation curvature does not exceed 360°	**No** Sedimentation is not discussed.	**Partial** Reprogramming enables differentiated deformation and induces functional decoupling.
19. Science Advances 8,eabn8932 (2022).	Wax-patterned NdFeB/PEI films are transferred into adhesive stickers and integrated with functional modules.	Assembly of pre-magnetized discrete/continuous NdFeB modules.	**PET matrix** Magnetically driven 2D and 3D origami-like and multi-curved deformation; enables multimodal locomotion and integration of functional modules.	**Yes** NdFeB is assembled onto tape in patterned form.	**No** The deformation curvature does not exceed 360°	**Yes** Particles are fixed, avoiding sedimentation.	**Partial** Decoupling is achieved by integrating functional modules, not by local structural deformation.
13. Nature 554, 81–85 (2018).	Ecoflex/NdFeB magnetoelastic sheet with hydrophobic surface.	Template-assisted harmonic magnetization programming.	**Silicone elastomer matrix** Magnetically driven multimodal locomotion such as swimming, liquid-surface crawling, rolling, walking, in-pipe crawling, and jumping.	**No** NdFeB particles are uniformly mixed into Ecoflex, without magnetic-particle patterning.	**No** Complex curved deformation is present, but the deformation curvature does not exceed 360°	**No** Sedimentation is not discussed.	**Partial** Magnetically driven multi-deformation capability enables multiple locomotion modes.
10. Nature 558, 274–279 (2018).	Direct ink writing of NdFeB/fumed-silica/silicone ink into soft structures.	Nozzle-field particle alignment encodes ferromagnetic domains during printing.	**Silicone elastomer matrix** Magnetically driven rapid and reversible 3D deformation; enables jumping motion.	**No** NdFeB particles are uniformly mixed into silicone, without magnetic-particle patterning.	**Partial** Complex curved deformation is achieved, but the deformation curvature does not exceed 360°	**Yes** Silica increases yield stress and suppresses sedimentation.	**Partial** Decoupling is achieved by integrating functional modules, not by local structural deformation.
30. Science Robotics 4, eaax7329 (2019).	Printed/extruded or injection-molded PDMS/TPU-NdFeB body with grafted hydrogel skin.	Strong-pulse magnetization of the NdFeB composite to form steerable domains.	**Silicone/TPU matrix** Submillimeter continuum robot with omnidirectional steering, low friction, and potential integration with optical-fiber delivery.	**No** NdFeB particles are uniformly mixed into PDMS/TPU, without magnetic-particle patterning.	**Partial** Complex curved deformation is achieved, but the deformation curvature does not exceed 360°	**Yes** Thixotropic ink suppresses phase separation.	**Partial** Magnetic navigation is decoupled from the optical-fiber function.
21. Nat Commun 15, 10442 (2024).	Uniform-field-assisted DLP stereolithography of NdFeB photocurable resin into thin-walled hollow robots.	In situ particle orientation and UV curing under 3D uniform magnetic fields.	**Resin matrix** Magnetically driven thin-walled structures enable peristalsis, unidirectional infusion, pumping, and drug release.	**No** NdFeB particles are uniformly mixed into photocurable resin, without magnetic-particle patterning.	**Partial** Complex curved deformation is achieved, but the deformation curvature does not exceed 360°	**Yes** Small particles plus fumed silica provide stability for 8 h.	**Partial** Magnetically driven motion is decoupled from deformation-mediated drug release.
11. Science Robotics 4,	Premagnetized NdFeB/UV-resin slurry	DLP selective curing after 3D	**Resin matrix**	**No**	**Partial**	**No**	**Partial**

eaav4494 (2019).	is UV-lithographically patterned into planar microrobots.	reorientation of premagnetized particles.	Magnetically driven origami and multiaxial bending enable locomotion and grasping deformation.	NdFeB particles are uniformly mixed into photocurable resin, without magnetic-particle patterning.	Multiaxial and 180 deg large bending are achieved, but the deformation curvature does not exceed 360°	Sedimentation is not discussed.	Locomotion, grasping, and release functions are decoupled.
12. Science Robotics 6, eabf0112 (2021).	Mold-cast magnetic/nonmagnetic polymer voxels are jig-assembled with elastomeric adhesive.	3D magnetization programmed by arranging voxels with prescribed magnetic moments.	**Silicone elastomer matrix**<br>Magnetically driven continuous deformation of microstructures enables negative Poisson's ratio, pumping, biopsy, transport, and anchoring deformation.	**No**<br>NdFeB particles are uniformly mixed into silicone, without magnetic-particle patterning.	**Partial**<br>Complex curved deformation is achieved, but the deformation curvature does not exceed 360°	**Partial**<br>Voxels are cured before assembly, indirectly avoiding sedimentation.	**Partial**<br>Decoupling is achieved by integrating functional modules, not by local structural deformation.
5.Proceedings of the National Academy of Sciences 121,e2320386121(2024).	LIG is transferred onto PDMS-NdFeB elastomer; alginate is electro-crosslinked/dried into xerogel, then laser-engraved.	Laser engraving programs multistimulus regions; NdFeB-PDMS layer enables magnetic locomotion.	**LIG-PDMS matrix**<br>Humidity, thermal, and magnetic responses enable multistep deformation, logic switching, and circuit repair.	**No**<br>NdFeB particles are uniformly mixed into PDMS, without magnetic-particle patterning.	**Yes**<br>Complex helical curved-surface deformation is achieved, and the deformation curvature exceeds 360°	**No**<br>Sedimentation is not discussed.	**Yes**<br>Different stimuli independently control deformation, enabling locomotion-function decoupling.
1. Science Robotics 6, eabd5483 (2021).	Conductive photothermal hydrogel prepared by ice-templated UV/cryo-polymerization.	No magnetic programming; porous conductive networks support actuation and sensing.	**Hydrogel matrix**<br>Light/thermal contraction and bending; enables strain and pressure sensing.	**N/A**<br>No magnetic particles are mixed in.	**No**<br>Non-magnetically driven complex curved-surface deformation.	**N/A**<br>Sedimentation is not discussed.	**Partial**<br>Sensing and actuation are decoupled, but the deformation decoupling is not magnetically driven.
44. Nat. Synth 4, 642–654 (2025).	Photodosage-printed NdFeB hydrogel films swell into temporary 3D shapes and are pulse-magnetized.	Template-free inverse magnetic-domain programming via swelling-assisted magnetization.	**Hydrogel matrix**<br>Magnetically driven complex curved-surface deformation; also provides solvent/water response and pH-triggered drug release.	**No**<br>NdFeB particles are uniformly distributed in the hydrogel, and the hydrogel can be patterned.	**Yes**<br>Complex helical curved deformation is achieved, and the deformation curvature exceeds 360°	**Yes**<br>PVP increases viscosity and prevents sedimentation.	**No**<br>The focus is complex curved-surface deformation.
16. Sci. Robot. 5, eabb9822 (2020).	Ni nanowires are aligned in photoresponsive hydrogel monomers and fixed by photopolymerization.	Static-field alignment fixes Ni nanowire magnetic anisotropy.	**Hydrogel matrix**<br>Photocontraction/bending works with magnetic torque to enable underwater locomotion, grasping, and release.	**No**<br>Oriented Ni nanowires are uniformly mixed into hydrogel precursor; but there is no magnetic-particle patterning.	**Partial**<br>Curved deformation is achieved, but the deformation curvature does not exceed 360°	**Partial**<br>Prepolymerization increases gel-monomer viscosity and reduces aggregation and sedimentation of Ni nanowires.	**Yes**<br>Light-driven actuation controls robot morphology, while magnetic actuation controls locomotion.
This work	Magnetized NdFeB patterns are transferred from PI/PET tape and embedded in UV-cured hydrogel; responsive layers/microneedles can be integrated.	Preset NdFeB skeleton and hydrogel anisotropy are independently programmed.	**Hydrogel matrix**<br>Magnetically driven multimodal locomotion and local hydrogel responses to water, acid, temperature, and ions; enables environmental adaptation, motion-sensing-action decoupling, selective drug release, and targeted gastric therapy.	**Yes**<br>The patterned NdFeB magnetic skeleton can be efficiently transferred into hydrogel and arranged spatially on both sides.	**Yes**<br>Dual programming enables complex 3D multi-curvature, helical/ring-shaped, and environmentally adaptive deformation, with deformation curvature exceeding 360°	**Yes**<br>The directly embedded NdFeB skeleton effectively avoids sedimentation and aggregation.	**Yes**<br>Magnetically driven locomotion and environmental sensing are induced by different local structural deformations, enabling locomotion-function decoupling.

Table S2. Technical summary of previous studies

Shape		"S" shape	In-plane	Perpendicular
Magnetizing direction		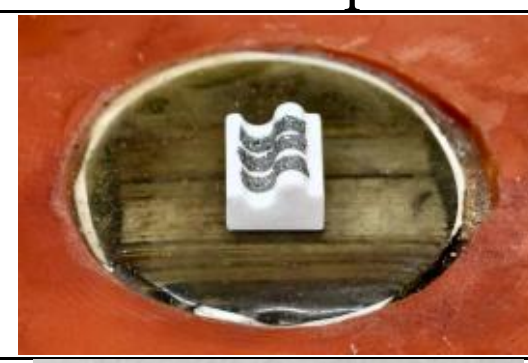	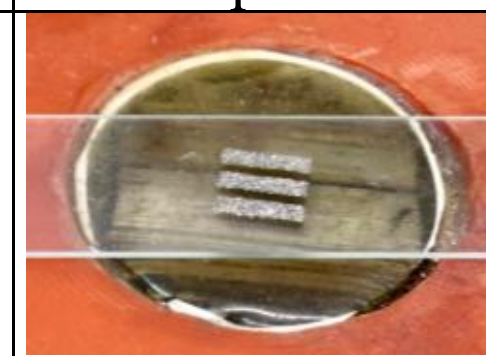	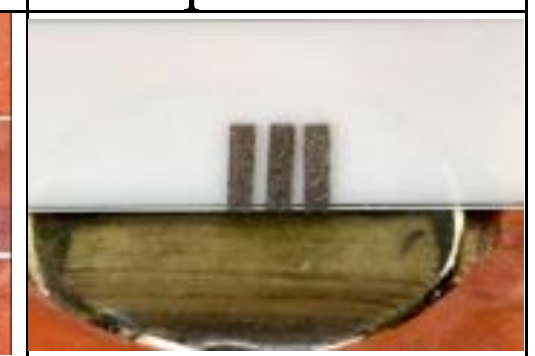
150 μm	Optical images	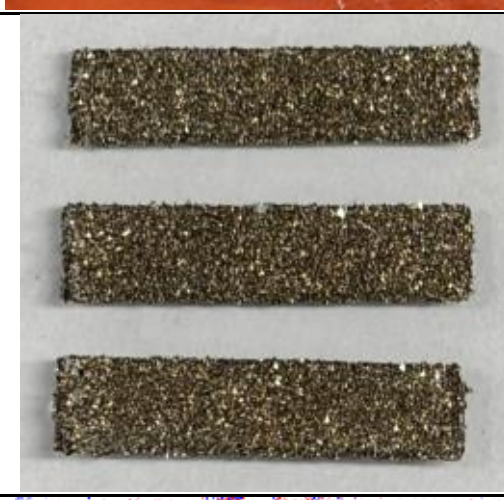	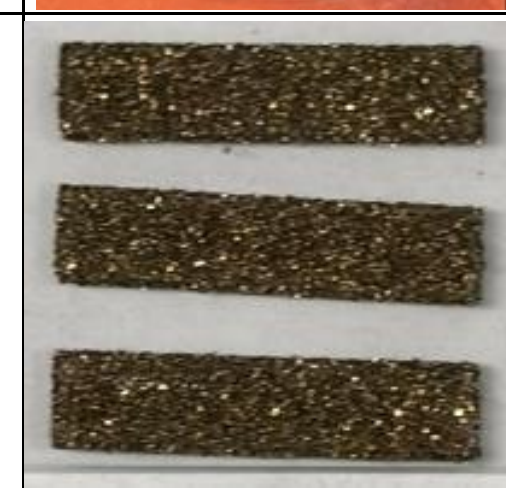	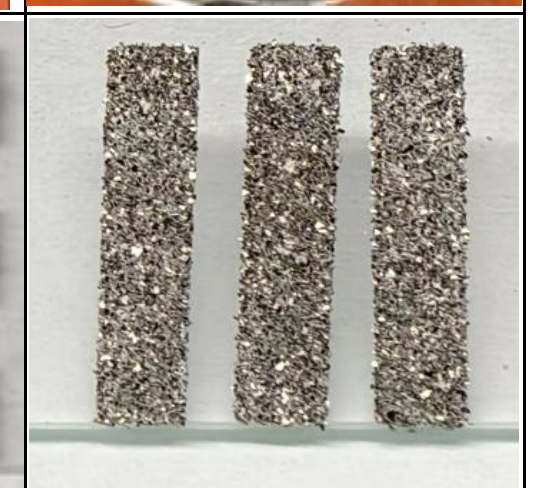
	Magneto-optical microscope images	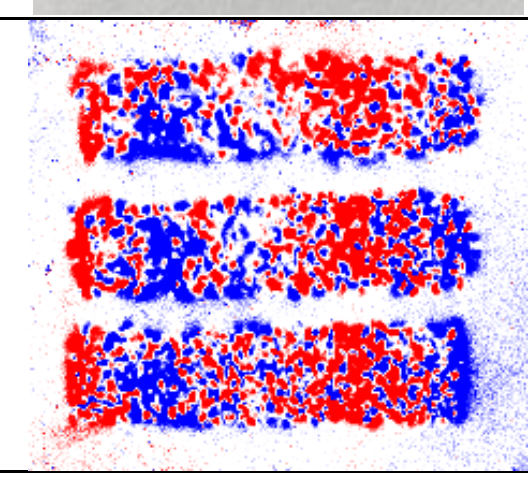	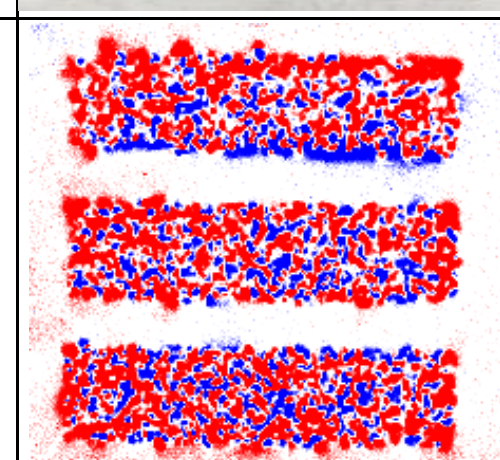	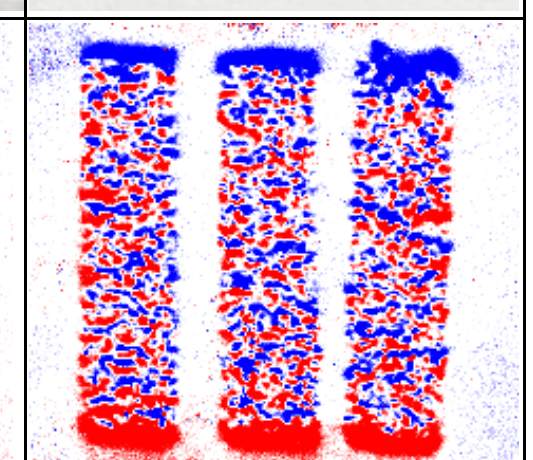
75 μm	Optical images	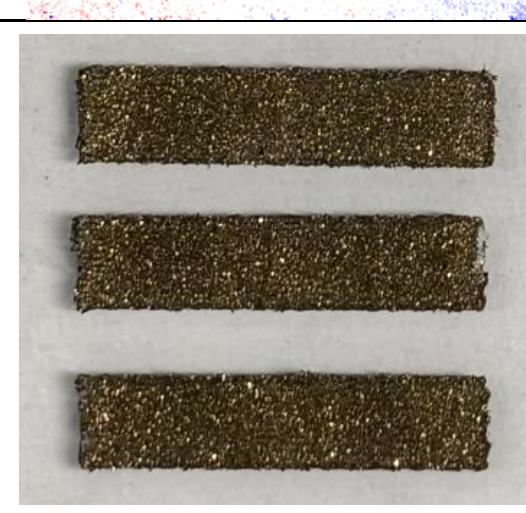	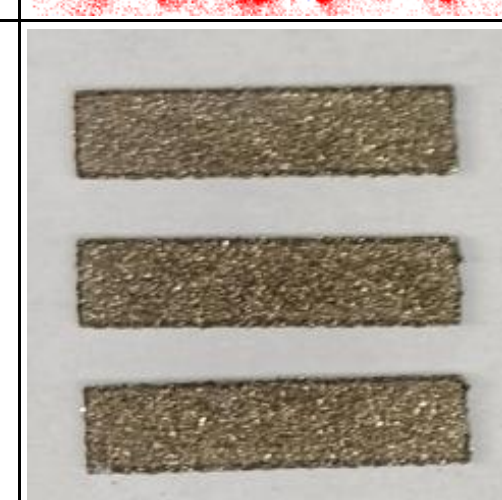	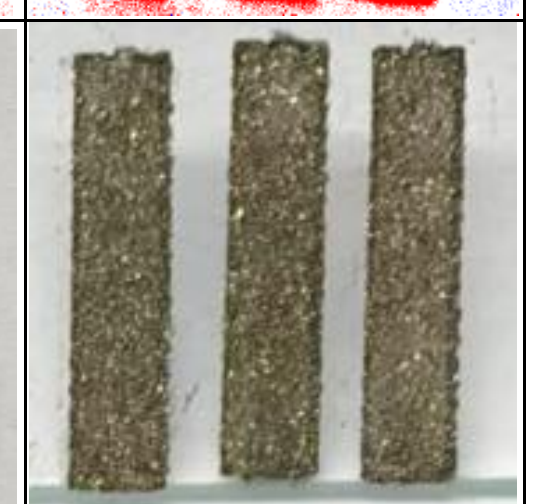
	Magneto-optical microscope images	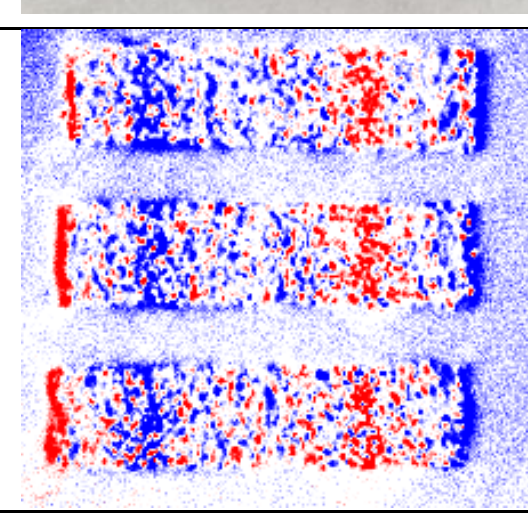	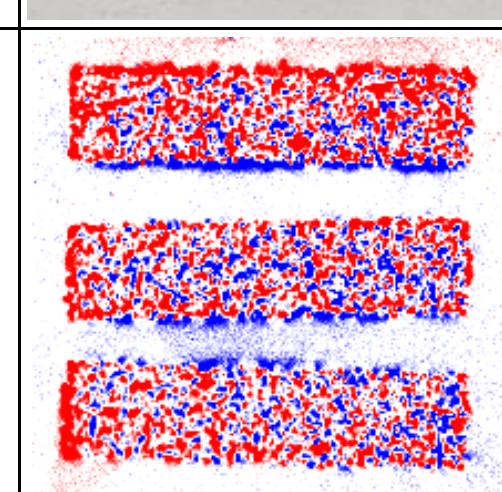	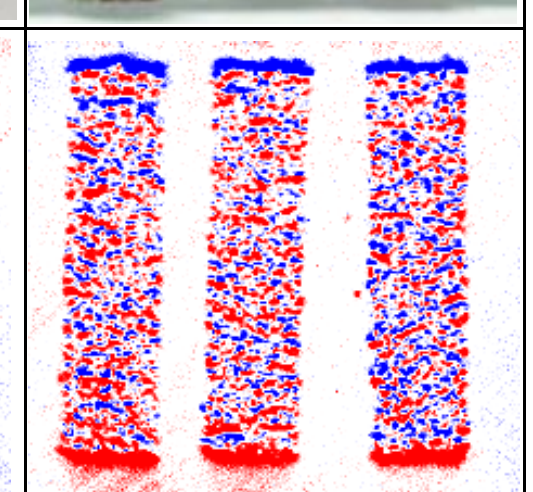
38 μm	Optical images	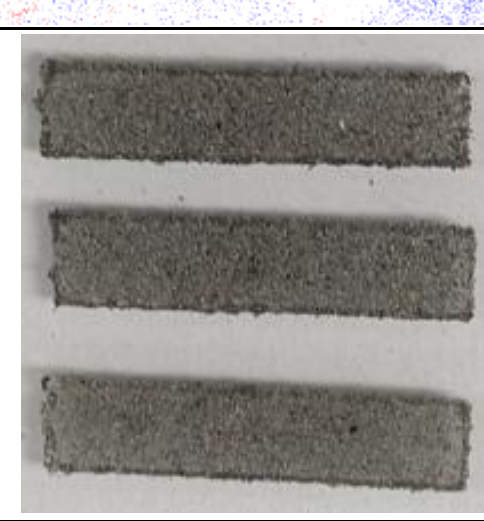	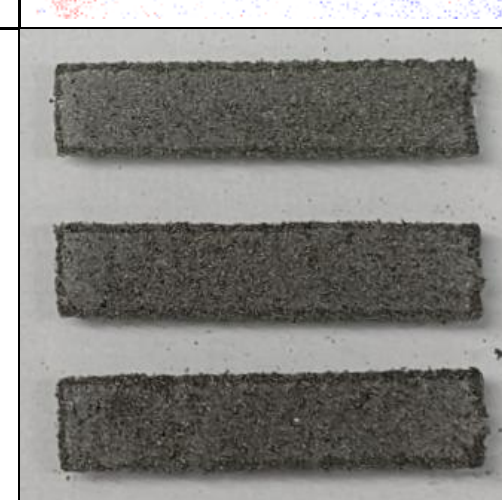	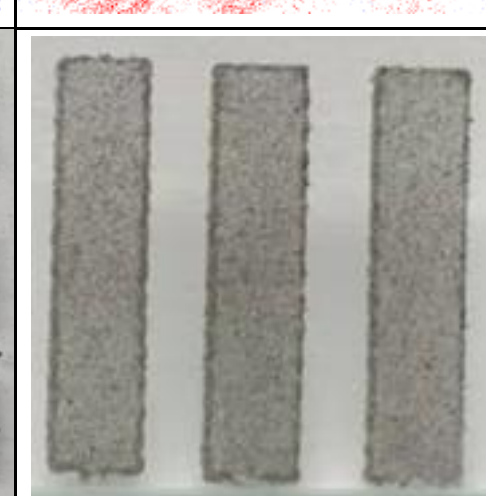
	Magneto-optical microscope images	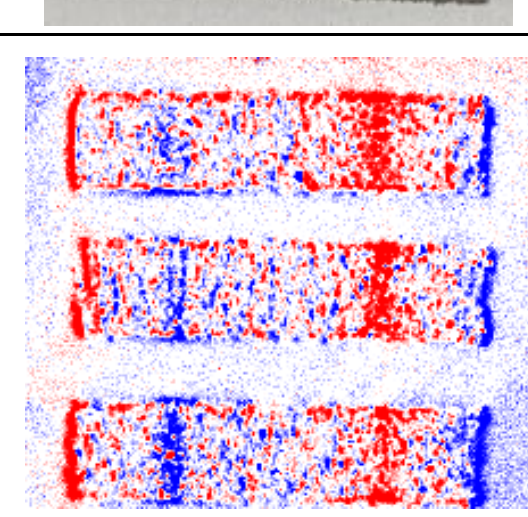	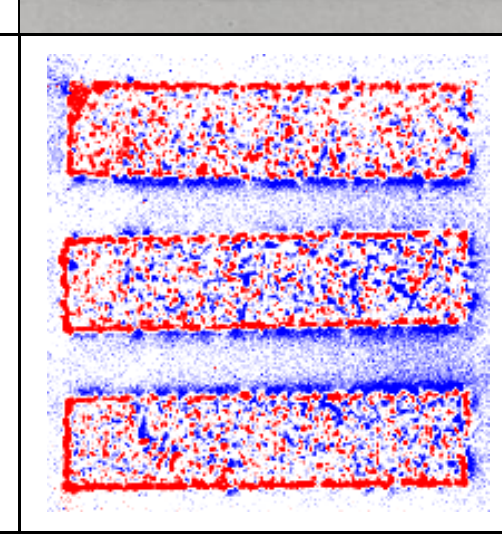	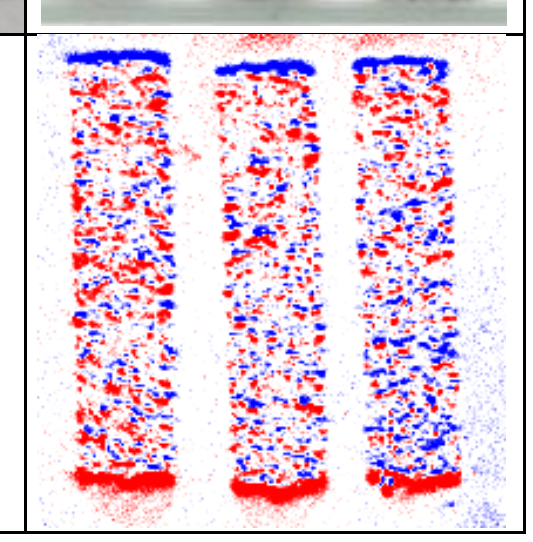

Table S3. Reproducibility test of 150μm NdFeB microparticles

Sample	1	2	3	4	5	6	7	8	9
Optical images	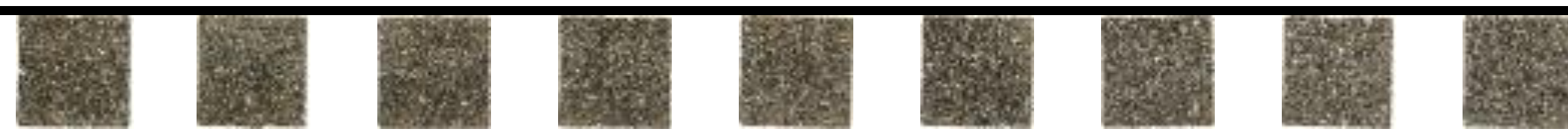								
Magneto-optical microscope images	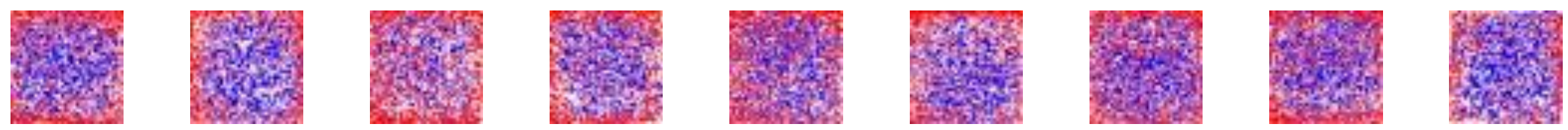								
Weight/g	0.0291	0.0284	0.0281	0.0282	0.0292	0.0286	0.0283	0.0286	0.0294

Table S4. Reproducibility test of 75μm NdFeB microparticles

Sample	1	2	3	4	5	6	7	8	9
Optical images	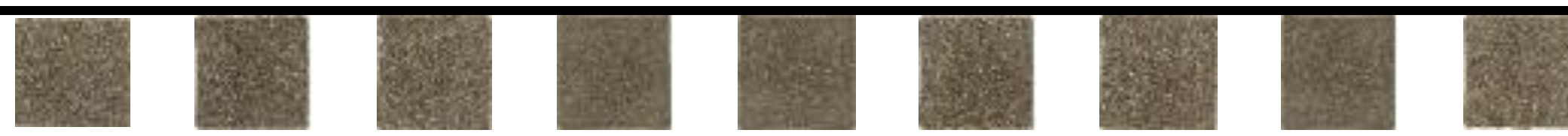								
Magneto-optical microscope images	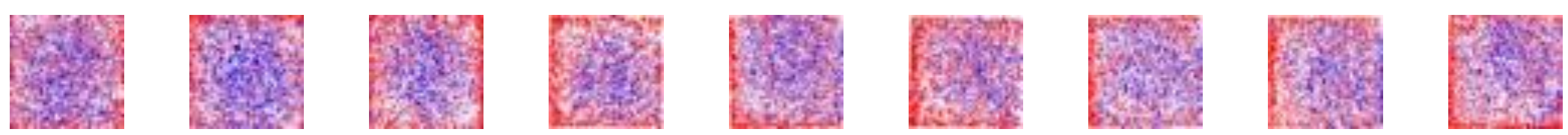								
Weight/g	0.0227	0.0221	0.0225	0.0223	0.0226	0.0229	0.0225	0.0224	0.0220

Table S5. Reproducibility test of 38μm NdFeB microparticles

Sample	1	2	3	4	5	6	7	8	9
Optical images	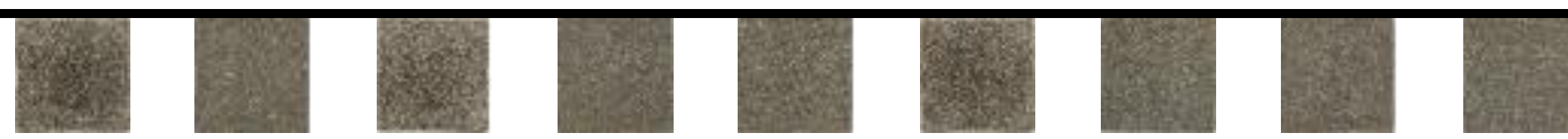								
Magneto-optical microscope images	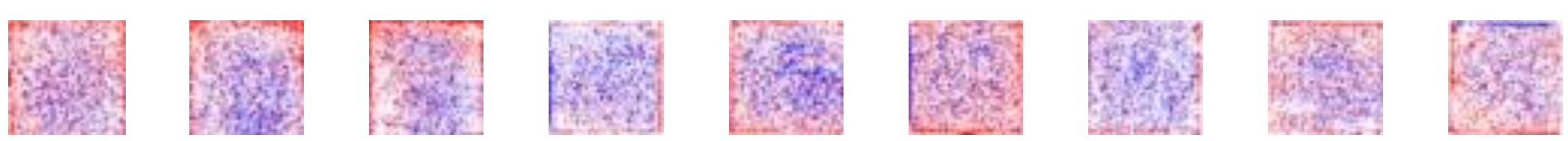								
Weight/g	0.0201	0.0195	0.0190	0.0202	0.0192	0.0196	0.0192	0.0199	0.0193

Movie S1.
The assembly of DHSR with roll-to-roll device

Movie S2.
Transfer experiment of NdFeB particles

Movie S3.
Adaptive locomotion of four-fin soft robot

Movie S4.
Experiment of single cargo releasing

Movie S5.
The DHSR with sequential actuation for multi-site cargo releasing

Movie S6.
The DHSR for the treatment of gastric diseases

Movie S7.
Integrated therapy combining the DHSR and capsule endoscopy

Movie S8.
In vivo animal experiments of a diagnostic-therapeutic platform

Data S1. (.xlsx format).
Source data for the peeling force test of three same DHSR samples in figure S9B.
